# Insights into the chemistry of the amphibactin–metal (M^3+^) interaction and its role in antibiotic resistance

**DOI:** 10.1038/s41598-020-77807-3

**Published:** 2020-12-03

**Authors:** Vidya Kaipanchery, Anamika Sharma, Fernando Albericio, Beatriz G. de la Torre

**Affiliations:** 1 KwaZulu-Natal Research Innovation and Sequencing Platform (KRISP), School of Laboratory Medicine and Medical Sciences, College of Health Sciences, University of KwaZulu-Natal, Durban, 4041 South Africa; 2 Peptide Science Laboratory, School of Chemistry and Physics, University of KwaZulu-Natal, Durban, 4001 South Africa; 3 CIBER-BBN, Networking Centre on Bioengineering, Biomaterials and Nanomedicine, and Department of Organic Chemistry, University of Barcelona, 08028 Barcelona, Spain; 4 Institute for Advanced Chemistry of Catalonia (IQAC-CSIC), 08034 Barcelona, Spain

**Keywords:** Density functional theory, Medicinal chemistry

## Abstract

We have studied the diversity and specificity of interactions of amphibactin produced by Vibrio genus bacterium (*Vibrio* sp. HC0601C5) with iron and various metal ions in + 3 oxidation state in an octahedral (O_h_) environment. To survive in the iron-deficient environment of their host, pathogenic bacteria have devised various efficient iron acquisition strategies. One such strategy involves the production of low molecular weight peptides called siderophores, which have a strong affinity and specificity to chelate Fe^3+^ and can thus facilitate uptake of this metal in order to ensure iron requirements. The Fe uptake by amphibactin and the release of iron inside the cell have been studied. Comparison of the interaction of different transition metal ions (M^3+^) with amphibactin has been studied and it reveals that Co and Ga form stable complexes with this siderophore. The competition of Co and Ga with Fe impedes iron uptake by bacteria, thereby preventing infection.

## Introduction

Metal ions are present in all microorganisms in many complexed forms, and they play key roles in numerous biological processes. Further studies of metal ion uptake by microorganisms are expected to shed more light on their life cycles and survival in different environmental conditions. The element Fe is essential for the growth of almost all microorganisms because it catalyzes several enzymatic processes, oxygen metabolism, electron transfer, and DNA and RNA syntheses, among others^1^. The *Vibrio* genus is curved-rod shaped gram-negative bacterium that is found in estuarine and marine environments worldwide. Several species of this genus can cause foodborne infection^2–9^. The seawater environments that are home to *Vibrionaceae* are slightly alkaline (pH 7.5–8.4), and the total concentration of soluble means Fe^+++^, FeOH^++^, Fe(OH)^2+^, Fe^++^, and FeOH^+^ in the absence of any chelating ligands is as low as 10^–10^ M, which is less than the amount required for bacterial growth (10^−6^–10^−7^ M)^10–12^. In biological systems, these Fe levels are further reduced (10^−15^–10^−25^ M) through sequestration by molecules such as transferrin and lactoferrin. For biological processes, Fe is required in a + 2 oxidation state. In aqueous-aerobic conditions and neutral p^H^, Fe is present mostly as insoluble Fe(III) hydroxide, which cannot be taken up by microorganisms^13,14^. In response to Fe scarcity and high biological demand, microorganisms have evolved strategies to acquire iron from the environment. In this regard, microorganisms secrete small-molecule peptides called siderophores, which chelate Fe^3+^, thereby ensuring their capacity to maintain biological processes^15–18^. Maintaining a dynamic equilibrium of Fe availability for the cell, i.e., Fe homeostasis is therefore critical for optimal cellular function, and indeed for life. Siderophores are metal-chelating agents with low molecular masses (200–2000 Da) produced by microorganisms and plants, especially under Fe-limiting conditions^2,19^. Several hundred siderophores, varying considerably in chemical composition, have been isolated and characterized. In this regard, they can be classified as hydroxycarboxylate-, catecholate-, or hydroxamate-type on the basis of the acidic moieties they hold in their metal-binding sites^2^.

The prime role of siderophores is to scavenge Fe. However, these small bioactive molecules also form complexes with other essential elements (i.e. Mo, Mn, Co and Ni), thereby mobilizing them and making them available for microorganisms^20–24^. The formation of Fe^3+^–siderophore complexes is affected by pH because of competition between free protons and Fe^3+^ ions for the free siderophore ligands^25^. In nature, Fe^3+^ competes for the siderophore binding sites not only with free protons but also with other metal ions, such as divalent cations Cd^2+^, Cu^2+^, N^i2+^, Pb^2+^ and Zn^2+^, trivalent cations Mn^3+^, Co^3+^ and Al^3+^, and actinides Th^4+^, U^4+^ and Pu^4+^^25–27^. Several studies have shown that siderophores influence the mobility of these metal ions in the environment^28^.

**Figure 1:**
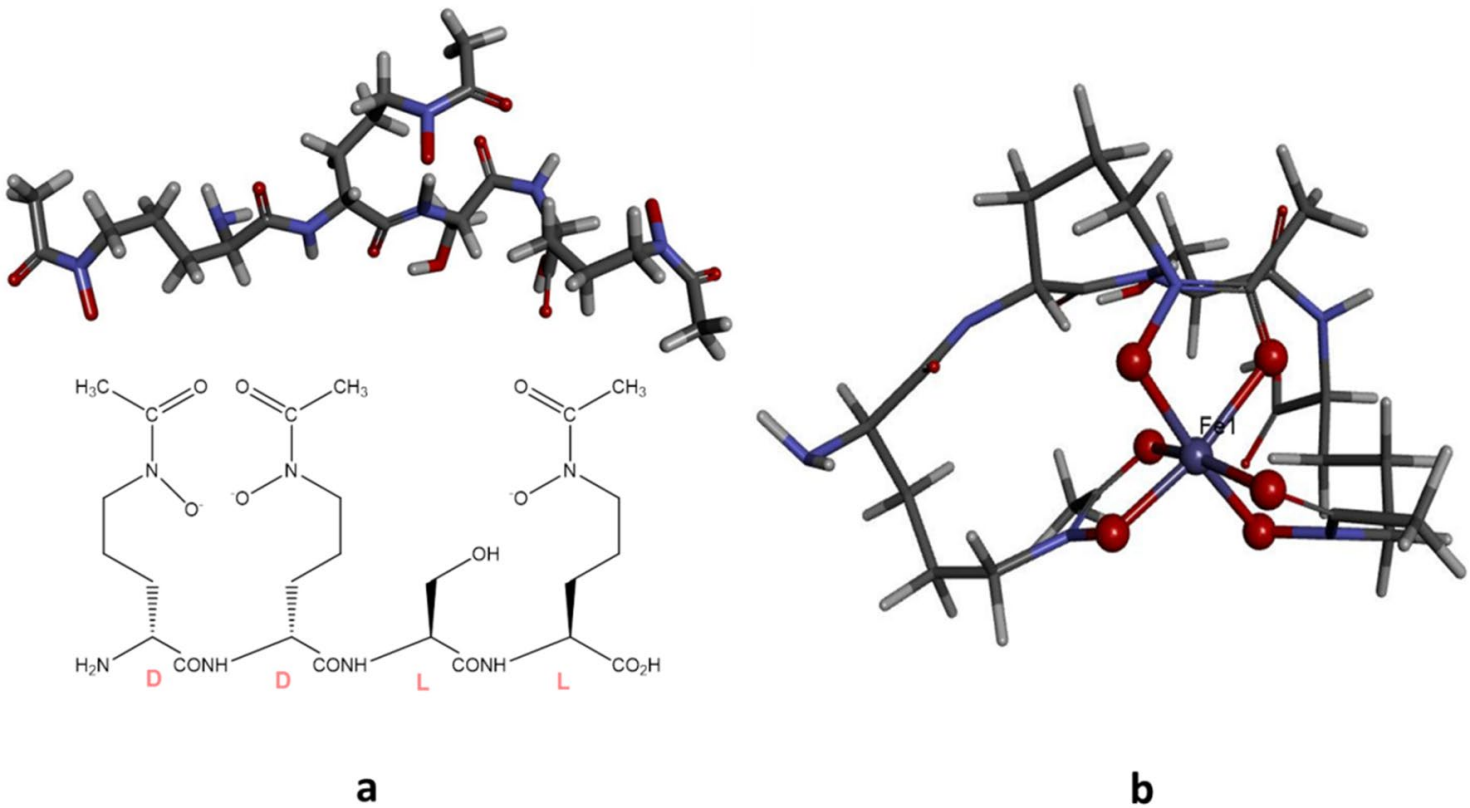
(**a**) Amphibactin, (**b**) amphibactin–Fe(III) complex. Color scheme: grey-carbon, red-oxygen, blue-nitrogen, purple/blue-iron, gray-hydrogen. All the structures are drawn using BIOVIA, Dassault Systèmes, Discovery Studio Visualizer, version: 17.2.0.16349, 2017. https://www.3ds.com/products-services/biovia/products/molecular-modeling-simulation/biovia-discovery-studio/visualization/ and PerkinElmer Informatics, ChemBioDraw UltraVersion: 14.0.0.117. https://cambridgesoft.com/Ensemble_for_Chemistry/details/Default.aspx?fid=14&pid=666.

In recent years, a novel family of cell-associated amphiphilic siderophores called amphibactin has been reported in marine gram-negative bacterium *Vibrio* sp. HC0601C5 (Fig. 1a). Containing a lipidic side chain attached to a polar head group, these siderophores are found only in waters with low Fe concentrations (< 0.3 nM)^29–38^. The amphibactin released from the bacteria specifically bind to Fe (Fe^3+^) from the environment, transport it into the cell, and then release it for cellular functions. Here we studied how amphibactin binds to Fe^3+^ and other + 3 oxidation state metal ions and whether this siderophore has greater affinity to bind metals other than Fe^3+^. Computational quantum chemistry tools can provide insights into the energetic parameters of metal complexation with amphibactin, the metal specificity of amphibactin, the metal–amphibactin interaction, the redox reaction, and the release of the Fe–amphibactin complex. Here we have computed different parameters to understand the binding of different M^3+^ ions with amphibactin and tried to deduce from these calculated parameters why certain M^3+^ ions form stable complexes with amphibactin. Fe is essential for many metabolic processes in microorganisms and developing an effective strategy to limit access to this metal will be a good defense against bacterial infections^15,39^. Several strategies to inhibit bacterial infections have been reported, and the research community faces the major challenge of antibiotic resistance of drugs^40–42^. In this context, the relative stability, interaction energy and affinity of the chelator amphibactin with various transition metal ions in + 3 oxidation state, other than Fe, are studied in order to examine the potential of using amphibactin–metal ion interaction as a tool to develop new drugs for antibiotic resistance. In this regard, here we explored the antibacterial activity of vibrio genus bacterium (*Vibrio* sp. HC0601C5) based on the chelation of metal ions Co^3+^ and Ga^3+^ which have similar ionic radii as Fe^3+^ with amphibactin. We demonstrate that the bacterial Fe uptake transport system can be exploited to deliver antimicrobials based on Co^3+^ and Ga^3+^ to the intracellular space of target bacteria with high specificity. This approach impedes Fe uptake and therefore blocks cellular processes.

## Results

### Molecular structure and stability of the complexes

Fe^3+^ prefers a hexa-coordinate octahedral ligand coordination sphere. The amphibactin–Fe(III) is a high spin d^5^ complex. Amphibactin has three hydroxamate ends; three of these groups bind Fe^3+^ in an O_h_ geometry, forming a neutral 1:1 complex with a single unpaired electron centered on the Fe^43^. Figure 1 shows optimized minimum energy structures for free amphibactin and amphibactin–Fe(III) complex. The optimized bond lengths and bond angles are given in Tables S1 and S2, and Fig. S1 (Supplementary information).

The geometric parameters of the optimized local minimum structures of amphibactin–metal ion complexes are given in Fig. S1 (SI). We performed a comparative study of the stability and affinity of amphibactin for Fe^3+^/Fe^2+^ and various transition metal ions in + 3 oxidation states by calculating and analyzing the parameters discussed below. The 1st row transition metals (Sc–Co), the Fe group (Ru, Os) and Mo, all of these with a preference for + 3 oxidation states and octahedral (O_h_) geometry, were examined.

#### Frontier molecular orbital analysis

The ground state reactivity was analyzed for amphibactin–transition metal ion complexes from the frontier molecular orbitals (FMOs): HOMO (highest occupied molecular orbitals) and LUMO (the lowest unoccupied molecular orbitals)^44^. The energy of the HOMO (*E*_HOMO_), energy of the LUMO (*E*_LUMO_) and the HOMO–LUMO gap (Δ*E*_*H–L*_) are used to describe the global reactivity and kinetic stability of a system^44–48^. The higher the value of ΔE_H–L_, the lower the reactivity of a molecule and vice versa. To evaluate the chemical reactivity or kinetic stability, the ΔE_H-L_ of all twelve amphibactin–transition metal ion complexes was calculated at B3LYP and M06/DEF2TZVP level (Table 1). The order of decreasing ΔE_H-L_ was as follows: Ga^3+^ > Sc^3+^ > Cr^3+^ > Mn^3+^ > Co^3+^ > V^3+^ > Fe^3+^ > Ti^3+^ > Mo^3+^ > Ru^3+^ > Fe^2+^ > Os^3+^ (B3LYP/def2tzvp level). At higher level calculation (M06/def2tzvp level) the order of decreasing ΔE_H-L_ gap was as follows: Ga^3+^ > Sc^3+^ > Cr^3+^ > Co^3+^ > Mn^3+^ > V^3+^ > Fe^3+^ > Mo^3+^ > Ti^3+^ > Ru^3+^ > Os^3+^ > Fe^2+^. The amphibactin–Ga^3+^ complex had the highest ΔE_H–L_, and hence was the most kinetically stable complex, the least stable being the amphibactin–Fe^2+^ and amphibactin–Os^3+^ complex from B3LYP/M06/def2tzvp calculations. Ionic radius of the metal ion is an important factor for amphibactin chelation with the metal ion. Here Sc^3+^ with high ΔE_H–L_ gap, but with large ionic radius compared to Fe^3+^ ion results in reduced preference for complexation with amphibactin (Table 2). The Sc^3+^ in *d*^0^ O_h_ geometry with high ΔE_H-L_ gap showed low E_int_, MIA, δ and ∆G (Tables 2 and 3). The Cr^3+^, Mn^3+^, Co^3+^ and V^3+^ have higher ΔE_H–L_ than Fe^3+^. The high ΔE_H–L_ for Cr^3+^ may be due to high LFSE (Ligand Field Splitting Energy) value (~ − 1.2Δ_0_, high spin *t*_*2g*_^3^) in an O_h_ geometry. The high spin *d*^*2*^ V^3+^ also showed high ΔE_H–L_ than Fe^3+^ due to high LFSE it had in an O_h_ environment. The Mn^3+^ shows a higher ΔE_H–L_ gap, but the *d*^*4*^ high spin *t*_*2*_*g*^3^* eg*^*1*^ Mn^3+^ ion was unstable and readily decomposes to Mn^2+^ and Mn^4+^ in aqueous condition^49^. The Co^3+^ had large ΔE_H-L_ gap value as it forms stable O_h_ complex with amphibactin. The optimized structure of the amphibactin–Mo complex was broken with the loss of metal–ligand six-coordinate O_h_ geometry. The results from Table 1 confirmed that out of the metal ions with similar ionic radii as Fe^3+^, amphibactin–Fe^3+^/Ga^3+^/Co^3+^/Cr^3+^/Mn^3+^ complexes were the most kinetically stable or were less reactive than other complexes.

**Table 1:** HOMO–LUMO gap ΔE_H–L_ and chemical hardness $\eta$ calculated in eV for different amphibactin–metal complexes in O_h_ geometry.

S. no.	Complex	ΔE_H–L_		$\eta$	
		B3LYP	M06	B3LYP	M06
1	Amphi–Fe^3+^	3.28	3.41	1.64	1.71
2	Amphi–Fe^2+^	2.57	2.78	1.29	1.39
3	Amphi–Cr^3+^	4.43	4.58	2.22	2.29
4	Amphi–Ru^3+^	2.61	2.96	1.31	1.48
5	Amphi–Os^3+^	2.52	2.80	1.26	1.40
6	Amphi–Mo^3+^	3.15	3.37	1.58	1.69
7	Amphi–Ga^3+^	5.04	5.71	2.52	2.86
8	Amphi–Mn^3+^	4.04	3.83	2.02	1.92
9	Amphi–Sc^3+^	4.98	5.64	2.49	2.82
10	Amphi–Ti^3+^	3.33	3.36	1.67	1.68
11	Amphi–V^3+^	3.46	3.51	1.73	1.76
12	Amphi–Co^3+^	3.78	4.29	1.89	2.15

**Table 2:** Ionic radii of metal ions in pm, interaction energy *E*_*int*_ with ZPE + BSSE correction, metal ion affinity (MIA) and deformation energy δ of amphibactin–metal complexes in kcal/mol calculated at B3LYP/M06/DEF2TZVP level.

Metal ion	Ionic radii (pm)^a^	(*E*_*int*_) with BSSE		MIA		δ	
		B3LYP	M06	B3LYP	M06	B3LYP	M06
Fe^3+^	65	− 1478.64	− 1498.61	1475.05	1496.46	216.86	198.64
Fe^2+^	78	− 757.08	− 777.90	754.18	777.69	34.54	169.87
Cr^3+^	62	1384.67	− 1439.14	1414.27	1437.92	222.29	203.67
Ru^3+^	68	− 1449.36	− 1465.06	1452.16	1470.52	215.13	196.72
Os^3+^	77	− 1446.97	− 1445.93	1448.96	1464.31	215.15	196.25
Mo^3+^	69	− 1413.82	− 1376.07	1418.65	1426.83	321.57	321.57
Ga^3+^	62	− 1442.04	− 1452.99	1447.06	1457.50	218.57	199.82
Mn^3+^	65	− 1477.38	− 1503.39	1476.78	1501.03	227.99	208.58
Sc^3+^	75	− 1276.84	− 1282.73	1282.62	1288.26	206.82	188.13
Ti^3+^	67	− 1342.20	− 1356.11	1348.14	1360.82	214.27	199.46
V^3+^	64	− 1381.24	− 1405.37	1378.17	1403.53	217.82	201.00
Co^3+^	61	− 1567.48	− 1588.34	1572.84	1593.10	237.29	218.52

^a^Shannon^50^, Ahrens^51^ and Choi et al.^52^.

**Table 3:** Free energy change ∆G, enthalpy change ∆H, entropy change ∆S, translational entropy change ∆S_*trans*_, rotational entropy change ∆S_*rot*_ and vibrational entropy change ∆S_*vib*_ calculated in kcal/mol for the different amphibactin–metal complexes at B3LYP/M06/DEF2TZVP level.

Metals	∆G		∆S		∆S_*trans*_		∆S_*rot*_		∆S_*vib*_	
	B3LYP	M06	B3LYP	M06	B3LYP	M06	B3LYP	M06	B3LYP	M06
Ga^3+^	− 1427.31	− 1440.37	− 71.19	− 62.42	− 38.29	− 38.29	− 1.83	− 1.68	− 31.06	− 22.45
Co^3+^	− 1551.22	− 1574.45	− 77.47	− 67.51	− 37.87	− 37.87	− 1.92	− 1.76	− 37.68	− 27.89
Cr^3+^	− 1402.96	− 1427.45	− 72.40	− 62.93	− 37.52	− 37.52	− 1.86	− 1.70	− 33.0	− 23.70
Fe^2+^	− 743.92	− 766.73	− 60.86	− 55.12	− 37.73	− 37.73	− 1.73	− 1.66	− 21.40	− 15.73
Fe^3+^	− 1464.17	− 1486.39	− 68.15	− 57.81	− 37.3	− 37.73	− 1.87	− 1.72	− 28.55	− 18.36
Mn^3+^	− 1465.45	− 1490.37	− 72.87	− 63.14	− 37.68	− 37.68	− 1.87	− 1.73	− 33.32	− 23.74
Mo^3+^	− 1401.35	− 1410.80	− 62.98	− 58.76	− 39.22	− 39.22	− 1.54	− 1.44	− 22.22	− 18.10
Os^3+^	− 1428.53	− 1446.91	− 73.50	− 63.34	− 40.83	− 40.83	− 1.69	− 1.53	− 30.98	− 20.97
Ru^3+^	− 1431.75	− 1452.97	− 73.43	− 63.82	− 39.32	− 39.32	− 1.77	− 1.61	− 32.34	− 22.89
Sc^3+^	− 1265.18	− 1270.58	− 63.47	− 64.24	− 37.13	− 37.13	− 1.73	− 1.61	− 24.62	− 25.50
Ti^3+^	− 1329.99	− 1344.59	− 65.82	− 59.40	− 37.30	− 37.30	− 1.76	− 1.61	− 26.76	− 20.48
V^3+^	− 1367.50	− 1393.07	− 68.73	− 62.03	− 37.47	− 37.47	− 1.78	− 1.64	− 29.47	− 22.91

#### Hardness

Chemical hardness (η), first put forward by Pearson, is a measure of the resistance of a chemical species to change its electronic configuration^53,54^. According to the Hard and Soft Acid and Bases (HSAB) principle, a hard acid prefers a hard base and a soft acid prefers a soft base. Here, free Fe^3+^ has an ionic radius of 67 pm and acts as a “hard” acid, preferring “hard” oxygen ligands such as phenolate (from Tyr) and carboxylate (from Asp or Glu). Free Fe^2+^ has an ionic radius of 83 pm and is borderline between a “hard” and “soft” acid, favoring coordination of nitrogen atoms (from His or pyrrole) and sulfur ligands (from Cys or Met) over oxygen ligands^55^. Since the parameter η indicates a preference of a hard/soft acid for a hard/soft base, for our purposes it provides a quantitative value of the preference of the chelator amphibactin for distinct metal ions.

1 $$\eta ={\frac{{(E}_{LUMO}-{E}_{HOMO})}{2}}$$

The $\eta$ values were calculated for all the amphibactin–metal complexes (Table 1). Since the ionic radius was an important factor deciding the complexation of amphibactin with metal ion, the Sc^3+^ with large ionic radius (Table 2) can be excluded even though it had large $\eta$ value. The Fe^3+^, Ga^3+^, Co^3+^, Cr^3+^ and Mn^3+^ have similar ionic radii (Table 2).The $\eta$ values were higher for amphibactin complexes with Fe^3+^, Ga^3+^, Cr^3+^, Mn^3+^ and Co^3+^, with the amphibactin–Ga complex showing the highest $\eta$ value. The high $\eta$ value for the amphibactin–Ga complex indicates that it was chemically the least reactive after complexation and hence it was kinetically stable or inert.

#### Interaction energy

Interaction energies (*E*_*int*_) between amphibactin and the different transition metal ions were calculated using the following equation

2 $${E}_{int}={E}_{metal ion{\text {--}}amphibactin}-{E}_{amphibactin}-{E}_{ion}$$

where *E*_*ion–amphibactin*_ is the energy of the ion–amphibactin complex, *E*_*amphibactin*_ is the energy of the isolated amphibactin, and *E*_*ion*_ is the energy of the isolated metal ion. The larger the negative interaction energy, the stronger the intermolecular interaction between amphibactin and the transition metal ion. The *E*_*int*_ with ZPE and BSSE correction for all the amphibactin–metal complexes are reported in Table 2. An important factor for amphibactin metal complexation is the size of the metal ion as the amphibactin was produced especially to chelate Fe^3+^ ions (~ 64 pm). The Fe^3+^, Co^3+^, Ga^3+^, and Cr^3+^ have similar ionic radii around (62–64 pm) and the ionic radii of Mn^3+^ and V^3+^ were next closer to Fe^3+^. Among these six metal ions the order of E_int_ was Co^3+^ > Fe^3+^ ~ Mn^3+^ > Ga^3+^ > Cr^3+^ > V^3+^.The amphibactin–Fe^3+^/Co^3+^/Ga^3+^/Mn^3+^ complexes showed large negative *E*_*int*_ values, with Co^3+^ having maximum interaction energy (Table 2). Amphibactin showed a high preference to complex with Fe^3+^, Ga^3+^, Mn^3+^ and Co^3+^, and the Co^3+^ ion interacted with the chelator strongly to form the most stable complex. The amphibactin–Fe^2+^ complex showed the lowest *E*_*int*_, making it the least stable complex and also the resulting optimized structure did not retain O_h_ geometry at B3LYP/DEF2TZVP level. At higher level calculation M06/DEF2TZVP, the amphibactin–Fe^2+^ complex showed a distorted O_h_ geometry with a large metal–ligand distance (5, Fig. S1, Table S1). It should be noted that amphibactin bound strongly to Fe^3+^, Co^3+^, Mn^3+^ and Ga^3+^, and all ions have almost the same ionic radius (Table 2). This observation reveals that amphibactin is specific and forms stable complexes with metal ions with this particular ionic radius. The amphibactin–Mn^3+^ complex was also found to be considerably stable, as reflected by the *E*_*int*_ value. The high spin *d*^*4*^ Mn^3+^ (~ 66–67 pm) preferred a tetragonal coordination compared to O_h_ due to Jahn–Teller effect (evident from bite angle and bond distances Tables S1 and S2). The Mn^3+^ ions shows a high tendency to disproportionate to Mn^2+^ or Mn^4+^ in neutral and acidic condition in water and was less stable^56–58^. The Cr^3+^ with an ionic radius of around 62–63 pm has *E*_*int*_ value lower than that of Fe^3+^, Ga^3+^ and Co^3+^ due to decreased metal–ligand interaction as the effective nuclear charge or penetration effect increases from left to right along the 1st row transition metals. The higher values for E_int_ for Ru^3+^ and Os^3+^ may be due to the high penetration effect or effective nuclear charge and hence stronger metal–ligand interactions. As we go down the Fe group, the penetration effect increases due to more diffuse orbitals and hence it was reflected in the higher values for E_int_, MIA and ∆G. The remaining metal ions (Mo^3+^, Sc^3+^, Ti^3+^, V^3+^) showed lower *E*_*int*_ values and larger ionic radii.

#### Relaxation/deformation energy

Relaxation or deformation energies (*E*_*relax*_) are derived by subtracting the complexation energies (unrelaxed single point energy, amphibactin without metal ion) from the interaction energy value (relaxed, optimized amphibactin). The *E*_*relax*_ values for the amphibactin–metal complexes are given in Table 2 and they provide a measure of how a metal ion can induce a specific conformation (O_h_ geometry) from the preferred conformation of amphibactin

3 $${E}_{relax}={{E}_{amphibactin\;complex\;without\;metal\;(unrelaxed)}-E}_{metal\;free\;amphibactin\;(relaxed)}$$

The large the value of *E*_*relax*_, indicates greater the capacity of metal ion to coordinate with amphibactin in six-coordinate O_h_ conformation and form a stable complex. The Fe^3+^, Co^3+^, Ga^3+^, and Cr^3+^ have similar ionic radii around (62–64 pm). The ionic radii of Mn^3+^ and V^3+^ were next closer to Fe^3+^. The order of relaxation energy (δ) is Co^3+^ > Mn^3+^ > Cr^3+^ > V^3+^  > Fe^3+^ > Ga^3+^ at M06/def2tzvp level. The metal ions Ru^3+^, Os^3+^, Sc^3+^, except (Mn^3+^, Ti^3+^ and V^3+^) had low values of *E*_*relax*_. The inducing effects of the ions on the conformation of amphibactin also depended on the strength of the amphibactin–metal ion interactions. This observation may explain why Cr^3+^, Mn^3+^ and V^3+^ showed slightly higher. The high spin *d*^*4*^ Mn^3+^ (~ 66–67 pm) with a stable tetragonal geometry compared to O_h_ due to Jahn–Teller effect causes two of the bond move apart and other four Mn-ligand bonds to come closer (Table S1). This more inducing effect of Mn^3+^ on amphibactin conformation causes high *E*_*relax*_ and closer metal–ligand interaction. The high spin *d*^3^ Cr^3+^ with *t*_*2g*_^3^ levels has high preference for an O_h_ structure with a high LFSE gap resulting in a larger *E*_*relax*_ value. The *d*^10^ Ga^3+^ with a LFSE = 0 has equal preference for both an O_h_ and T_d_ geometry. With large ligands, to avoid ligand–ligand repulsion and metal ions with zero LFSE (*d*^0^, *d*^5^ and *d*^10^), the tetrahedral geometry is preferred compared to O_h_. For a metal ion to induce an O_h_ geometry causes more *E*_*relax*_ and hence this may be the reason for higher *E*_*relax*_ value for Cr^3+^ than Ga^3+^. The high spin *d*^2^ V^3+^ also has higher preference for O_h_ geometry than Ga^3+^ and therefore a higher E_relax_ value. Therefore from above the discussion, Co^3+^ metal ion had the largest *E*_*relax*_ values, thereby making it the most stable O_h_ complexes, Fe^2+^ being the least stable with lowest *E*_*relax*_. The *E*_*relax*_ values decreased with the increase in the atomic radii of the ions upon complexation with amphibactin. The Mo^3+^ does not retain an O_h_ geometry after optimization (one of the bonds is broken) and the high δ value accounts for that broken bond.

#### Metal ion affinity

Metal ion affinity (MIA) is one of the methods to study the stability of metal–ligand interactions and was calculated as,

4 $$MIA=-\left[{E}_{el}\left(ion{\text{--}}amphibactin\right)-{E}_{el}\left(amphibactin\right)-{E}_{el}\left(ion\right)+\left({E}_{vib}\left(ion{\text{--}}amphibactin\right)-{E}_{vib}\left(amphibactin\right)\right)\right]$$

where *E*_*el*_ is the electronic energy obtained from the SCF (Self Consistent Field) computation and *E*_*vib*_ include the Zero point energy and temperature corrections from 0 to 298 K obtained by the thermo-chemical analysis of vibrational frequencies^59–61^. MIA is considered as the negative of the enthalpy for the dissociation of the amphibactin–metal complex to free amphibactin and metal ion. The higher the MIA value, the greater the affinity of the metal ion for amphibactin. MIA values of the amphibactin–metal complexes showed a similar trend to the *E*_*int*_ values (Table 2). Among these six (Co^3+^, Fe^3+^, Mn^3+^, Ga^3+^, Cr^3+^, V^3+^) metal ions with almost same ionic radii, the order of MIA are: Co^3+^ > Fe^3+^ ~ Mn^3+^ > Ga^3+^ > Cr^3+^ > V^3+^. The Mn^3+^ with its *d*^4^ high spin tetragonal structure promotes better metal–ligand interaction and hence the large MIA value. The decreased metal–ligand interaction in case of Cr^3+^ and V^3+^ results in low MIA. The higher values for MIA for Ru^3+^ and Os^3+^ may be due to high (LFSE) expected in these complexes due to more interaction between the ligand and diffuse valence metal orbitals. These distorted O_h_ structures were less stable which is evident from their relaxation energies (Table 2 and Table S2). Out of the metal ions with similar ionic radii as Fe^3+^, MIA values for Fe^3+^, Ga^3+^ and Co^3+^ were larger than the other metal ions examined, Co^3+^ having the highest value. This observation further confirms the affinity of amphibactin for Co^3+^ followed by Fe^3+^ and Ga^3+^ and compared to other transition metals in the + 3 oxidation state.

#### Chelate angle and interatomic distances

**Figure 2:**
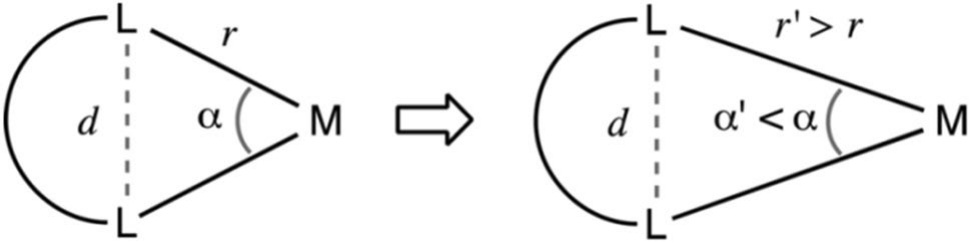
Chelate angle α =  < L–M–L, where *L* ligand, *M* metal.

The rigidity of the chelate ligand is measured by the bite angle (α) of the chelate ring and is sensitive to the metal–ligand distance. The bite angle α is defined as the L–M–L angle (Fig. 2). The longer the bond distance, the smaller the α. Octahedral and square planar complexes show a preference for a α of around 90°, while for tetrahedral complexes it is 110°. If the Ls are too far apart, thus resulting in a much bigger α, one end of the ligand will dissociate, indicative of low stability of the complex. For chelate rings with a α of less than 90°, the coordination sphere extends from O_h_ toward the trigonal prism with smaller α. As shown in Table S2, the α for Fe^3+^, Ga^3+^, Mn^3+^, Cr^3+^ and Co^3+^ were close to 90°, as required for an O_h_ coordination sphere, thus making these metal ions more stable than others with a smaller bite angle. The decreasing order of α: Co^3+^ > Mn^3+^ > Ga^3+^ ~ Cr^3+^ > Fe^3+^. The bite angles for remaining metal ions were farther away from 90°, thus indicating a more deviation from O_h_ geometry. If ligands were large; so as to avoid ligand-ligand repulsion, for metal ions (Sc^3+^, Fe^3+^, Ga^3+^, Ru^3+^, Os^3+^) with zero LFSE (*d*^0^, *d*^5^ and *d*^10^), the tetrahedral geometry was preferred over O_h_. These metal ions have showed deviation from O_h_ geometry (Table S2). The Co^3+^ shows maximum value of 82.9/83.6° making amphibactin–Co^3+^ the most stable complex. In the case of the amphibactin–Mo complex, it had the largest deviation from α of 90° with most deviation from O_h_ geometry.

#### Thermodynamic properties

To further understand the stability of amphibactin–metal ion complexes and binding preference of amphibactin for a particular metal ion, the enthalpy, free energy, entropy and individual contributions (translational, rotational, and vibrational) of the transition metal ions complexed with amphibactin are reported in Table 3. The ∆G, ∆H, ∆S parameters explain the thermodynamic stability of the complex. A negative ∆G/∆H indicates the formation of a stable complex. Chelation or complex formation causes ∆S to be negative. As a result of negative S values, G values are less negative than their corresponding H values. The ∆H values recorded for the complexes were slightly less negative than their ∆G values (Table 3). Examination of the thermodynamic parameters revealed Fe^3+^ and Co^3+^ as the metal ions with the largest ∆G/∆H values, thereby allowing them to form strong complexes with amphibactin. In contrast, Fe^2+^ was the least stable metal ion, as reflected by the lowest ∆G/∆H values. The ∆G/∆H values depended on the value of ∆S. Large negative ∆S values were found for Ga^3+^, Co^3+^, Fe^3+^, Mn^3+^, Os^3+^ and Ru^3+^. We calculated the individual contributions to total ∆S. The ∆S_*trans*_ and ∆S_*rot*_ became more negative as the size of the complex grew and is purely size-dependent. This explains the reason for large negative ∆S_*trans*_ values for Os^3+^ and Ru^3+^ complexes because as we go down the periodic table the size increases due to more diffuse orbitals. The ∆S_*rot*_ depends on the symmetry and geometry of the complex formed. If the resulting complex has highly symmetric, like O_h_ geometry the ∆S_*rot*_ will be more negative. From Table 3 it was clear that Co^3+^, Fe^3+^, Ga^3+^, Cr^3+^ and Mn^3+^ had larger negative ∆S_*rot*_ values implying that the complexes formed by these metal ions have more symmetric O_h_ geometry and contributes to its stability. Also the bite angles calculated for above mentioned metal ions were closer to O_h_ geometry. The ∆S_*vib*_ depended on the strength of the interaction between the metal ion and amphibactin. As the complex became stronger, the vibrations became stiffer, and the ∆S_*vib*_ became more negative. In case of Ga^3+^, Co^3+^, Fe^3+^, Cr^3+^, Mn^3+^, the ∆S_*vib*_ values were high, indicating strong amphibactin–metal interactions in these complexes. The ions Cr^3+^, Sc^3+^, Ti^3+^, Os^3+^ and Ru^3+^ had high ∆S_*vib*_ but with low ∆G values. The high ∆S_*vib*_ value for Cr^3+^ may be due to the high LFSE value exhibited by Cr^3+^ in O_h_ coordination, but the weak metal–ligand interaction results in low ∆G value. In the case of Sc^3+^ and Ti^3+^ as well, the high LFSE in O_h_ environment contribute to high ∆S_*vib*_ values. The higher penetration effect in Os^3+^ and Ru^3+^ assist in better metal–ligand interaction and hence higher ∆S_*vib*_ values for these ions. The Mn^3+^ also formed a stable complex with amphibactin. However, Mn^3+^ complexes are generally unstable in aqueous neutral and acidic pH environments and they decompose in water to produce Mn^2+^ and Mn^4+^^56–58^. Also Mn^3+^ prefers a tetragonal geometry in d^4^ state^62^. In tetragonal geometry either two ligands move apart or come closer to the central metal ion to reduce the degeneracy. In amphibactin–Mn(III) complex two of the bond distances showed considerable difference compared to other four Mn–ligand bonds (Table S1). The thermochemical analysis also reveals amphibactin–Co^3+^ forms the stable complex compared to other amphibactin–metal ions studied.

### Redox reaction

A powerful strategy to fight microbial infections is to prevent pathogens from acquiring Fe. The reduction of Fe^3+^ to Fe^2+^ is an important step in the Fe uptake process. A greater understanding of this step may contribute to the development of molecules that inhibit Fe uptake. All the following calculations were carried out at B3LYP/6-31g(d,p)/LANL2DZ level in gaseous and aqueous condition (dielectric constant ε = 78.4) in G09 CPCM model.

$$Fe^{3 + } \;to\;Fe^{2 + } \;reduction{:}\quad {\text{Fe}}\left( {{\text{III}}} \right)\;{\text{amphibactin}} + \overline{{\text{e}}} \to {\text{Fe}}\left( {{\text{II}}} \right)\;{\text{amphibactin}}$$

The molecular weights of the ferric–siderophore complexes exceeded the cut off for porins and therefore require specific outer membrane receptors for their uptake into the cell through the periplasmic space. Once siderophores enter the cytoplasm, the Fe from the ferric-siderophore complex can be released as Fe^2+^ for metabolic processes in the following two ways^2,15,63^.

**Figure 3:**
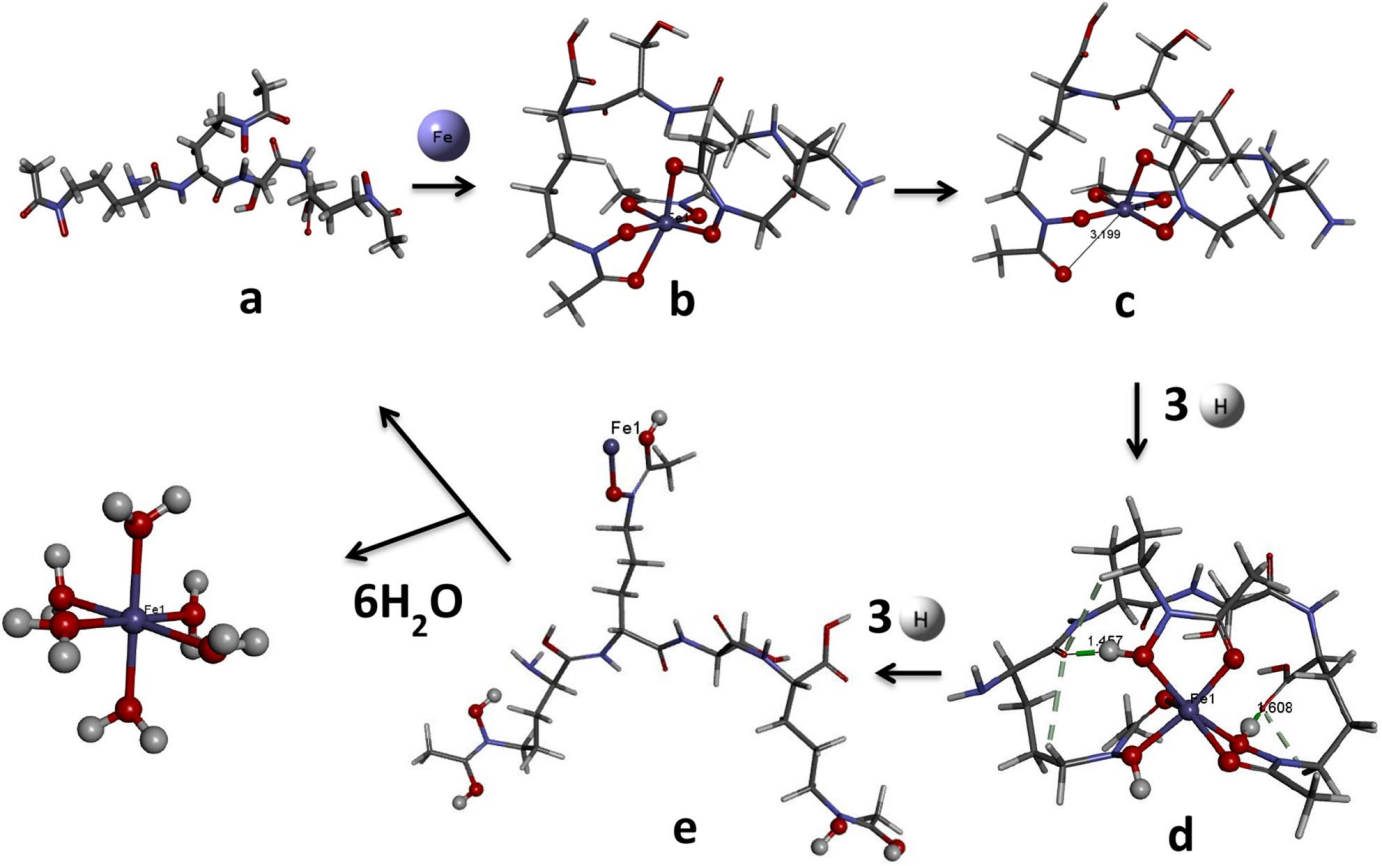
Mechanistic pathway showing Fe(III) acquisition followed by reduction to Fe(II) and further protonation steps to release Fe(II). Optimized minimum energy structures of (**a**) free amphibactin (**b**) amphibactin–Fe(III) complex (**c**) amphibactin–Fe(II) complex (**d**) three-hydrogen protonated amphibactin–Fe(II) complex (**e**) six-hydrogen protonated amphibactin–Fe(II) complex, respectively, at B3LYP/6-31g(d,p)/LANL2DZ level. All the structures are drawn using BIOVIA, Dassault Systèmes, Discovery Studio Visualizer, version: 17.2.0.16349, 2017. https://www.3ds.com/products-services/biovia/products/molecular-modeling-simulation/biovia-discovery-studio/visualization/.
  Further protonation of the reduced amphibactin–Fe^2+^ complex by three hydrogen atoms in aqueous medium was calculated using Scheme 1 (see below) and the free energy change ${(\Delta {\varvec{G}}}_{{\varvec{t}}}^{\#})$ was found to be negative, typical of a spontaneous process. The CPCM model in Gaussian 09 was used to calculate the free energy for protonation from the optimized amphibactin–Fe^2+^ complex with three hydrogen atoms added to the (N–O)hydroxamate group coordinated Fe^2+^ metal ion in aqueous medium.

1. The reduction of amphibactin–Fe(III) to amphibactin–Fe(II) complex, followed by protonation and hydrolysis to release Fe^2+^ in to the cell cytoplasm (see Fig. 3). The first step is the reduction of amphibactin–Fe^3+^ complex that has entered the cytoplasm. The free energy change for the reduction of amphibactin–Fe(III) to amphibactin–Fe(II) complex with zero point correction in aqueous solvent $({\Delta G}_{s}^{0})$ was negative ($-112.35$ (kcal/mol)). The Eq. (S1) (Supplementary) gives the standard reduction potential, ${E}^{0}\left(V|RE\right)=0.44 V$, for the reduction reaction of amphibactin–Fe^3+^ to amphibactin–Fe^2+^. This was compared with standard hydrogen electrode (SHE) using Eqs. (S2) and (S3) and was positive, thereby indicating that the complex reduces readily in an acidic p^H^^42,64^. Bacterial efflux pumps (EPs) are proteins that are localized and imbedded in the plasma membrane of the Gram-negative bacterium are responsible for the pH dependent binding and release of the substrate and for other reactions like reduction^65^. Many vibrio species use NADH with ferric reductase enzyme^66–72^. Inside the cell, NADH acts as a potential electron donor for the enzyme. Therefore we can couple the reduction reaction of amphibactin–Fe^3+^ to amphibactin–Fe^2+^ with NADH oxidation reaction. The overall redox reaction is the sum of the two half-cell reactions or couples: (a) reduction of amphibactin–Fe^3+^ to amphibactin–Fe^2+^ and (b) oxidation of NADH → NAD^−^ + H^+^. We calculated the above mentioned two half-cell reactions. ${E}_{Cell}^{0}$ was found to be positive (0.12 V), thereby establishing that the reaction slightly spontaneous, see Eq. (S4). A negative value of ${E}_{Cell}^{0}$ indicates that the reaction will proceed spontaneously in the opposite direction. The E^h^_cell_ at p^H^ = 7 was calculated from the Nernest equations (S8–S10). The E^h^_cell_ calculated was 0.12 V at p^H^ = 7 (S16). The E°_cell_ and E^h^_cell_ calculated at p^H^ = 0 and p^H^ = 7 respectively for the reduction of amphibactin–Fe(III) by NADH was the same 0.12 V (Eqs. S4 and S16). Even though with small E_cell_ value, the reduction was facilitated at p^H^ = 7. The standard electrode potentials calculated for the reduction amphibactin–Fe(III) to amphibactin–Fe(II) at p^H^ = 0, E^0^ = 0.44 and at p^H^ = 7, E_h_ = 0.02588 respectively are different and E_h_ < E^0^ (Eqs. S2 and S10). The E_h_ for Fe^3+^ → Fe^2+^ at p^H^ = 2 is 0.32 V (Eq. S12) indicating the reduction would be more spontaneous at an acidic p^H^. The ∆G°_h_ at p^H^ = 7 was calculated from the E_h_ value of Fe^3+^ → Fe^2+^ reduction potential (Eqs. S22–23). The ∆G°_h_ = − 0.60 kcal/mol (Eq. S11) is slightly non-spontaneous at p^H^ = 7 and ∆G°_h_ = − 7.42 kcal/mol (Eq. S13) at p^H^ = 2, indicating that the complex will reduce readily at an acidic p^H^. It is well known that the reduction Fe^3+^ to Fe^2+^ happens inside the cell where proton gradient generated to lower the extracellular pH to acidic p^H^. Using a milder reducing agent than NADH like FADH_2_ in the cell may increase the E_cell_ value. Usually, the steps which were coupled with NADH reduction had a free energy change of about Δ*G* =  − 100 to − 150 kcal/mol. The reduction of amphibactin–Fe(III) to amphibactin–Fe(II) has a free energy value of 110 kcal/mol. Therefore it might not be feasible to couple with NADH reduction but it would be favourable (thermodynamically) to couple with an easy-to-reduce and less energetic molecule FADH_2_. With FADH_2_ (free) as reducing agent, the E_cell_ = 0.22 V (Eq. S19) and FADH_2_ (bound) E_cell_ = 0.41 V (Eq. S21) at a p^H^ = 7 indicating the reduction of amphibactin–Fe(III) to amphibactin–Fe(II) is spontaneous and favoured.

**Scheme 1:**
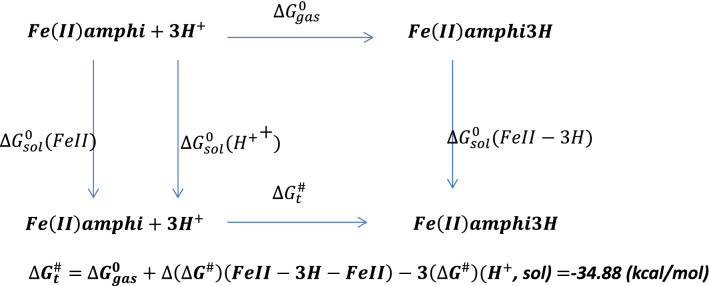
Protonation of amphibactin–Fe^2+^.
2. The protonation of amphibactin–Fe^3+^ followed by reduction reaction. The free energy change for the protonation reaction of amphibactin–Fe^3+^ with three hydrogen atoms attached to the (N–O) hydroxamate group in aqueous medium was calculated using Scheme 2 (see below) and was found to be positive and thus a non-spontaneous reaction. Comparing the free energy changes obtained using Schemes 1 and 2, the former was more thermodynamically favorable than the latter. The former was a spontaneous process with a negative ∆G value whereas the latter was non-spontaneous with a positive ∆G.
  At physiological p^H^ = 7, the ∆G for Schemes 1 and 2 are − 6.26 kcal/mol and 39.07 kcal/mol respectively (Eq. S23). This implies that the Scheme 1 is spontaneous process and Scheme 2 non-spontaneous.

**Scheme 2:**
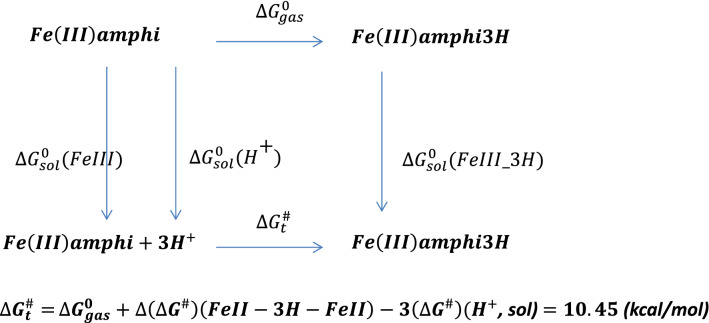
Protonation of amphibactin–Fe^3+^.

#### Protonation reaction

The Δ*E*_*H–L*_, chemical hardness (η), E_*int*_ and relaxation energy were calculated for amphibactin–Fe^3+^, amphibactin–Fe^2+^, 3H protonated amphibactin–Fe^2+^ and 6H protonated amphibactin–Fe^2+^ respectively (Table 4). The protonation of reduced amphibactin–Fe complex was done first by the addition of three hydrogen atoms to the three oxygen atoms of N–O (ligand) of amphibactin, followed by the addition of a further three hydrogen atoms to the three oxygen atoms of C=O chelating with Fe. Hydroxamates show p^Ka^ values from 8 to 9 in seawater environments that are home to *Vibrionaceae* with slightly alkaline (pH 7.5–8.4)^73–75^. The energy for protonation of O of CO 7.44 kcal/mol higher than protonation of O of NO of hydroxamate group implying NO protonation was more preferred compared to CO protonation. Here a proton (H^+^) was attached to one of the O of CO of hydroxamate group of the three hydrogen protonated at three O atoms of NO of hydroxamate group amphibactin–Fe(II)–3H complex to get amphibactin–Fe(II)–4H complex. Computationally from G09 the p^Ka^ for hydroxamate CO calculated from the Eq. (S26) was around 8.15. As we go from **1** to **4** the stability of the complex decrease from the E_*int*_ decreases and relaxation energy increases while its reactivity increases from the decrease in the Δ*E*_*H–L*_ and η (Table 4). The higher Δ*E*_*H–L*_ for **2** was due to additional hydrogen bonding interactions in the system due to conformational/geometry change on protonation (d, Fig. 3). This indicates that the reduction of Fe^3+^ to Fe^2+^ in the Fe–amphibactin complex, followed by 3H and 6H pronation reduced the thermodynamic stability and reactivity of the complexes. Fe^3+^ is a hard metal ion that has a preference for hard ligands, like oxygen. Fe^2+^ is a borderline metal ion that prefers tetrahedral coordination and softer/borderline ligands such as nitrogen (e.g., porphyrin, PhNH_2_) or sulphur (RSH, R_2_S). Therefore, the reduction of amphibactin–Fe^3+^ (**1**) to amphibactin–Fe^2+^ (**2**) drastically lower the affinity of the siderophore for the Fe^2+^ ion. The E_*int*_ also decreases down Table 4, with positive interaction energy for **4**. This implies that Fe^3+^ (**1**) reduction to Fe^2+^ (**2**) followed by subsequent protonation (**3** and **4**) reduce the affinity of siderophore for Fe^2+^ ion, thereby releasing the Fe into the cell. The Fe-free siderophore is usually degraded or recycled by excretion through an efflux pump system (like H^+^/Na^+^ driven pump). Further hydrolysis of amphibactin–Fe^2+^–3H is a spontaneous process with a ΔG = − 517.16 kcal/mol.

$${\text{Amphi}}{\text {--}}{\text{Fe}}^{{{2} + }} {-}{\text{3H}} + {{\text{6H}}_{{2}}} {\text{O}} \to {\text{Fe}}\left( {{{\text{H}}_{{2}}} {\text{O}}} \right)_{{6}}^{{{2} + }} + {\text{Amphibactin}}$$

**Table 4:** The Δ*E*_*H–L*_, chemical hardness (η), interaction energy and relaxation energy δ calculated for amphibactin–iron complex in + 2 and + 3 oxidation states, and amphibactin–Fe^2+^ with three and six hydrogen protonated complexes respectively at B3LYP/6-31 g(d,p)/LANL2DZ level.

S. no.	Complex	HOMO–LUMO gap Δ*E*_*H–L*_	Chemical hardness (η)	Interaction energy	Relaxation energy δ
1	Amphi–Fe^3+^	3.15	1.58	− 1514.38	216.65
2	Amphi–Fe^2+^	2.90	1.45	− 773.60	174.68
3	Amphi–Fe^2+^–3H	3.86	1.93	− 387.14	77.61
4	Amphi–Fe^2+^–6H	0.30	0.15	− 27.05	55.47

The resulting aqua complex of Fe^2+^(Fe(H_2_O)_6_^2+^) was stable, with a Δ*E*_*H–L*_ value of 5.77 eV, and can therefore be further used for metabolic processes inside the cell.

### Co (III) and Ga (III) complexes as antibacterial agents

An alternative approach to block Fe uptake to limit pathogen growth is to use the bacteria’s Fe uptake systems against them in a “Trojan-horse” strategy. Various Ga^3+^ complexes gained special attention for the treatment of infections associated with bacterial biofilms^26^. Ga^3+^ readily forms poly-hydroxo complexes in aqueous physiological conditions. Many chelated complexes of Ga^3+^ show antibacterial activity against *Pseudomona aeruginosa* and are stable in physiological conditions^76–83^. In addition to complexation, co-administration of Ga^3+^ salts with known antimicrobial agents, such as a combination of antimicrobial and non-anti-microbial agents, has been described to enhance antimicrobial potency^84,85^. A very small number of Co^3+^ complexes have biochemical roles. The Co^3+^ ion as such is unstable in water but can be stabilized against reduction to Co^2+^ by coordination to ligands or chelators. To date, only the N, O donor ligand type has been found to stabilize the Co^3+^ ion in aqueous conditions. The Co^3+^complexes derived from this ligand donor set have proved to be surprisingly efficient antibacterial or antiviral agents. Some of the most promising classes of Co^3+^ complexes containing N, O donor ligands, like Schiff base [Co(NH_3_)_6_]Cl_3_, are antibacterial and thermally and kinetically stable in aqueous solution, in addition to being easily synthesized^86–88^. The amphibactin is produced by bacteria to specifically chelate Fe^3+^ for biological process and hence the size of metal ion is an important factor. Our earlier analysis on the *E*_*int*_, MIA, chemical hardness, Δ*E*_*H–L*_, and thermodynamic parameters (Tables 1 and 2) for metal ions with similar ionic radii as Fe^3+^ with amphibactin indicate that Ga^3+^ and Co^3+^ can replace and form better complexes than Fe^3+^ and the other metal ions examined. Ga^3+^ has similar ionic radius as Fe^3+^ and is more kinetically stable/inert compared to Fe^3+^ even though it is thermodynamically less stable than Fe^3+^. The Co^3+^ is both kinetically and thermodynamically stable than Fe^3+^. This means that once we replace the Fe^3+^ by Ga^3+^/Co3 + , the resulting amphibactin–Ga(III)/Co(III) complex is kinetically inert for further replacement reaction and the bacterial cell wall will allow its passage as the size is similar to Fe^3+^ ion. Since Fe is essential for the metabolic activities of bacteria and hence for their survival, the better complexation of amphibactin with Ga^3+^ and Co^3+^ than Fe^3+^ (with all three ions having the same ionic radii) will lead to competition for Fe acquisition by bacteria. So instead of Fe; Ga and Co form stable complexes with amphibactin, thereby impairing bacterial metabolic activities and causing cellular toxicity, since the entered Co^3+^ and Ga^3+^ cannot partake in the oxidation–reduction processes inside the cell. The free energy changes for the following reactions were studied

1 $${\text{Co}}{-}{\text{hexamine}} + {\text{amphibactin}} \to {\text{Co}}{-}{\text{amphibactin}} + 6 {{\text{NH}}_{{3}}}$$

2 $${\text{Ga}} - {\text{aqua}} + {\text{amphibactin}} \to {\text{Ga}}{-}{\text{amphibactin}} + 6{{\text{H}}_{{2}}} {\text{O}}$$

3 $$\left[ {{\text{Co}}\left( {{\text{CO}}_{{3}}} \right)_{{3}} } \right]^{{{3} - }} + {\text{amphibactin}} \to {\text{Co}}{-}{\text{amphibactin}} + 3{{\text{CO}}_{{3}}}^{ - }$$

The ∆G with ZPE correction for Eqs. 1 and 2 at b3lyp/def2tzvp level were − 708.52 kcal/mol and − 785.83 kcal/mol, respectively, indicating spontaneity. The reactions (1) and (2) confirmed that the formation of amphibactin–Co^3+^ and amphibactin–Ga^3+^ complexes were thermodynamically feasible. The Co^3+^ was inert in aqueous condition but the replacement of monodentate ligand by multidentate was very favourable thermodynamically due to chelation effect. Entropy was largely responsible for the greater free energy change observed for chelates, compared to complexes of unidentate ligands with the same metal ion. The Co-hexamine was kinetically inert (high ΔE_H–L_ = 6.625) in aqueous solution as it was a low spin *d*^6^ O_h_ complex with high LFSE. Instead of the Co-hexamine we can use [Co(CO_3_)_3_]^3−^ complex (ΔE_H–L_ = 5.14) as it is kinetically less stable or more labile making the forward replacement reaction kinetically feasible. The ∆G + ZPE = − 117.21 kcal/mol for Eq. (3) shows spontaneity^89^.

The Eqs. (1–3) confirm that Co^3+^ from the Co(NH_3_)_6_ complex and Ga^3+^ from the Ga(H_2_O)_6_ complex readily binds to amphibactin, forming thermodynamically stable amphibactin–Co and amphibactin–Ga complexes, which inhibit Fe uptake by the bacteria. Also, the different parameters calculated (Tables 1, 2, 3) confirms the Co^3+^ and Ga^3+^ forms stronger complexes with amphibactin. Thus Ga^3+^ and Co^3+^ emerge as good antibacterial agents and can be impressed as Trojan horses for marine bacteria *Vibrio* sp. HC0601C5, especially those producing amphibactin siderophores. This way of exploiting the nutritional pathways of bacteria to quell infection is an effective strategy to stop infection by introducing Co and Ga into the amphibactin–Fe^3+^ complex has potential therapeutic benefit and paves the way for the development of new antibiotics based on Co and Ga. Indeed, Co^3+^ and Ga^3+^ compounds coupled with known antibiotic agents emerge as potential tools to tackle sea food or waterborne infections.

## Discussions

Here we studied the stability, affinity and energetics of amphibactin complexed with different transition metal ions in + 3 oxidation state in an O_h_ coordination sphere. The Δ*E*_*H–L*_, interaction energy **(**E_*int*_), metal ion affinity (MIA), relaxation energy **(**δ) and thermodynamic parameters (∆G, ∆H, ∆S, ∆S_*trans*_, ∆S_*rot*_ and ∆S_*vib*_) calculated indicate that out of the different metal ions studied (Tables 1, 2, 3) Fe^3+^, Ga^3+^ and Co^3+^ with similar ionic radii form stable complexes with amphibactin. The Mn^3+^ also forms strong complex with amphibactin, but the Mn^3+^ ion is unstable in aqueous condition. The free energy changes calculated for the studied process: reduction of Fe^3+^ → Fe^2+^, followed by protonation and subsequent hydrolysis, were found to be spontaneous, thereby making this the favored mechanism of Fe release from the chelated amphibactin–Fe^3+^ complex into the bacterial cell. We examined the energetics involved in the mechanistic pathway of Fe acquisition, followed by its reduction, protonation and subsequent release into the bacterial cell in order to understand the scope of blocking bacterial infection by hindering this process. Of the transition metal ions listed in Table 2, Co^3+^ and Ga^3+^ showed better chelation for amphibactin other than Fe^3+^. Here Ga^3+^ and Co^3+^, both with same ionic radius as Fe^3+^, competed with Fe^3+^ and formed better complexes, thereby impairing the capacity of bacteria to take up Fe and effectively starving the cells. These findings pave the way for Co^3+^ and Ga^3+^ as new anti-bacterial agents for bacteria-producing amphiphilic siderophores, amphibactin. The amphibactin–Co^3+^ forms strongest complex compared to other complexes studied thereby making it the best candidate as anti-bacterial agent. Our results also exemplify the potential to utilize Co^3+^ and Ga^3+^ akin to a Trojan horse to effectively block Fe acquisition by bacteria, enabling their specific and effective sterilization irrespective of their antibiotic-resistance type.

## Methods

Density functional theory (DFT) calculations using the GAUSSIAN 09 suite of programs were carried out for all the amphibactin–metal complexes^90^. Geometry optimization of all the structures studied was performed using the hybrid density functional B3LYP^91–94^ and M06^95–97^ levels of theory with all atoms with DEF2TZVP basis set^98,99^, as implemented in the GAUSSIAN 09 quantum chemistry package. Calculations were done as unrestricted DFT calculation for ground state spin 2S + 1 > 1 and for 2S + 1 = 1 restricted DFT calculation respectively. Out of the twelve amphibactin–metal complexes studied five (Fe^2+^, Fe^3+^, Mn^3+^, Cr^3+^ and V^3+^) were high spin and remaining were low spin complexes. The input structure of amphibactin is made maintaining the (DDLL) configuration at asymmetric carbon centres as given in Fig. 1a. The lipidic side chain was replaced by hydrogen in amphibactin to reduce the computational expense. We did geometry optimization on this amphibactin structure followed by harmonic vibrational frequency analysis. To find the optimized minimum energy structure vibrational harmonic frequency analysis had been done. Vibrational frequency analysis did not show any imaginary frequencies indicating that the optimized minimum energy structure was reached. This optimized minimum energy amphibactin structure (DDLL) was complexed with metal ions and chosen as input structures for further optimization of the complexes. The gas phase contribution to the thermochemistry parameters Gibbs Free energy G, Enthalpy H, Entropy S, etc., were determined using DFT calculations. The HOMO–LUMO gaps, chemical hardness (η), interaction energy (E_*int*_), metal ion affinity (MIA), and relaxation energies were further calculated using appropriate equations. The E_int_, MIA, relaxation energies were ZPE corrected. The E_int_ was calculated after correcting the basis set super-position error (BSSE) by the counterpoise procedure (CP) of Boys and Bernardi^100^. Geometry optimization was done, followed by vibrational frequency analysis of amphibactin–Fe^2+^ and amphibactin–Fe^3+^ at 298 K and 1 atm., in aqueous medium (dielectric ε = 78.4) using the polarized continuum (overlapping spheres) model (CPCM), where a calculation was performed in the presence of a solvent by placing the solute in a cavity within the solvent reaction field^101,102^.

## Supplementary information

Supplementary Information.
