# Supplementary material for: Insights into the chemistry of the amphibactin–metal (M3+) interaction and its role in antibiotic resistance
Source: Sci Rep. 2020 Dec 3;10:21049. doi: 10.1038/s41598-020-77807-3 (PMC7712776; doi:10.1038/s41598-020-77807-3)
Supplement: Supplementary file 1 — Supplementary Information. [file 41598_2020_77807_MOESM1_ESM.docx]

**Supplementary Information**

**Insights into the chemistry of the amphibactin-metal (M^3+^) interaction and its role in antibiotic resistance**

Vidya Kaipanchery,^1,2*^ Anamika Sharma,^1,2^ Fernando Albericio,^2,3,4*^ and Beatriz G. de la Torre^1^*

^1^KwaZulu-Natal Research Innovation and Sequencing Platform (KRISP), School of Laboratory Medicine and Medical Sciences, College of Health Sciences, University of KwaZulu-Natal, Durban 4041, South Africa; ^2^Peptide Science Laboratory, School of Chemistry and Physics, University of KwaZulu-Natal, Durban 4001, South Africa; ^3^CIBER-BBN, Networking Centre on Bioengineering, Biomaterials and Nanomedicine, and Department of Organic Chemistry, University of Barcelona, 08028 Barcelona, Spain; ^4^Institute for Advanced Chemistry of Catalonia (IQAC-CSIC), 08034 Barcelona, Spain

**Figure S1**: Optimized geometries of different amphibactin-metal complexes at B3LYP/M06/def2tzvp level. All the structures are drawn using BIOVIA, Dassault Systèmes, Discovery Studio Visualizer, version: 17.2.0.16349, 2017. [https://www.3ds.com/products-services/biovia/products/molecular-modeling-simulation/biovia-discovery-studio/visualization/](https://www.3ds.com/products-services/biovia/products/molecular-modeling-simulation/biovia-discovery-studio/visualization/%20) and PerkinElmer Informatics, ChemBioDraw UltraVersion:14.0.0.117. <https://cambridgesoft.com/Ensemble_for_Chemistry/details/Default.aspx?fid=14&pid=666>.

| 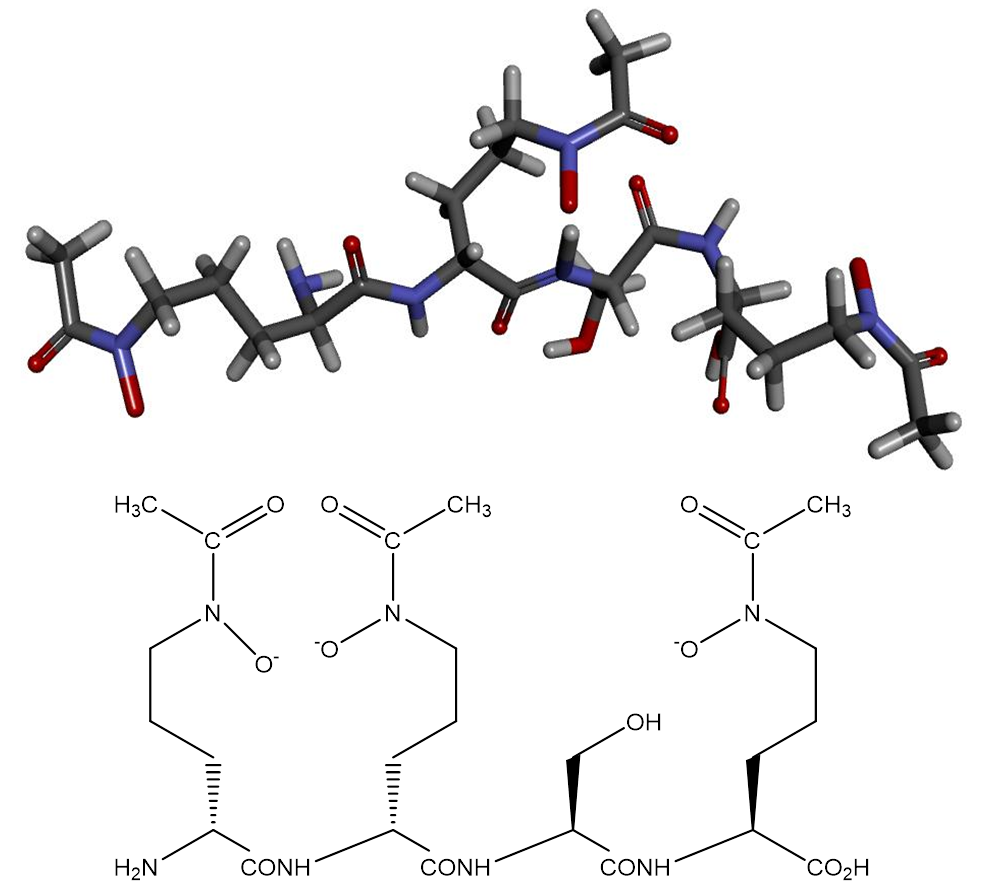  1 Amphibactin |  |
| --- | --- |
|  |  |
|  |  |
| 2 Co(III)-amphibactin 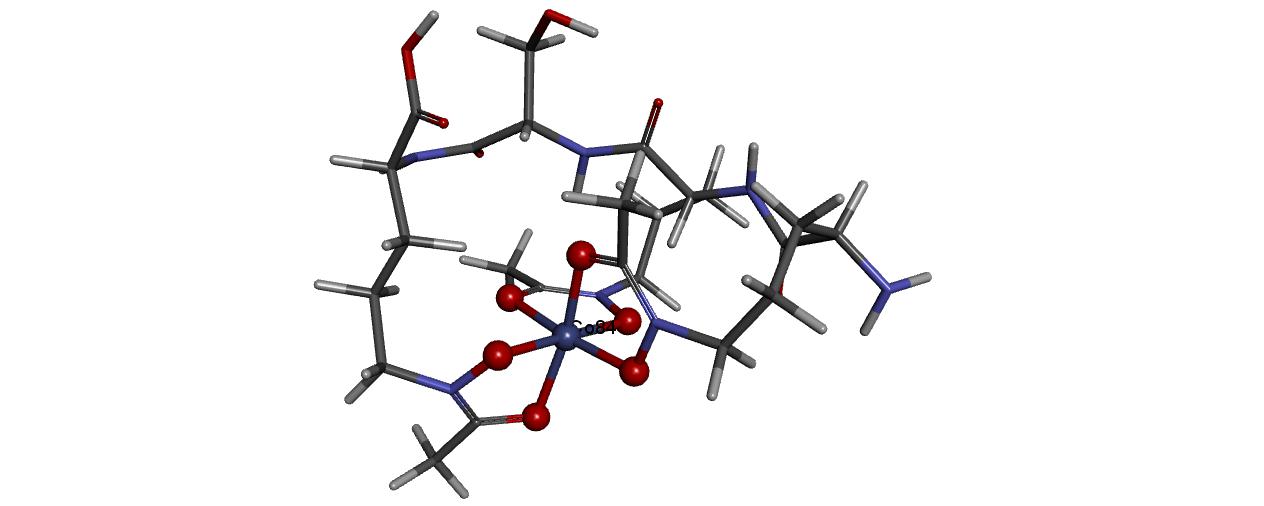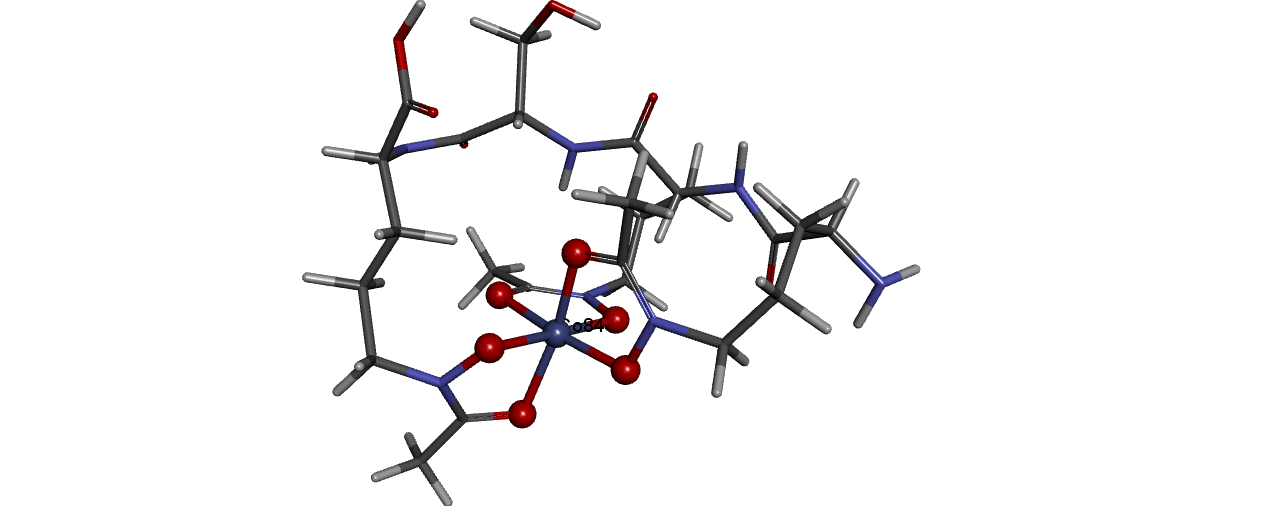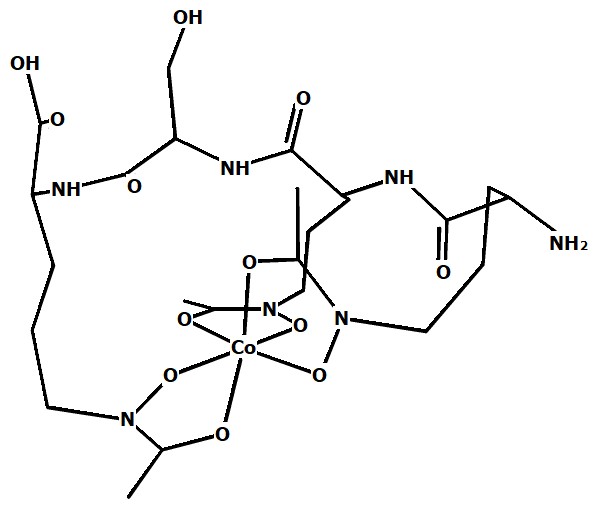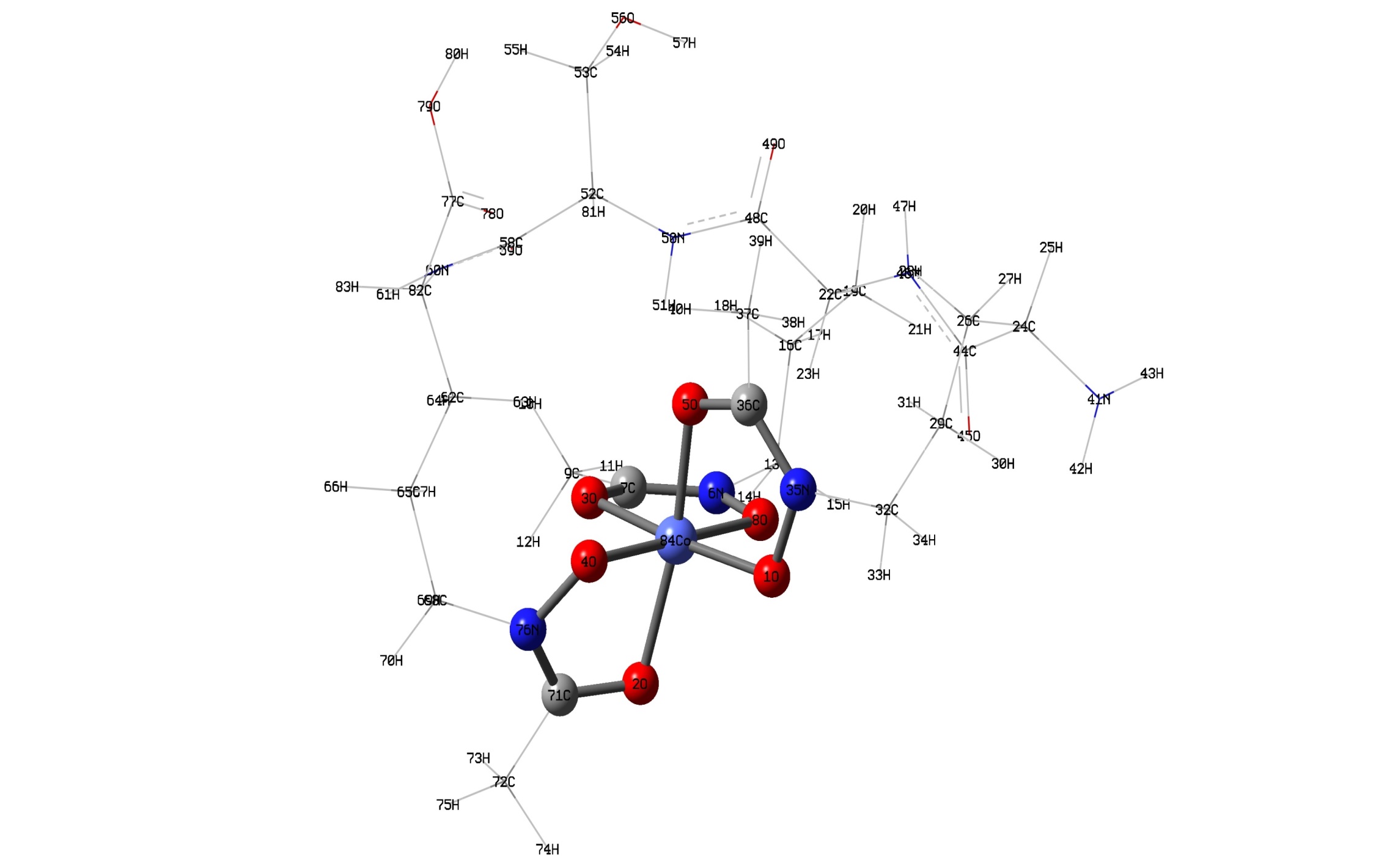 **B3LYP/DEF2TZVP**  **M06/DEF2TZVP** |  |
|  |  |
|  |  |
|  | |


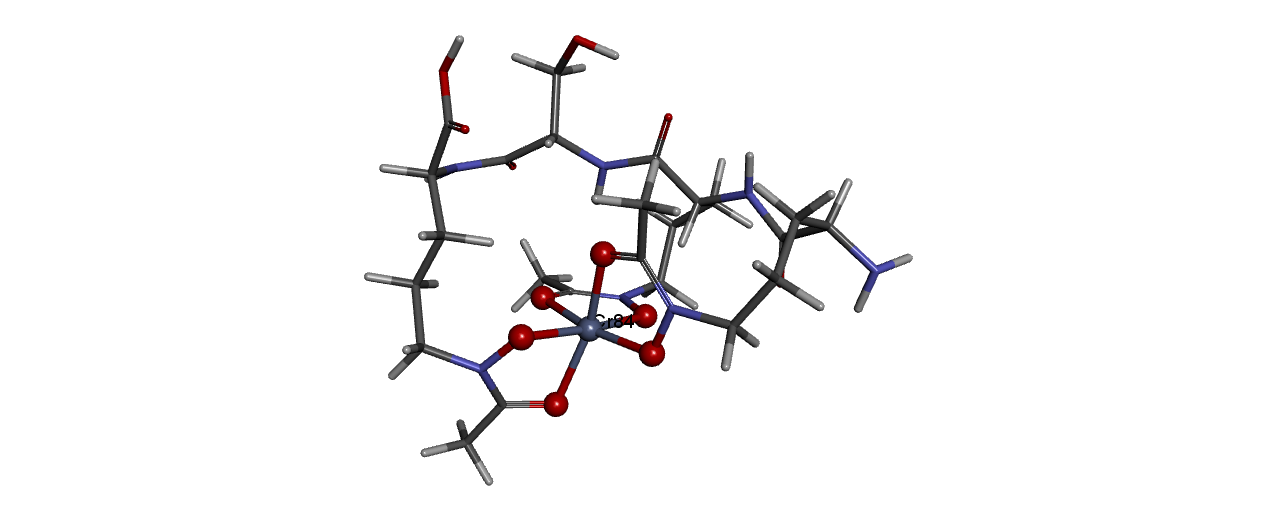

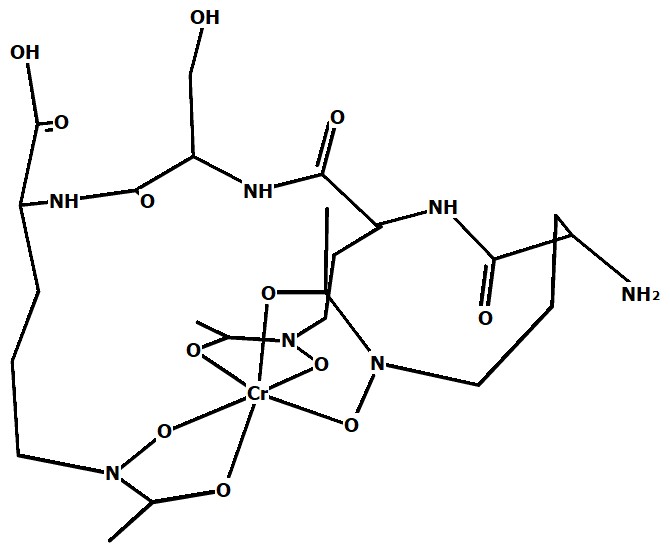

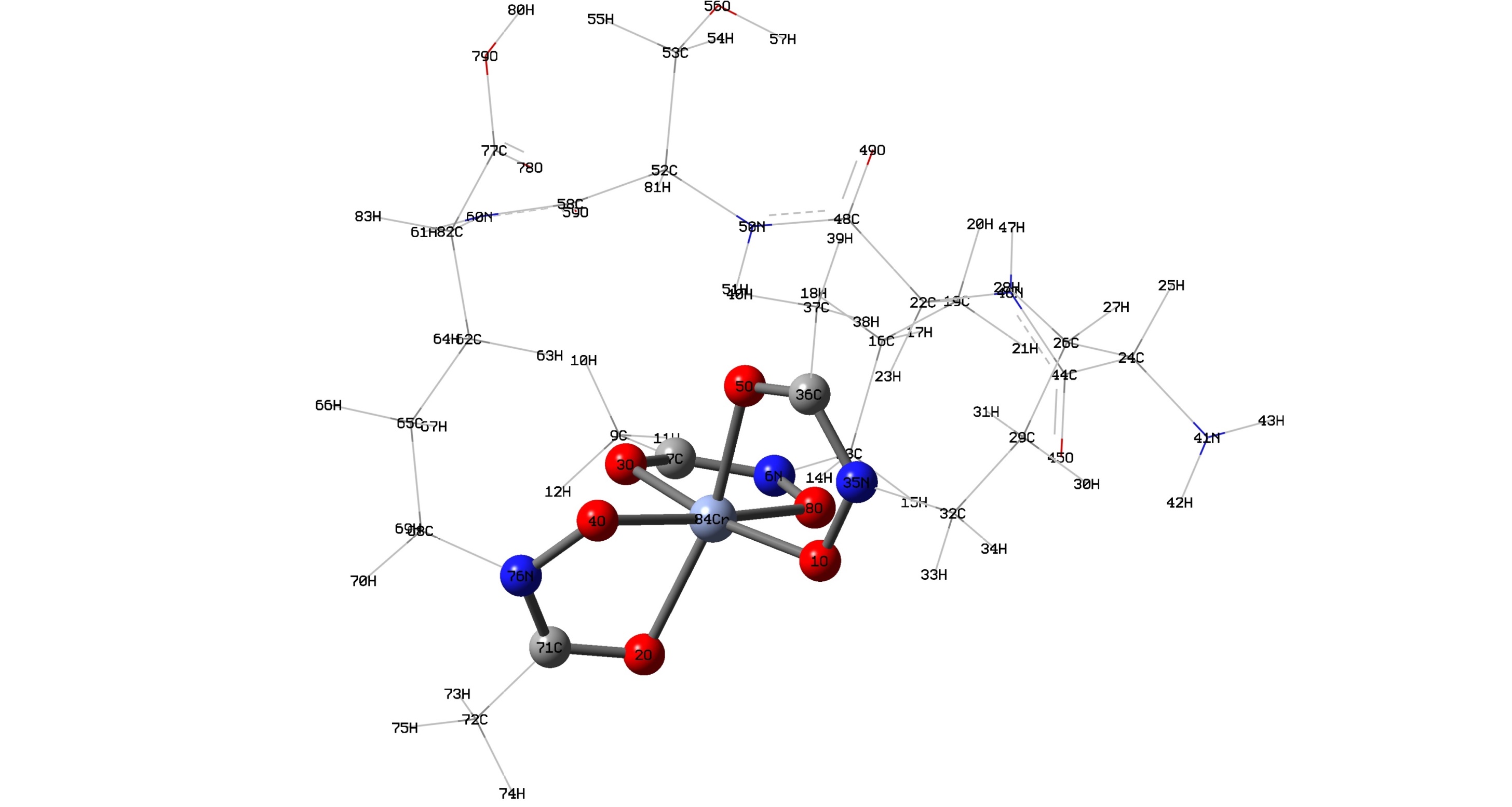

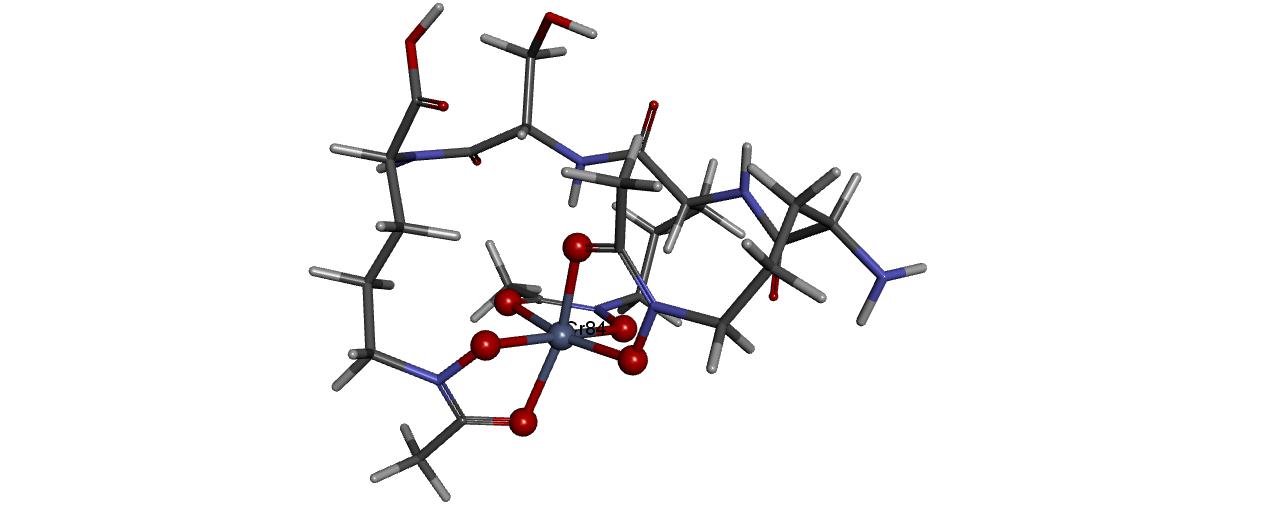


**B3LYP/DEF2TZVP**

**M06/DEF2TZVP**

3 Cr(III)-amphibactin

4 Ga (III) - amphibactin


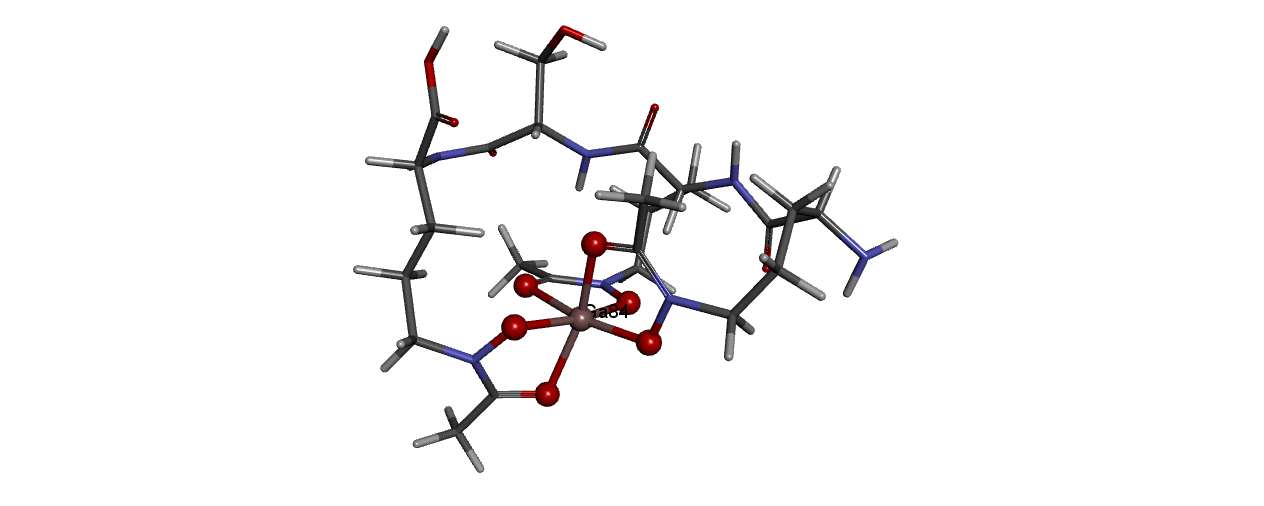

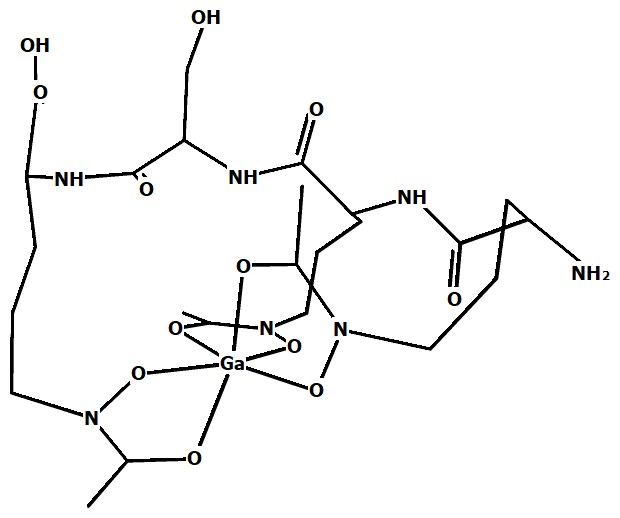

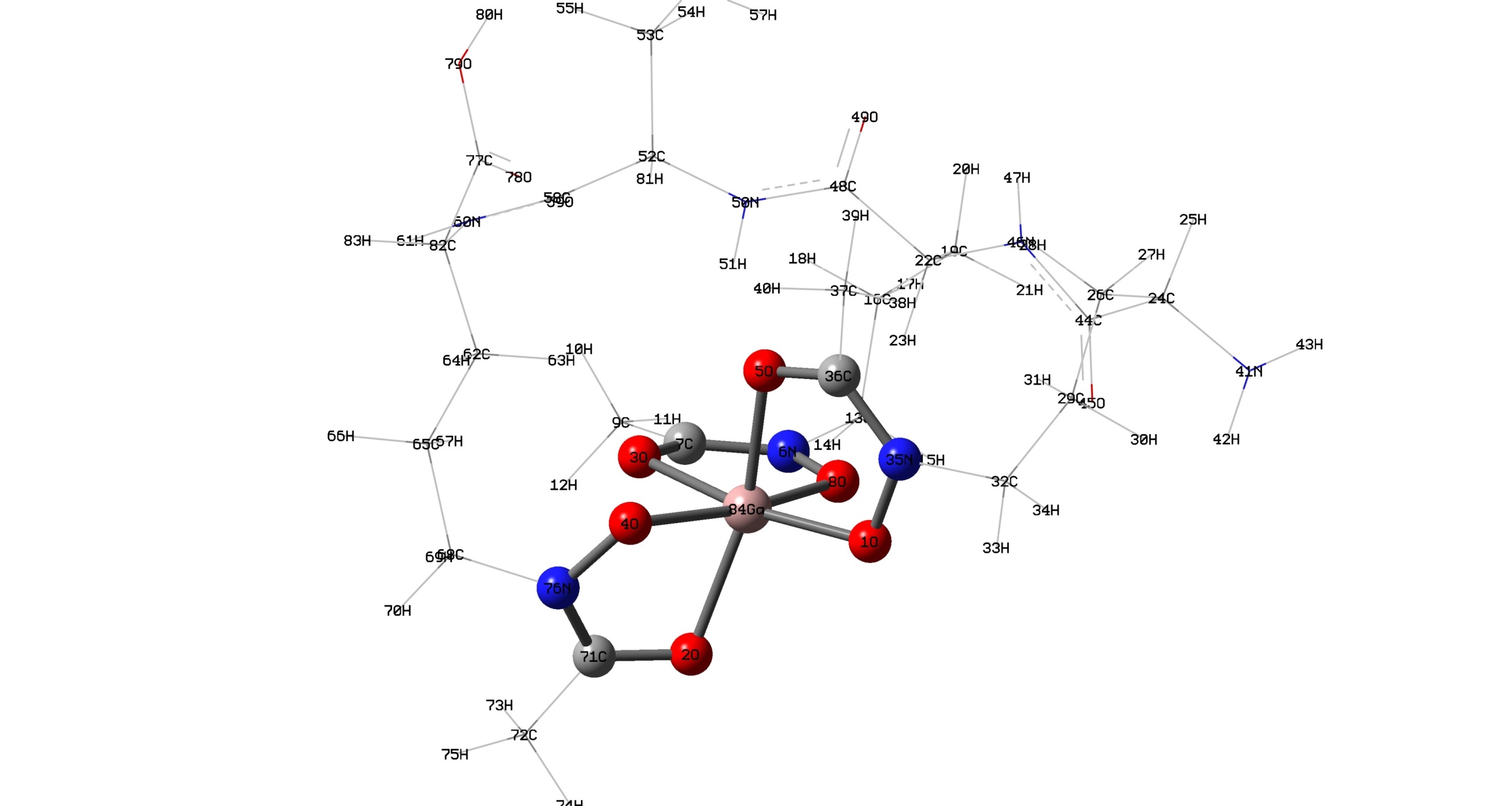

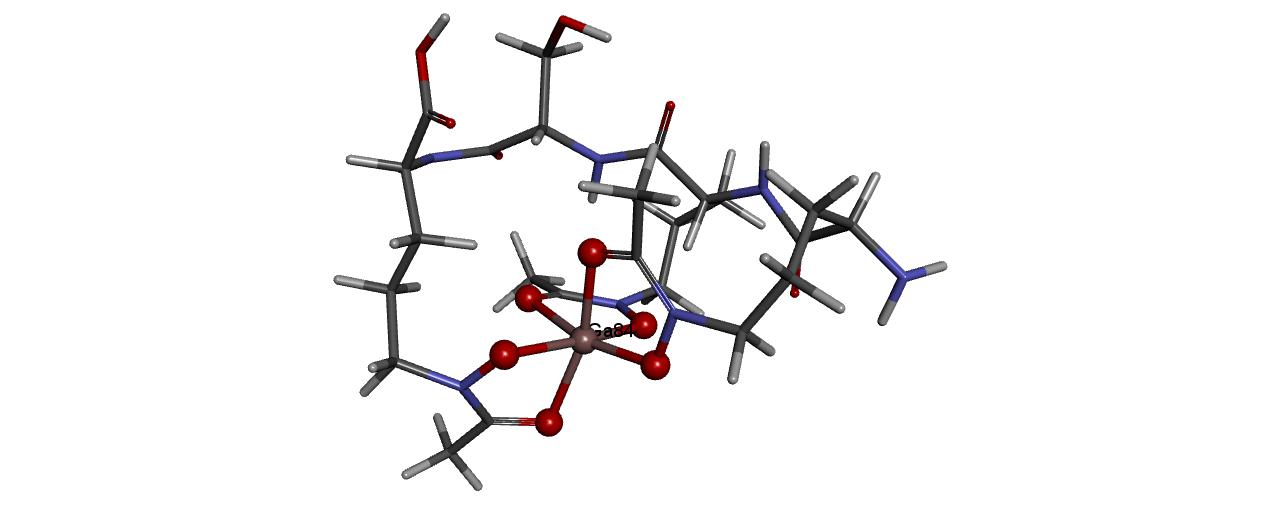


**B3LYP/DEF2TZVP**

**M06/DEF2TZVP**

5 Fe(II) - amphibactin


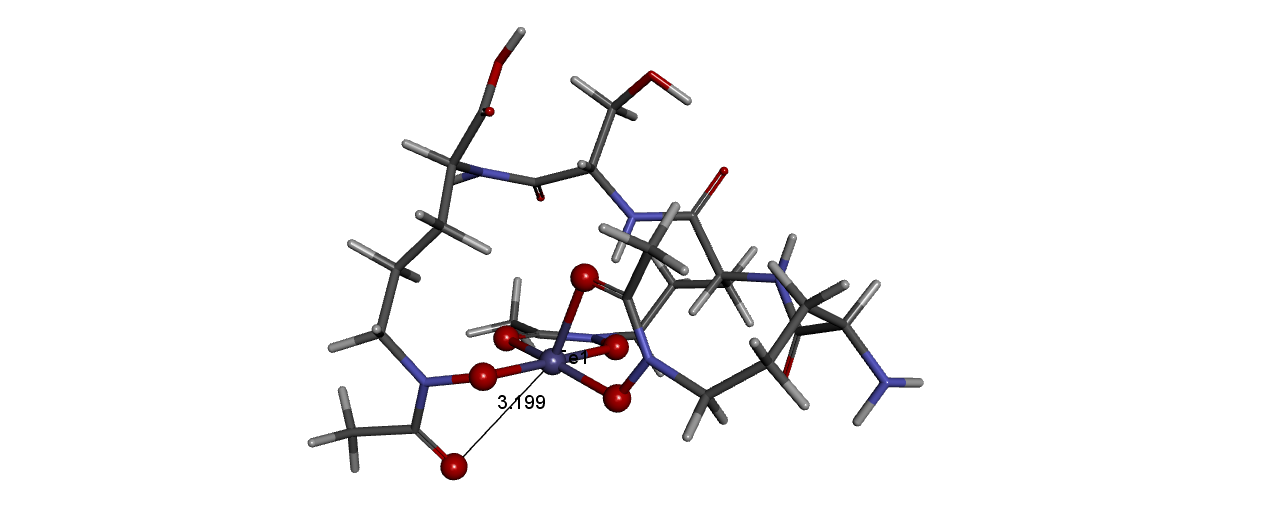


**FeII**


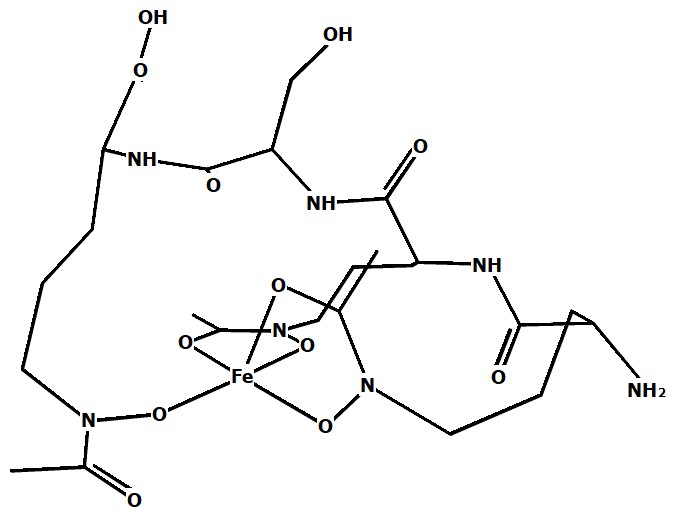

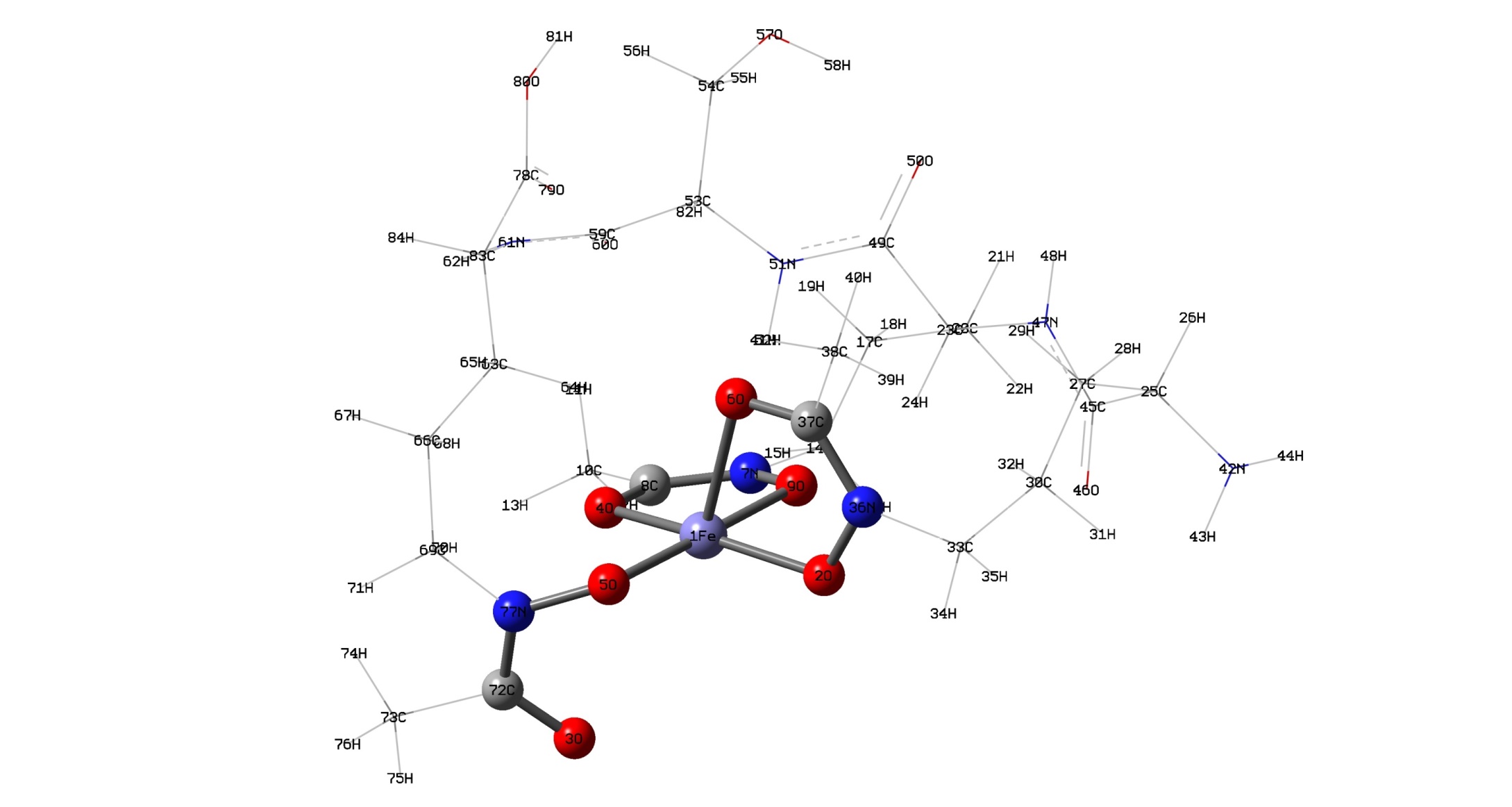

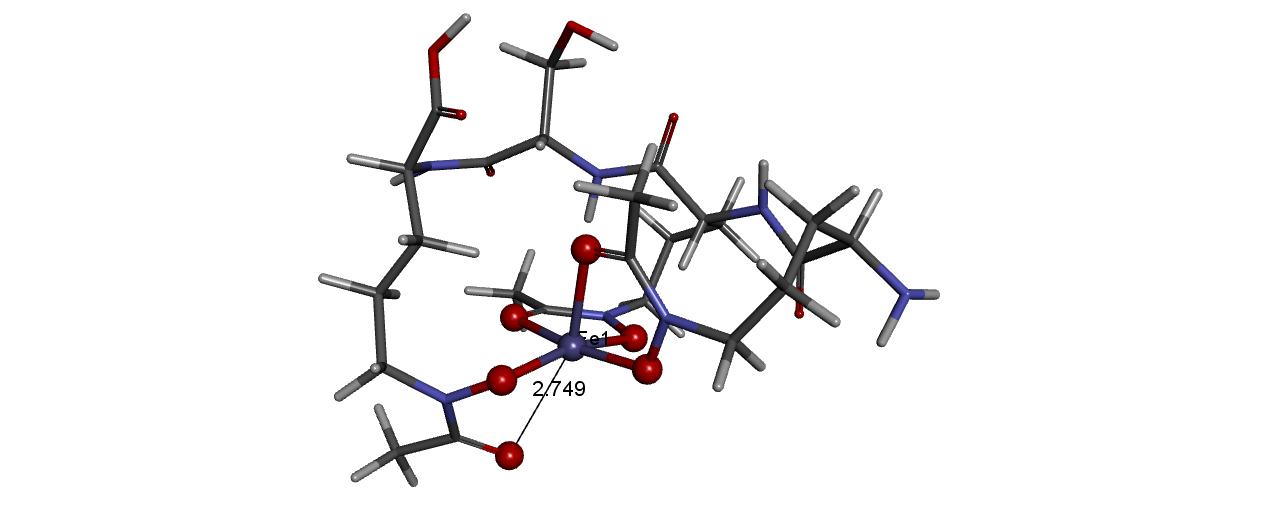


**B3LYP/DEF2TZVP**

**M06/DEF2TZVP**

6 Mn(III) - amphibactin


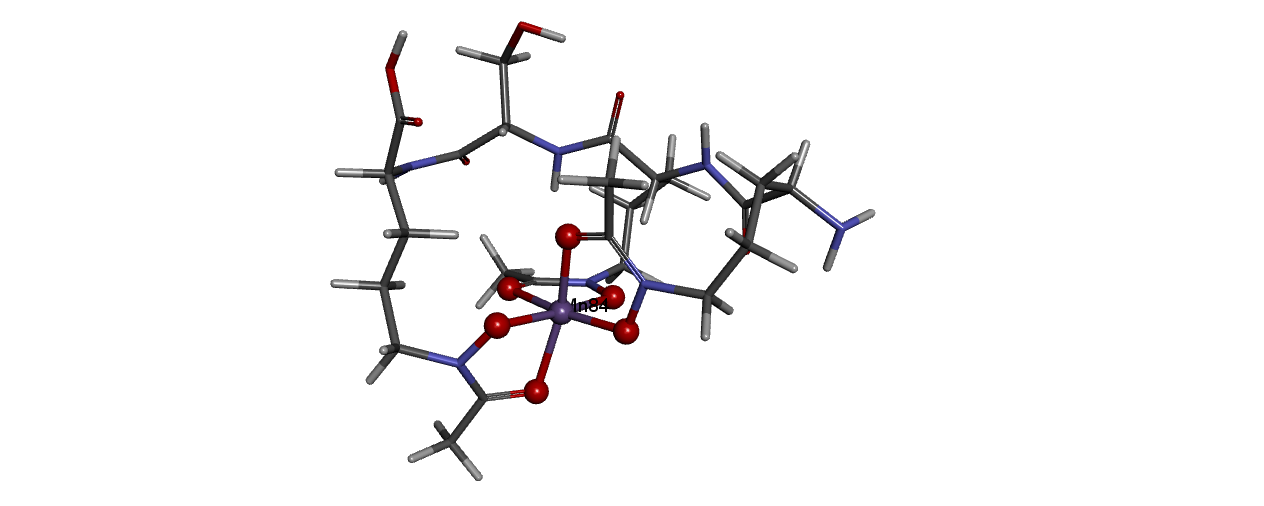

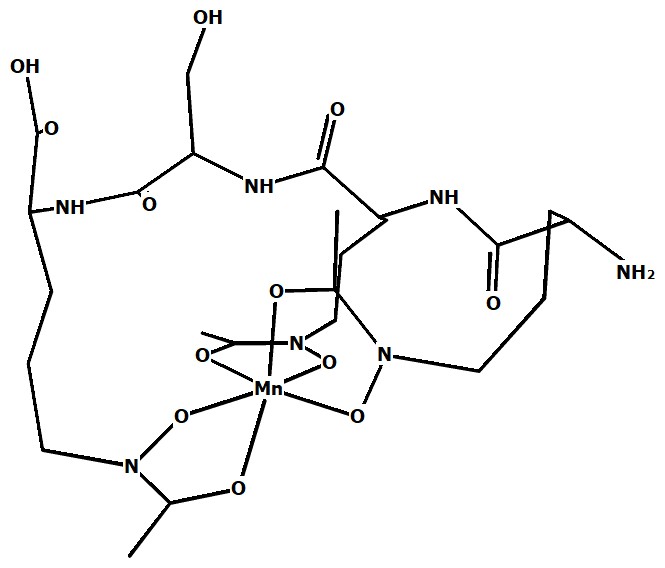

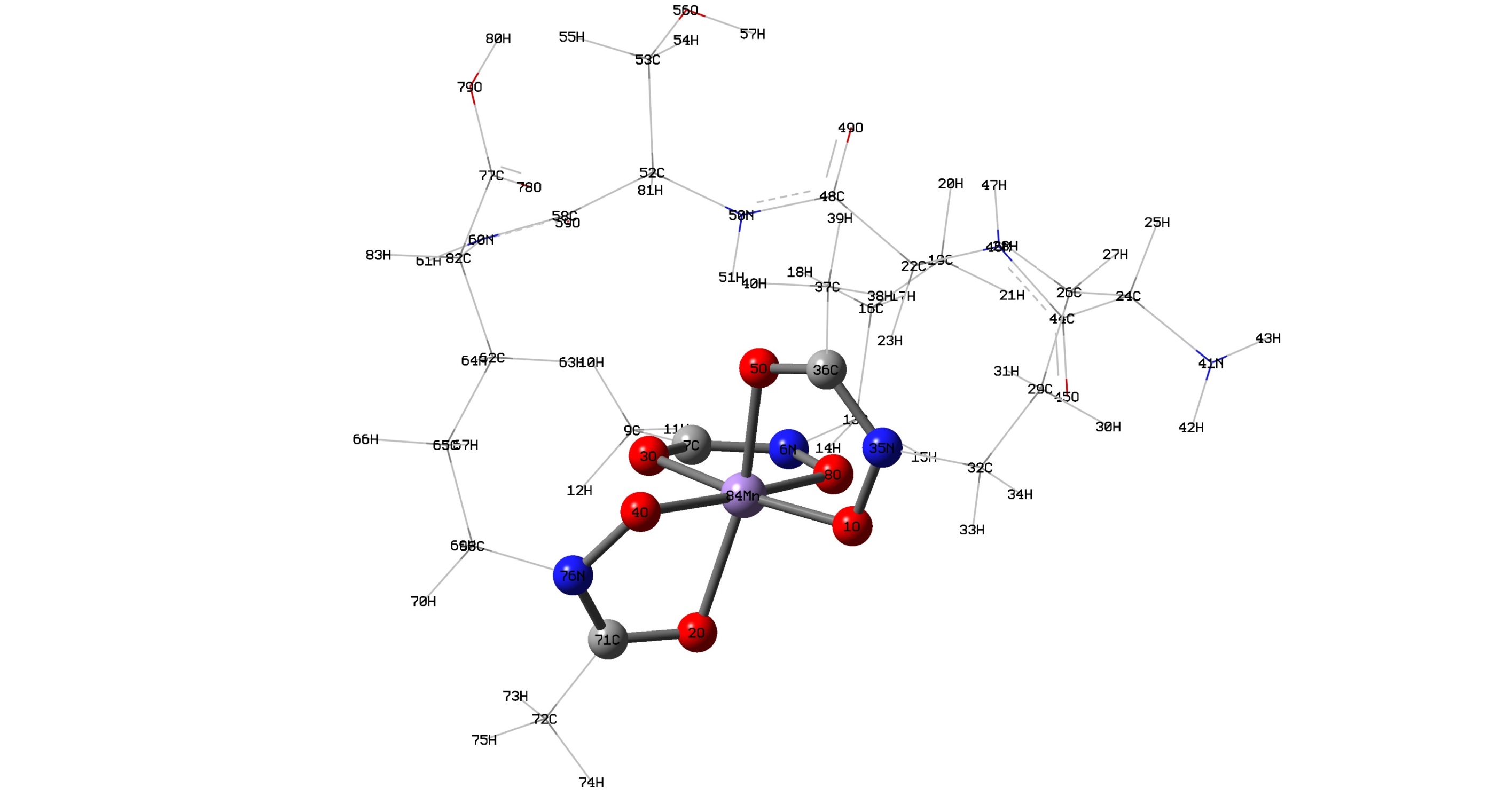

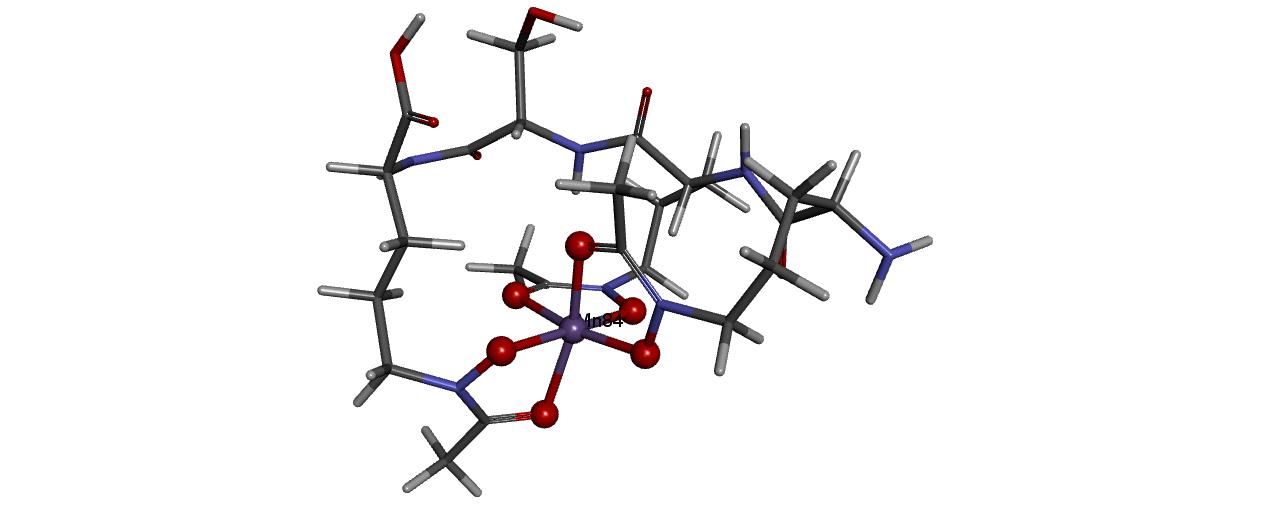


**B3LYP/DEF2TZVP**

**M06/DEF2TZVP**


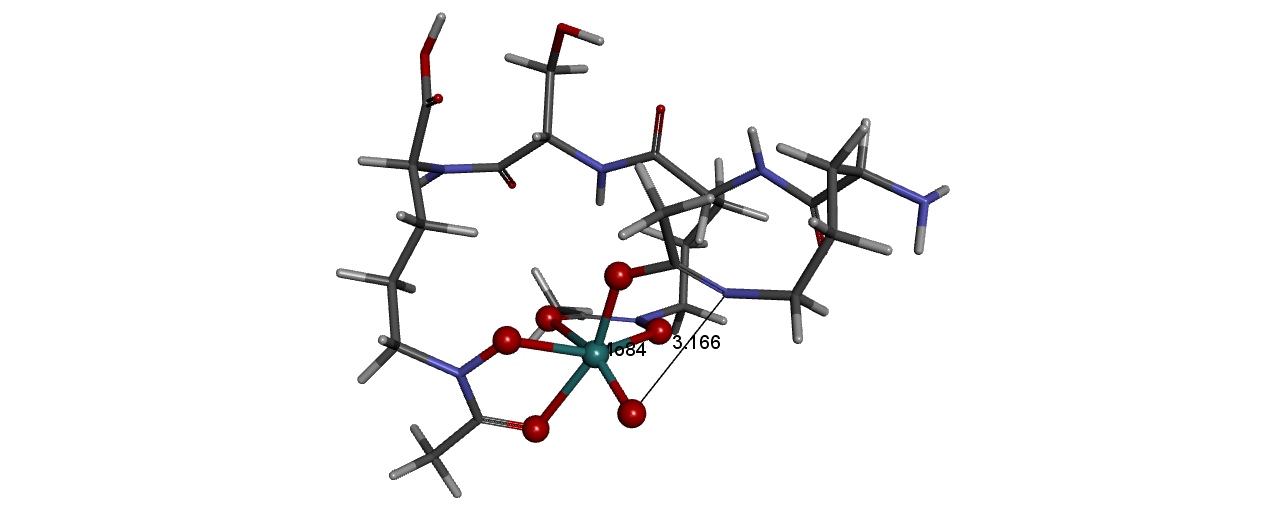

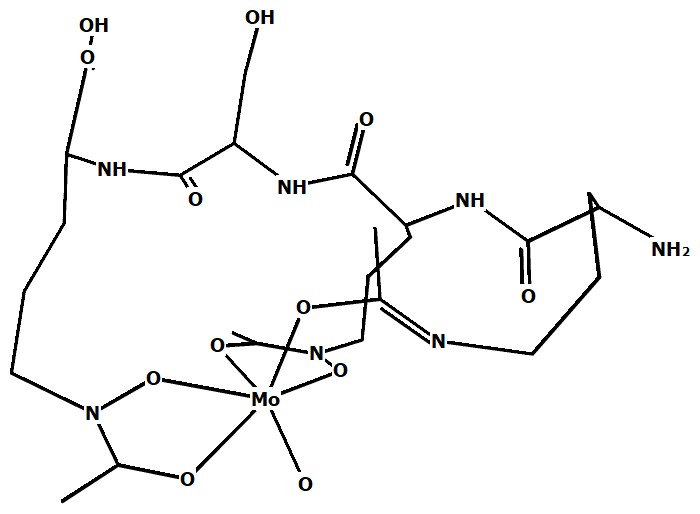

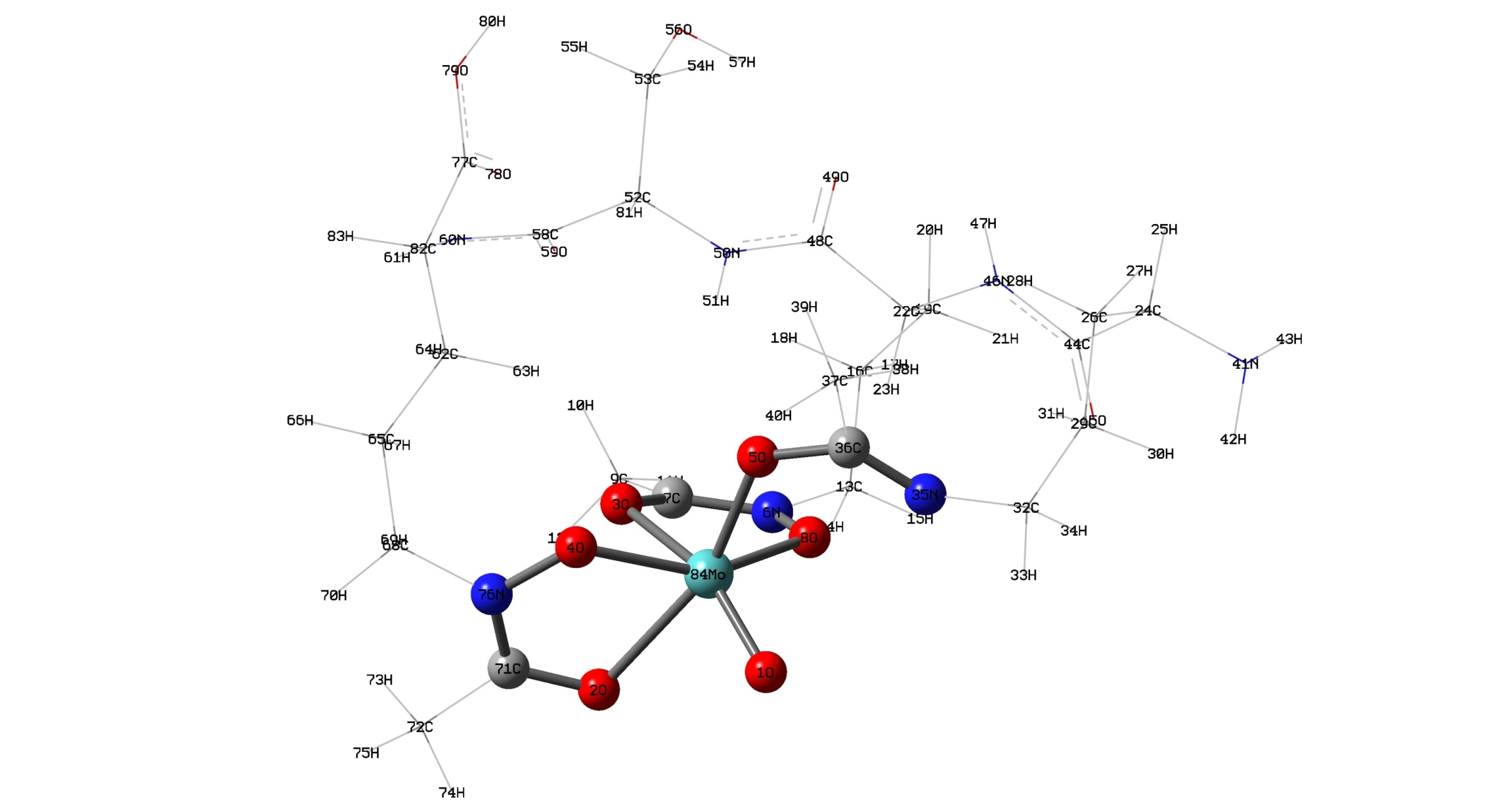

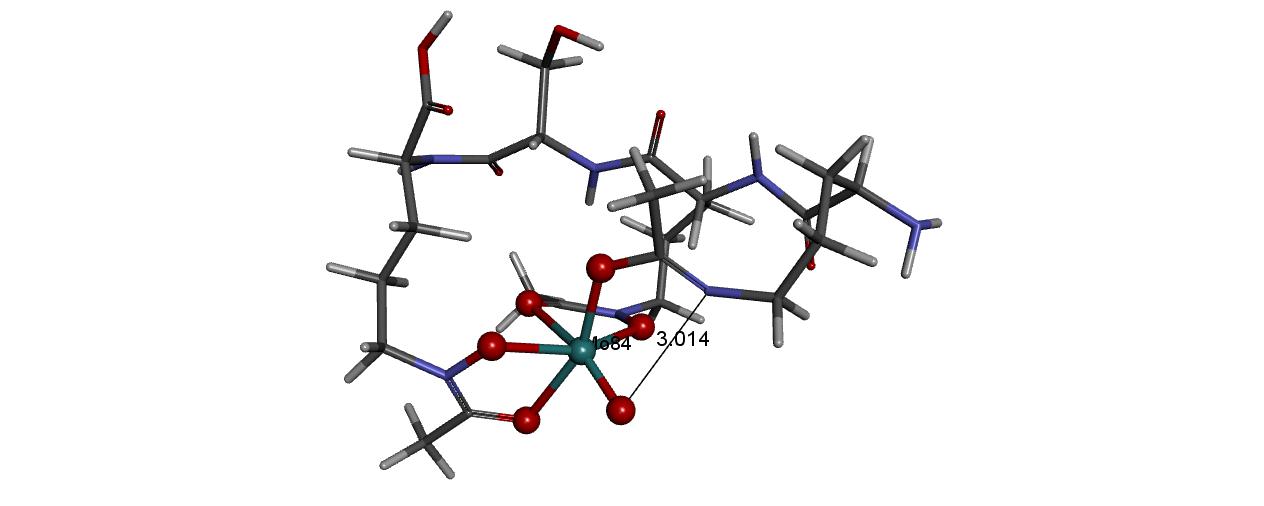


**B3LYP/DEF2TZVP**

**M06/DEF2TZVP**

7 Mo(III) - amphibactin


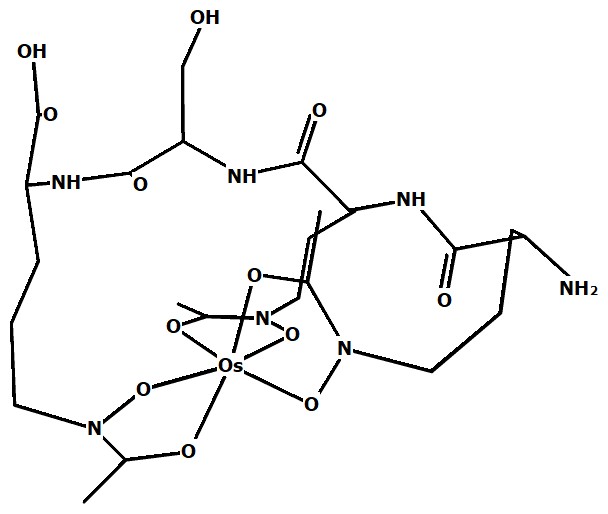

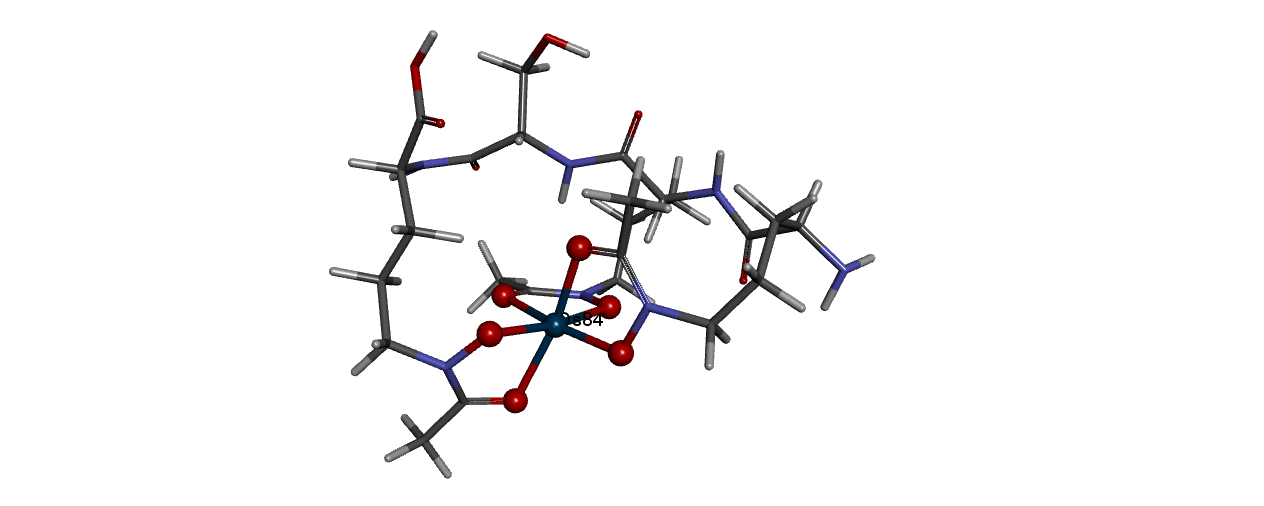

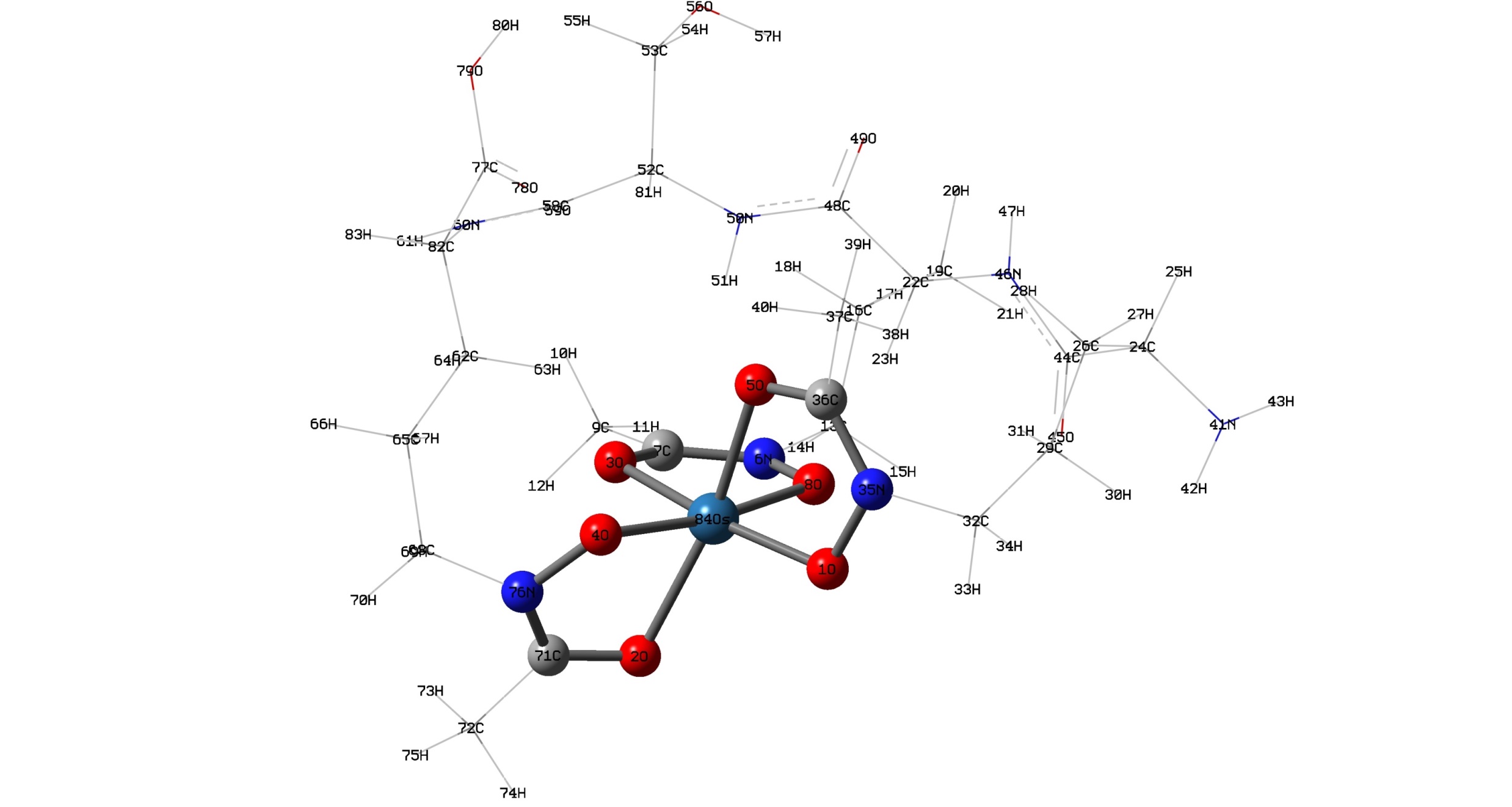

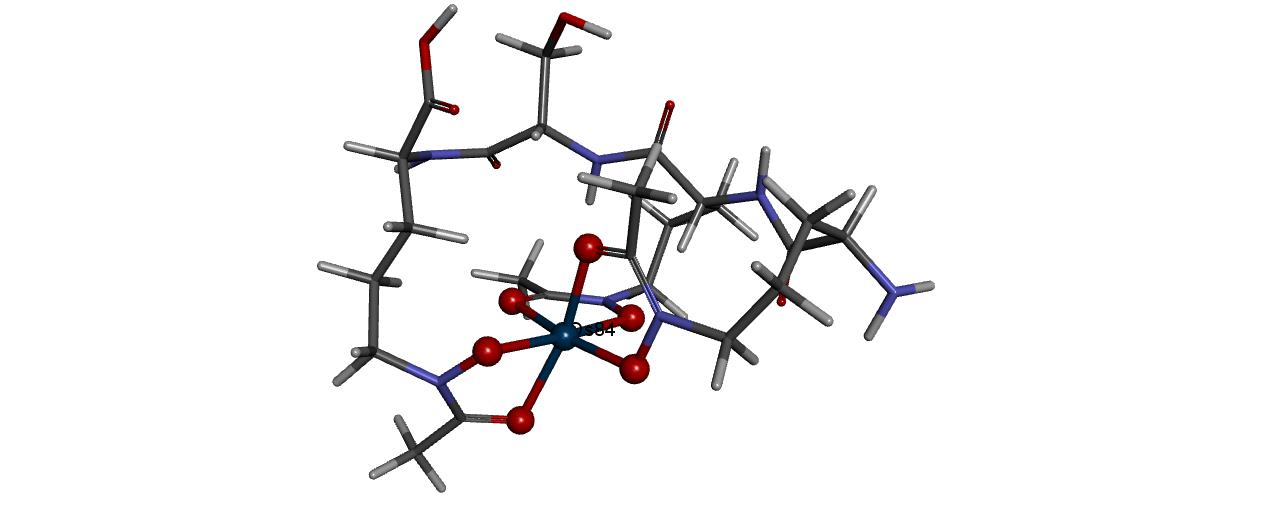


**B3LYP/DEF2TZVP**

**M06/DEF2TZVP**

8 Os(III) - amphibactin

9 Ru(III) - amphibactin


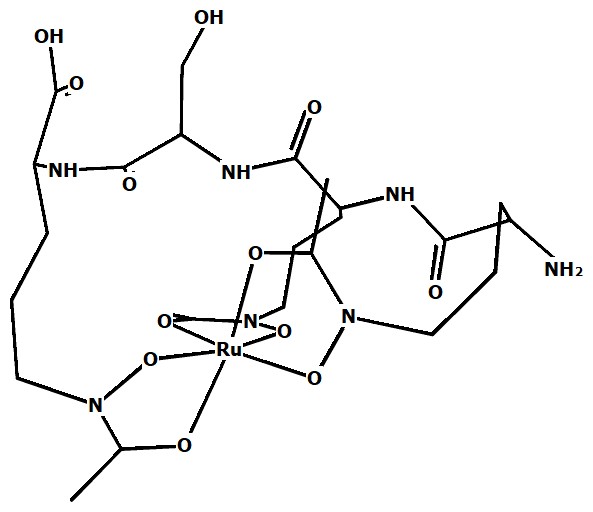

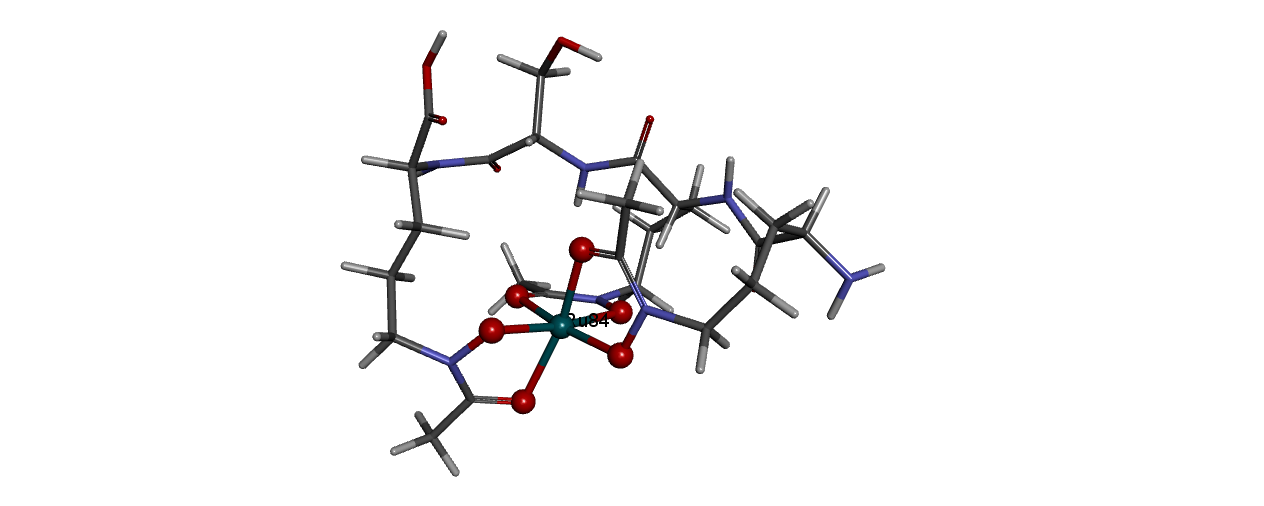

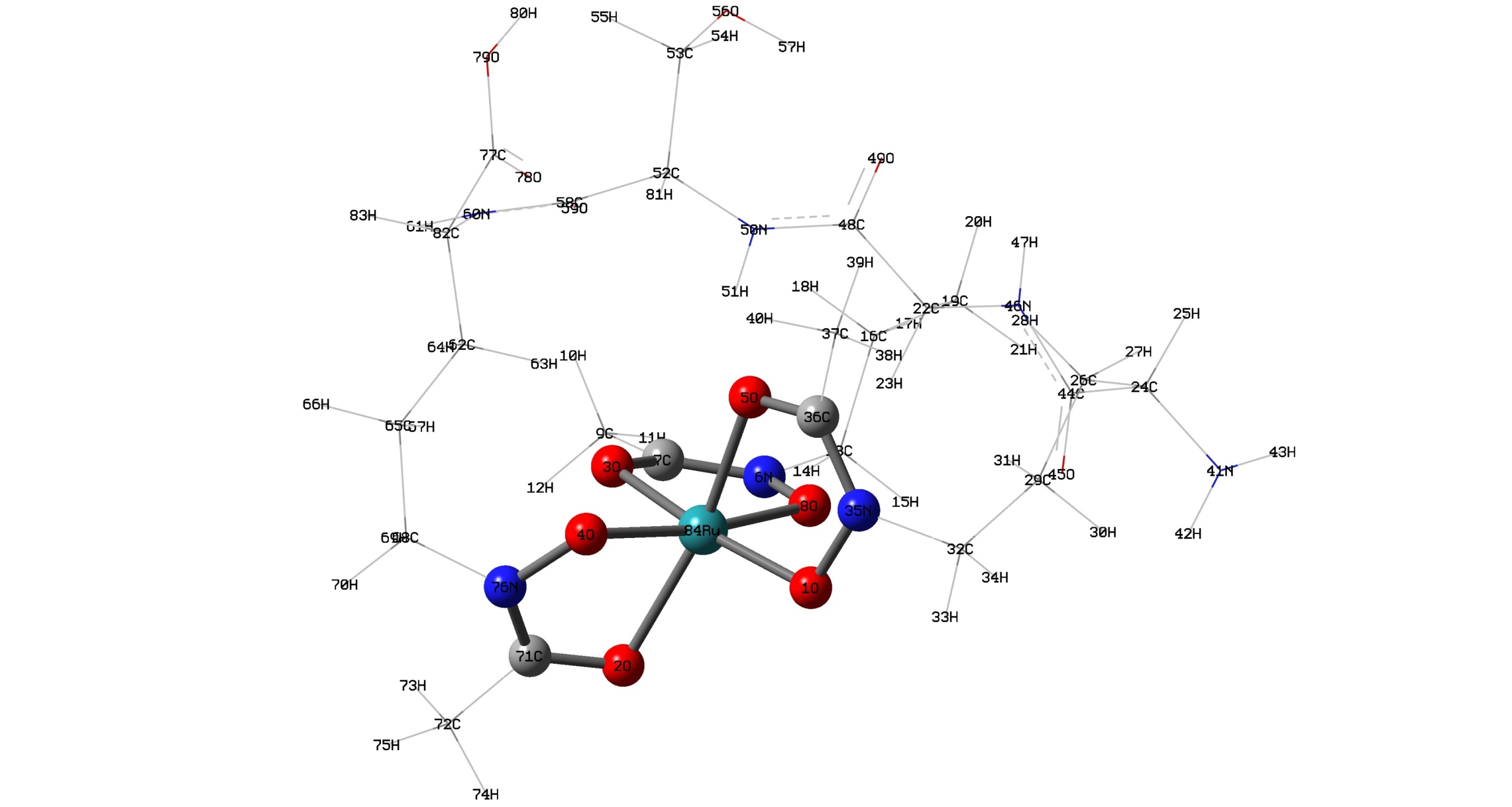

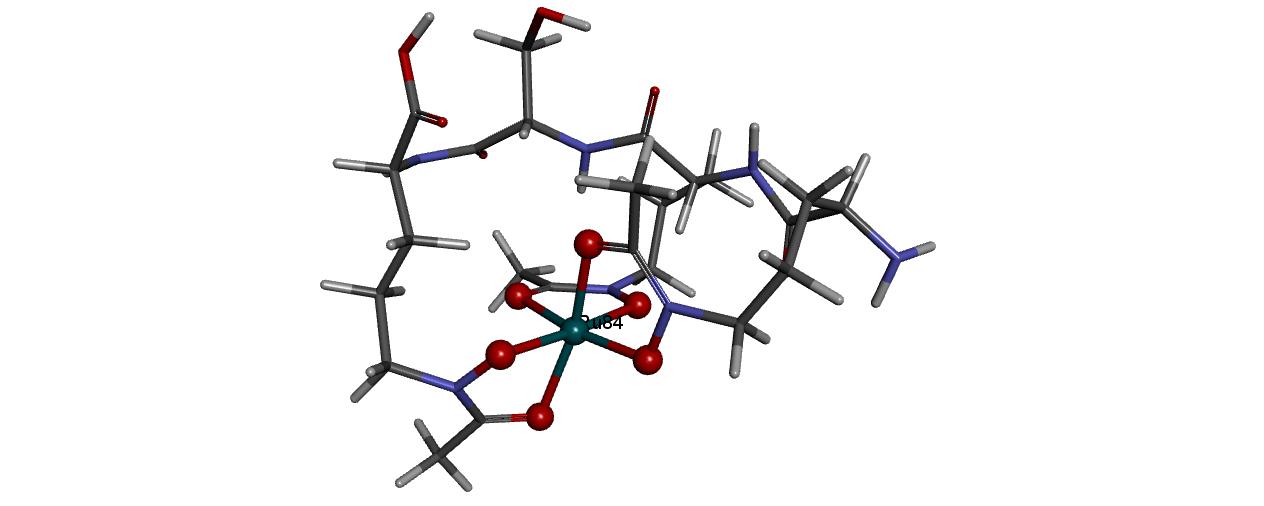


**B3LYP/DEF2TZVP**

**M06/DEF2TZVP**

10 Sc(III) - amphibactin


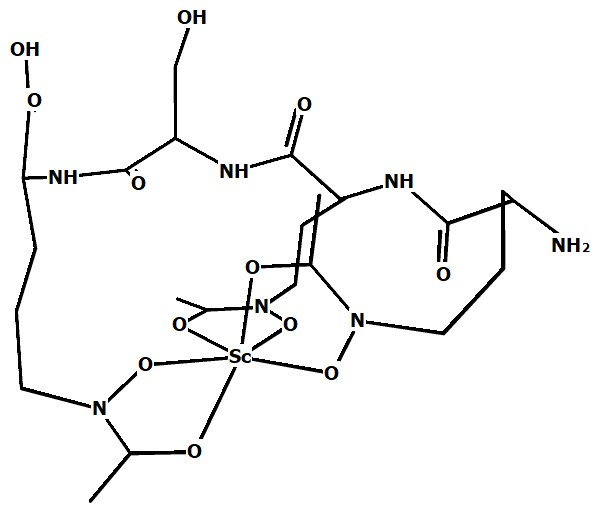

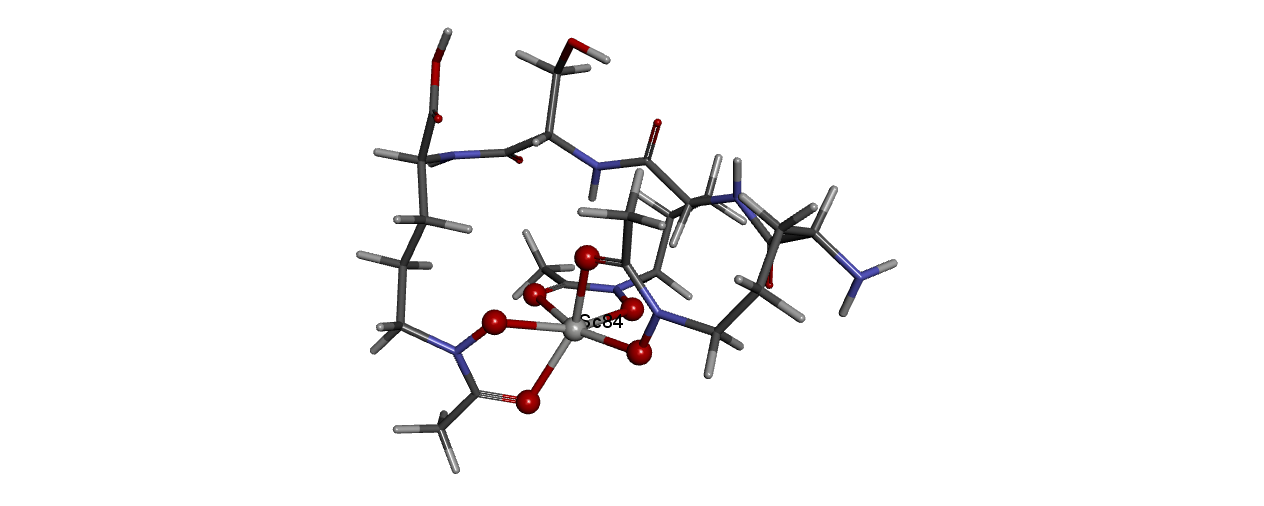

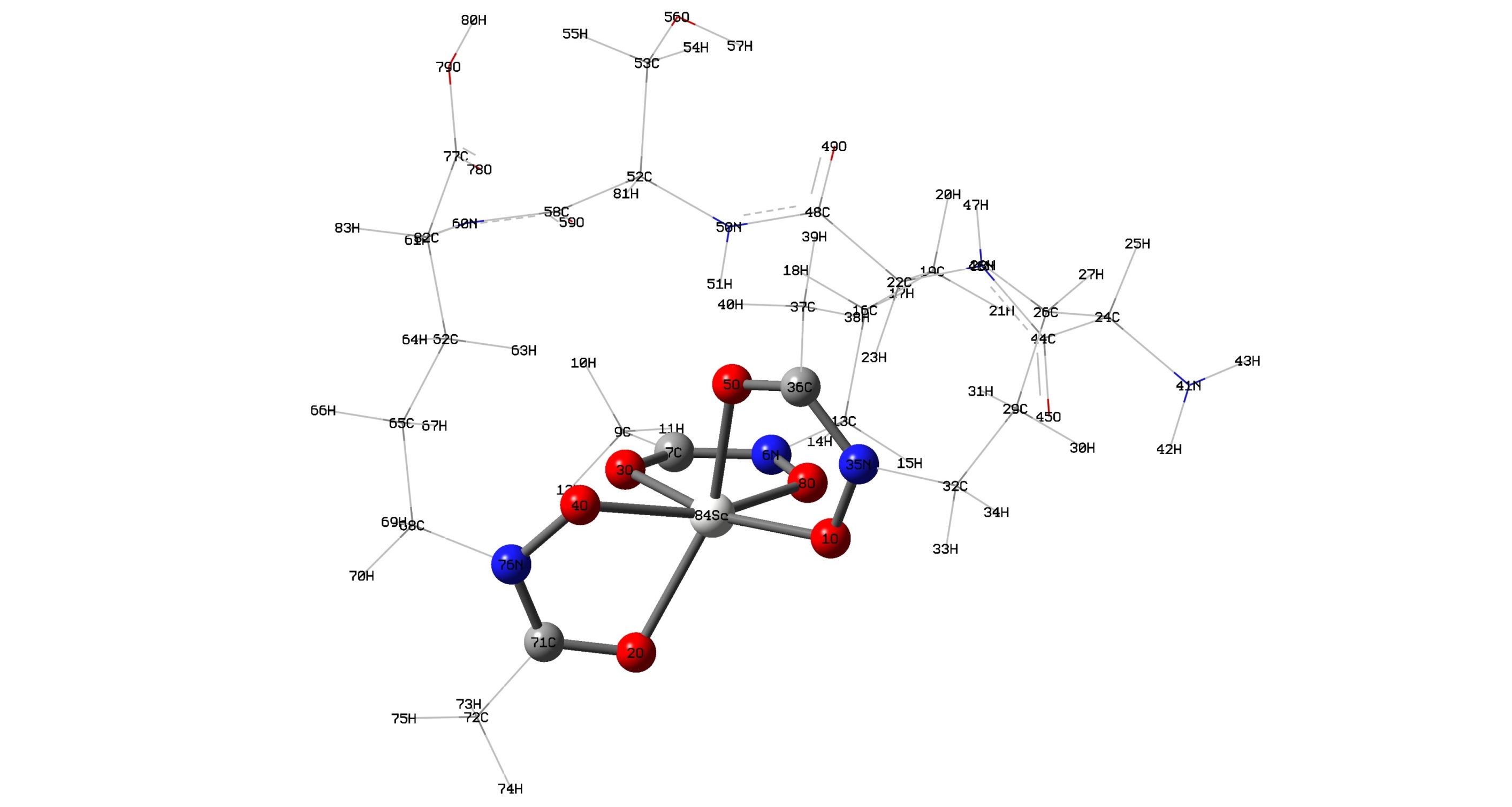

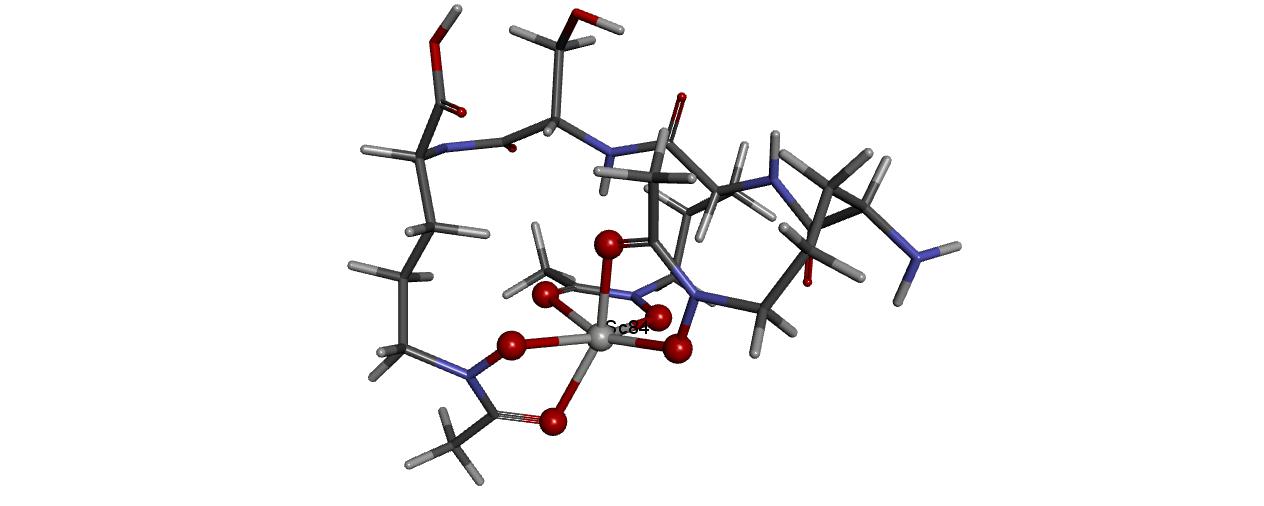


**B3LYP/DEF2TZVP**

**M06/DEF2TZVP**

11 Ti(III) - amphibactin


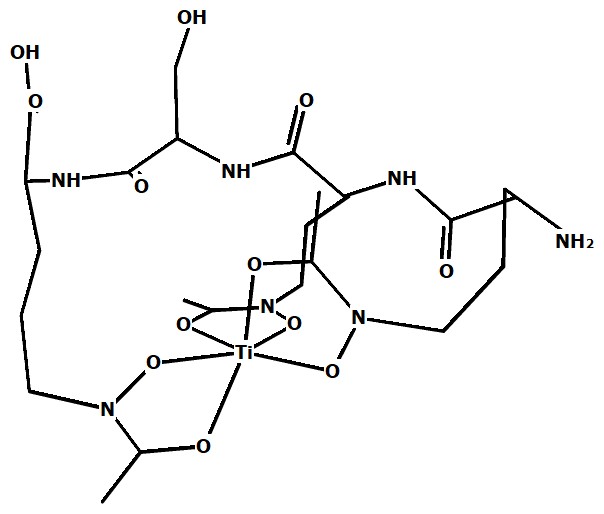

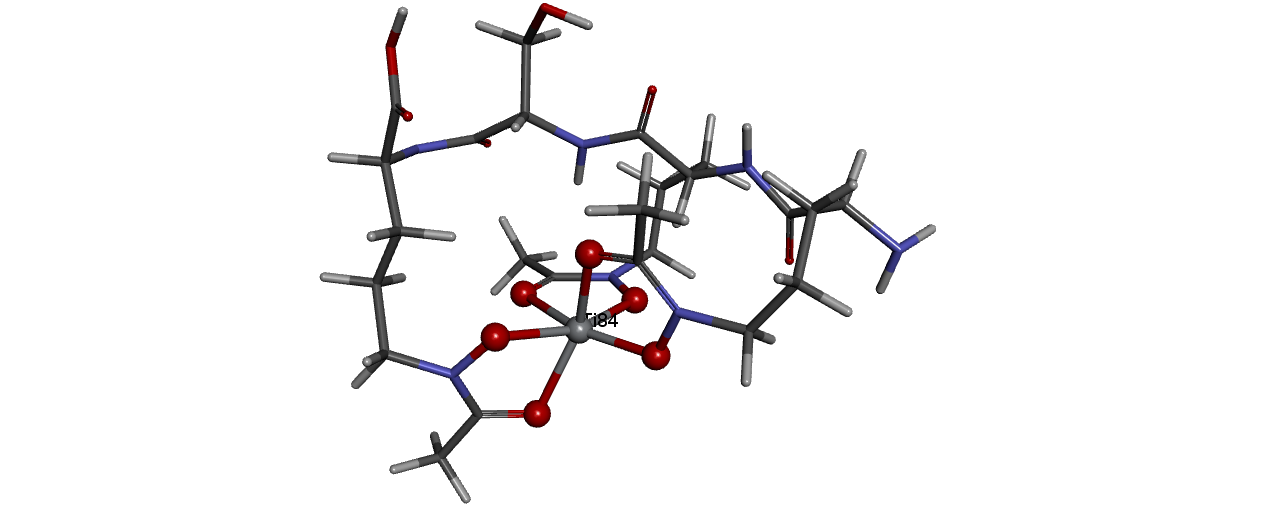

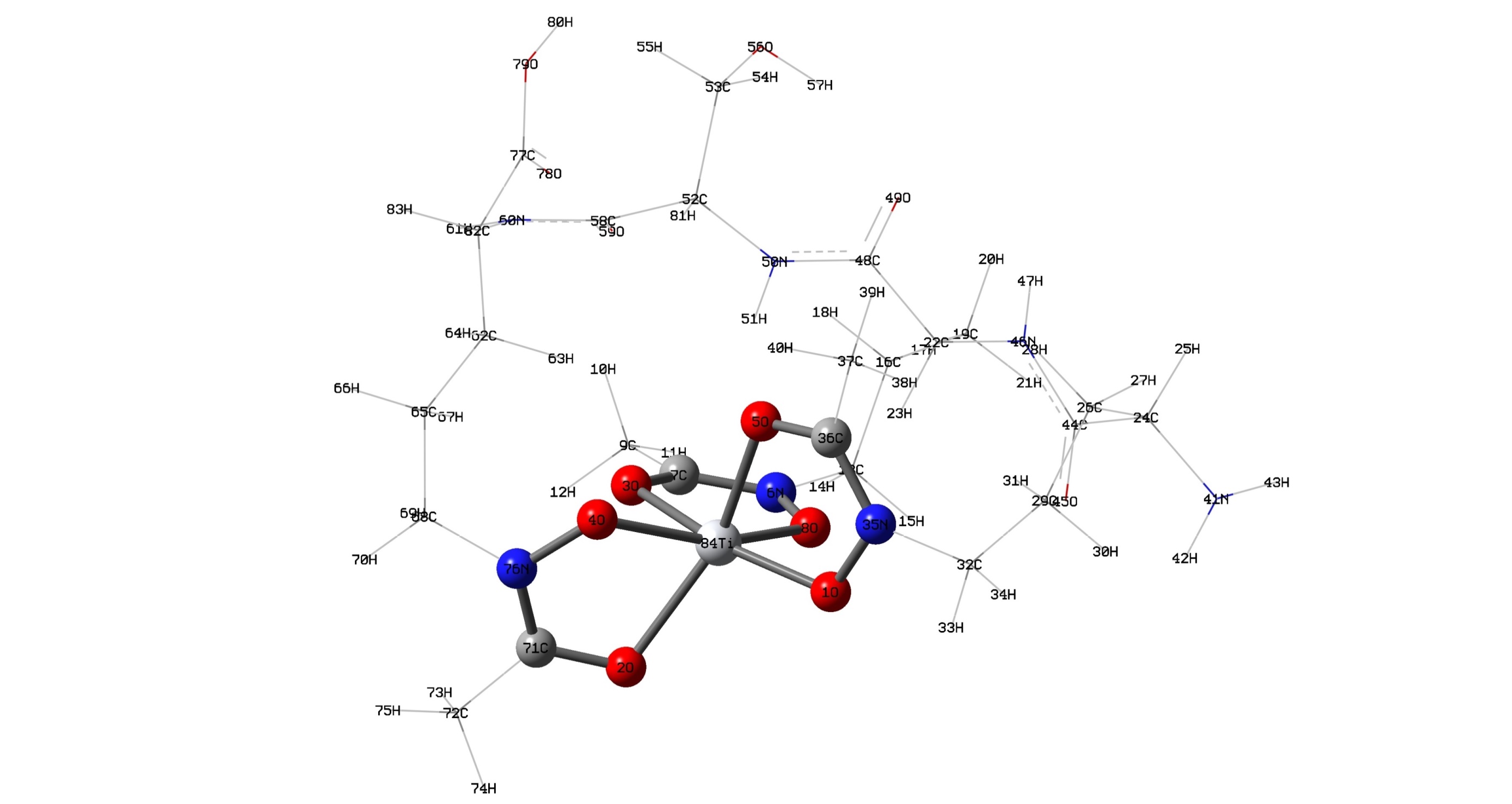

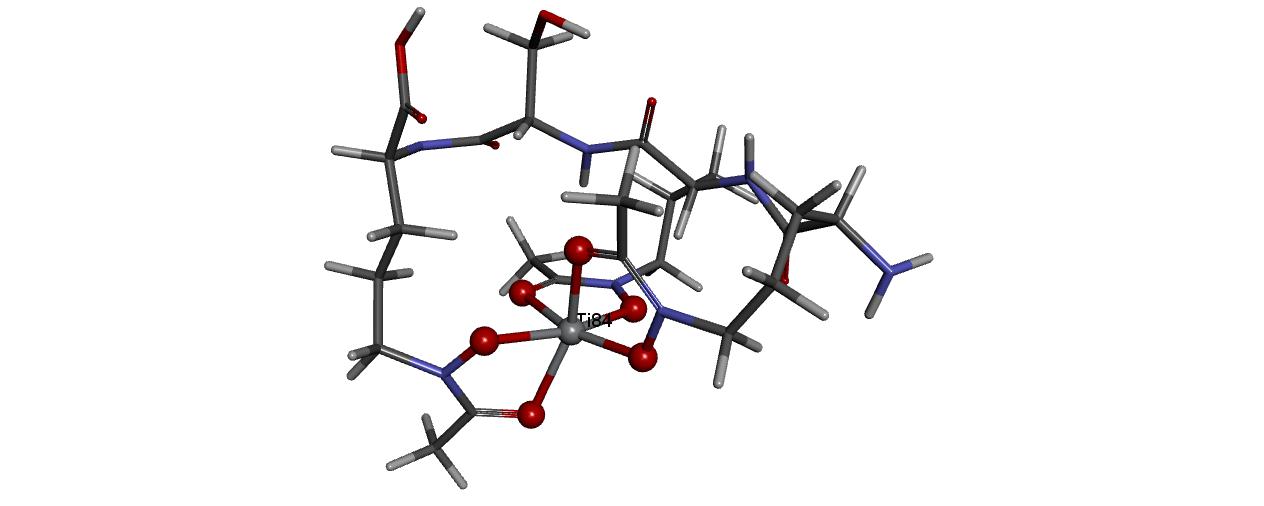


**B3LYP/DEF2TZVP**

**M06/DEF2TZVP**

12 V(III) - amphibactin


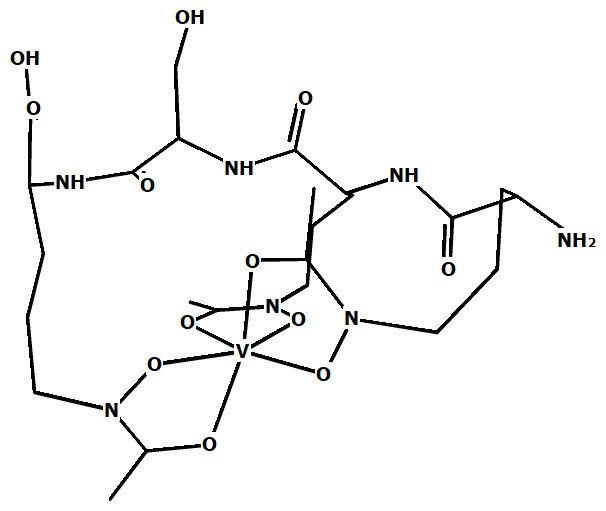

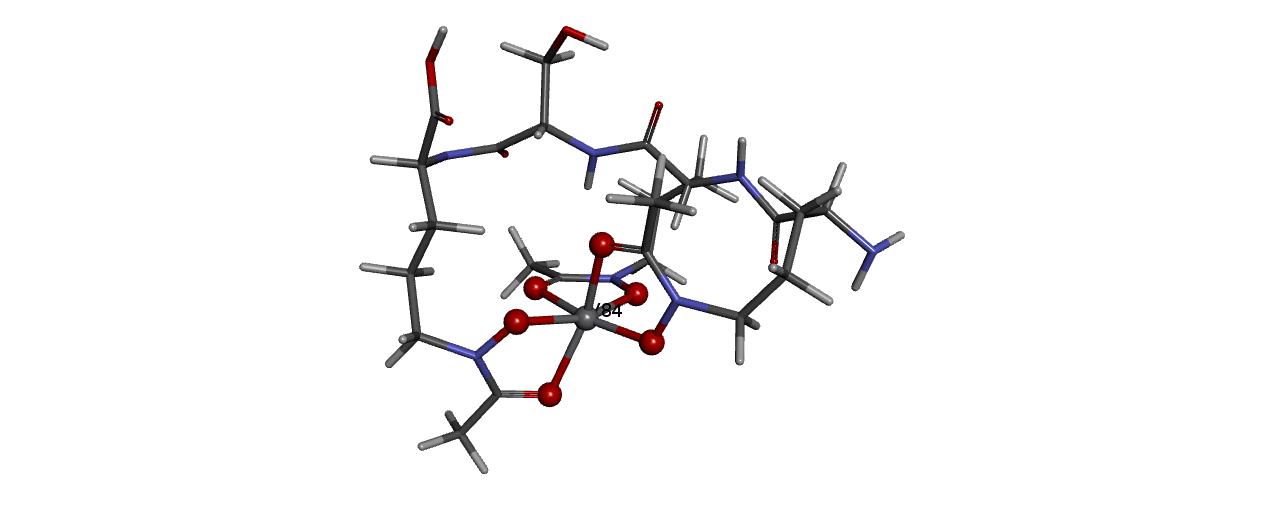

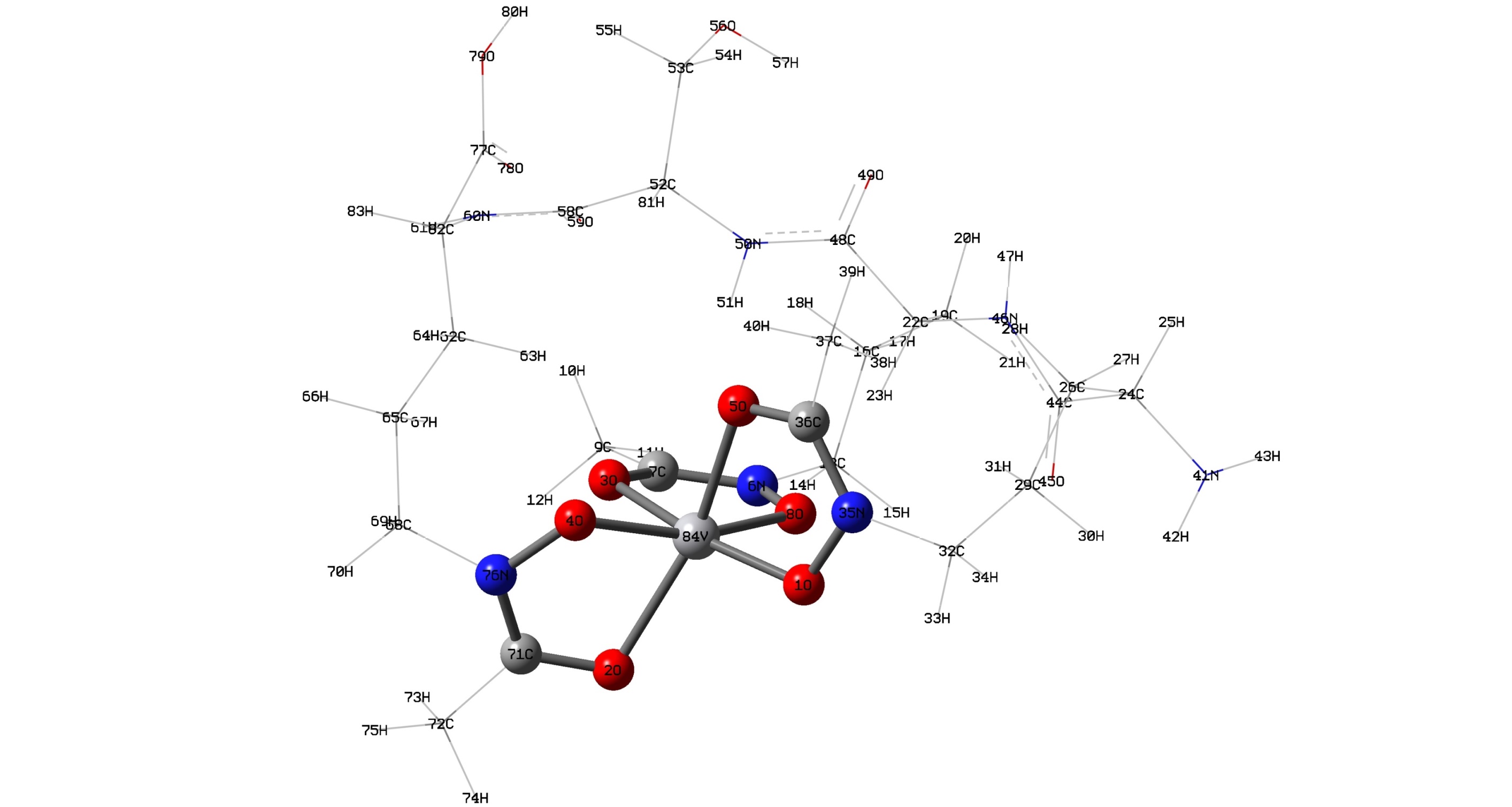

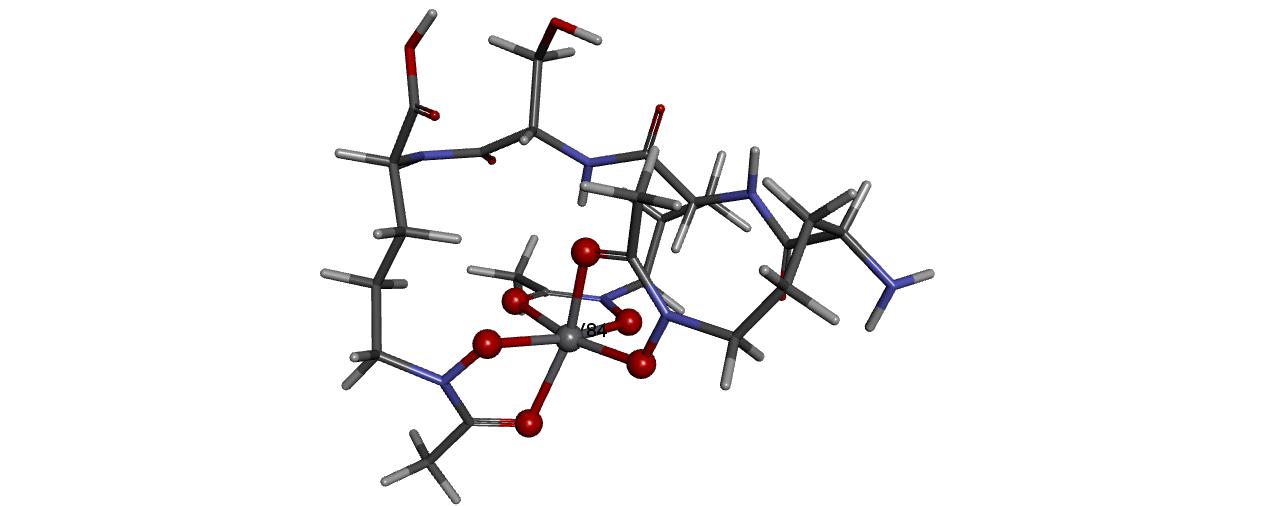


**B3LYP/DEF2TZVP**

**M06/DEF2TZVP**

**Table S1**: The optimized bond lengths in Å at B3LYP/M06/def2tzvp level

13 Fe (III) - amphibactin


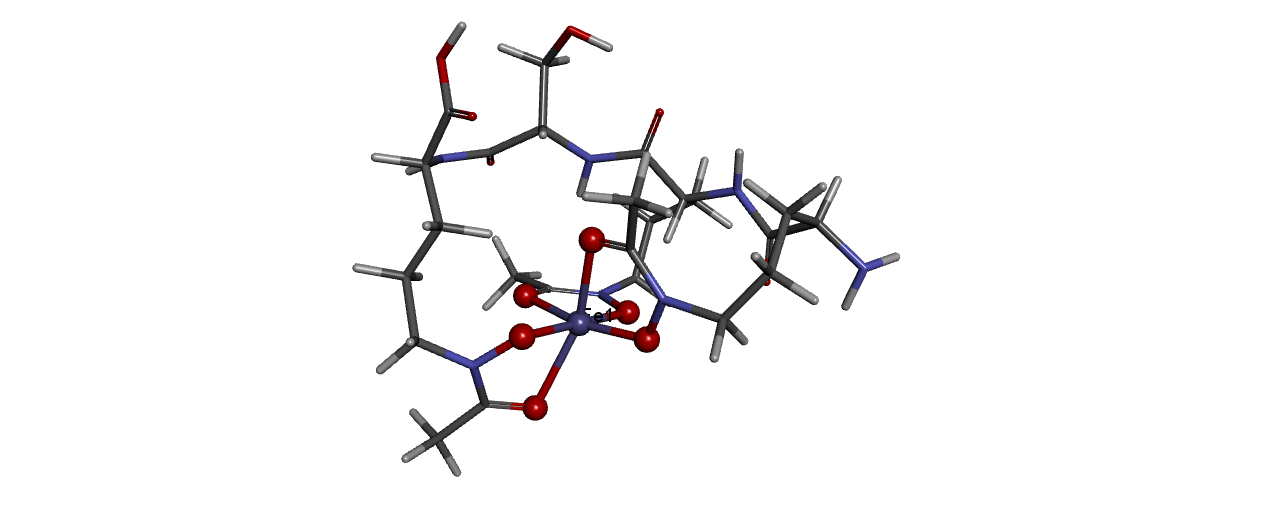

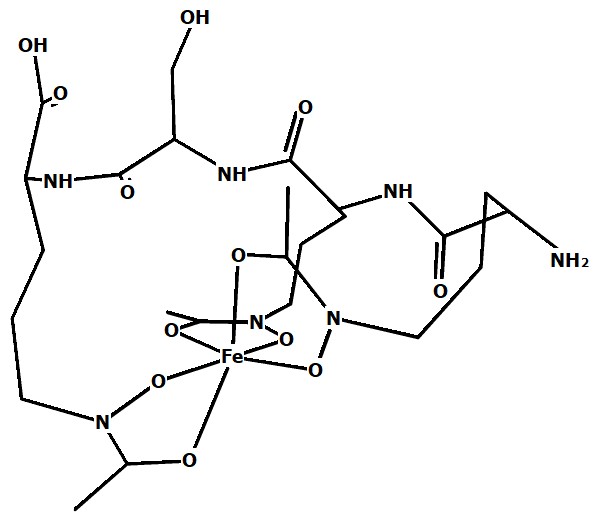

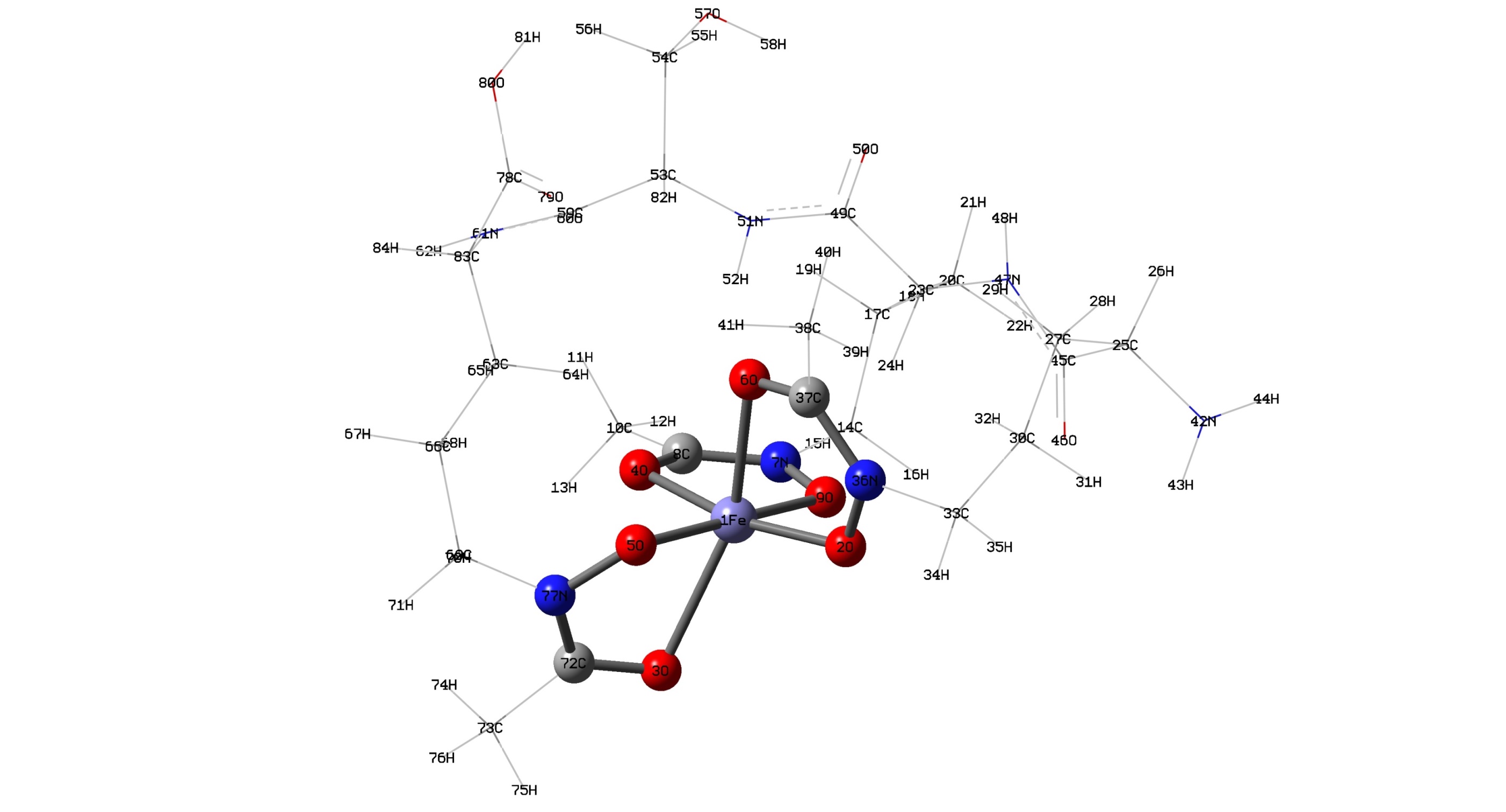


**B3LYP/DEF2TZVP**


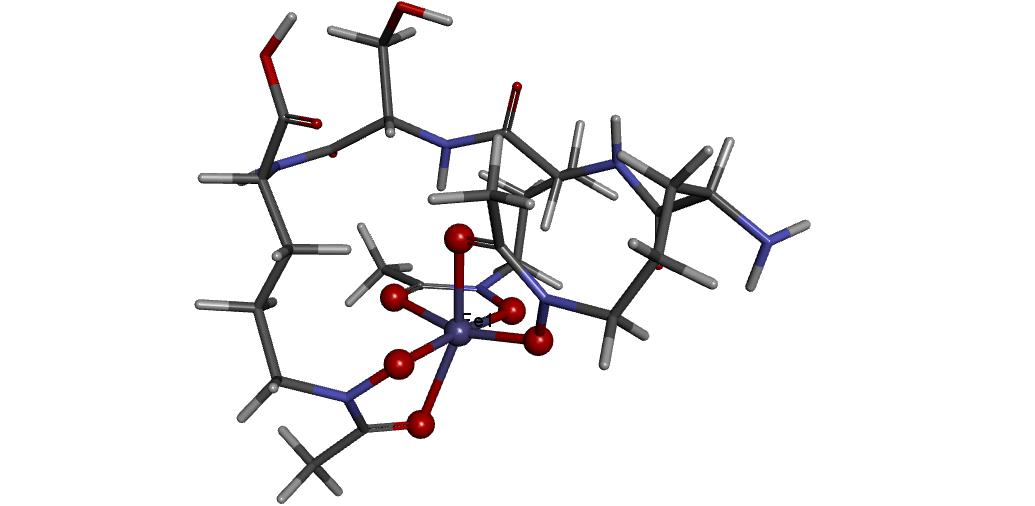


**M06/DEF2TZVP**

| **Metal** | **Bond Lengths (Å)** | | | | | | **Average** |
| --- | --- | --- | --- | --- | --- | --- | --- |
| **Ga B3LYP** | 1.933 | 2.055 | 1.944 | 2.039 | 1.948 | 2.155 | 2.012 |
| **M06** | 1.924 | 2.049 | 1.909 | 2.015 | 1.924 | 2.142 | 1.994 |
| **Co B3LYP** | 1.967 | 1.876 | 1.879 | 1.907 | 1.905 | 1.970 | 1.917 |
| **M06** | 1.949 | 1.861 | 1.883 | 1.893 | 1.869 | 1.956 | 1.914 |
| **Cr B3LYP** | 2.056 | 1.982 | 1.942 | 2.067 | 1.949 | 2.000 | 1.999 |
| **M06** | 2.054 | 1.962 | 1.929 | 2.037 | 1.942 | 1.979 | 1.984 |
| **Fe II B3LYP** | 2.027 | 3.199 | 1.901 | 1.984 | 2.361 | 1.944 | - |
| **M06** | 2.032 | 2.749 | 1.935 | 1.961 | 2.226 | 1.937 | - |
| **Fe III B3LYP** | 1.888 | 2.298 | 1.856 | 2.205 | 1.901 | 1.992 | 2.023 |
| **M06** | 1.883 | 2.222 | 1.860 | 2.164 | 1.882 | 1.999 | 2.002 |
| **Mn B3LYP** | 1.892 | 1.967 | 1.924 | 2.054 | 2.042 | 1.896 | 1.963 |
| **M06** | 1.883 | 1.941 | 1.912 | 2.037 | 2.027 | 1.889 | 1.948 |
| **Mo B3LYP** | 1.668 | 1.998 | 2.251 | 2.055 | 2.272 | 1.970 | - |
| **M06** | 1.648 | 1.995 | 2.195 | 2.039 | 2.322 | 1.976 | - |
| **Os B3LYP** | 2.010 | 2.092 | 2.058 | 2.135 | 2.135 | 1.983 | 2.069 |
| **M06** | 2.024 | 2.075 | 2.037 | 2.129 | 2.140 | 1.992 | 2.066 |
| **Ru B3LYP** | 2.123 | 2.043 | 2.072 | 1.982 | 1.959 | 2.128 | 2.051 |
| **M06** | 2.117 | 2.049 | 2.007 | 1.968 | 2.003 | 2.125 | 2.045 |
| **Sc B3LYP** | 2.174 | 2.071 | 2.085 | 2.262 | 2.049 | 2.156 | 2.133 |
| **M06** | 2.185 | 2.068 | 2.059 | 2.241 | 2.028 | 2.154 | 2.123 |
| **Ti B3LYP** | 2.127 | 1.986 | 2.096 | 1.999 | 2.226 | 2.000 | 2.072 |
| **M06** | 2.135 | 1.976 | 2.077 | 1.969 | 2.207 | 1.978 | 2.057 |
| **V B3LYP** | 1.918 | 2.086 | 2.016 | 2.155 | 1.918 | 2.127 | 2.037 |
| **M06** | 1.900 | 2.048 | 2.014 | 2.129 | 1.912 | 2.114 | 2.020 |

**Table S2**: Bite angle in degrees measured for different amphibactin-metal complexes at B3LYP/def2tzvp level. In brackets bite angle calculated at M06/def2tzvp level. The values in bold are average values of bite angle.

| **Bite angle** | **Amphi-Fe^3+^** | **Bite angle** | **Amphi-Fe^2+^** | **Bite angle** | **Amphi-Cr^3+^** |
| --- | --- | --- | --- | --- | --- |
| O6-Fe1-O2 | 77.7 **77.4** (77.74) (**78.3)** | O6-Fe1-O2 | 73.7 **70.4** (77.0) (**74.9)** | O5-Cr84-O1 | 79.4 **78.5** (80.1) (**79.3)** |
| O5-Fe1-O3 | 74.2 (75.97) | O5-Fe1-O3 | 58.1 (66.8) | O4-Cr84-O2 | 78.2 (78.9) |
| O4-Fe1-O9 | 80.2 (81.05) | O4-Fe1-O9 | 79.4 (81.1) | O8-Cr84-O3 | 77.9 (78.8) |
| **Bite angle** | **Amphi-Ru^3+^** | **Bite angle** | **Amphi-Os^3+^** | **Bite angle** | **Amphi-Mo^3+^** |
| O5-Ru84-O1 | 79.3 **78.3** (79.2) **78.4** | O5-Os84-O1 | 78.0 **77.5** (77.7) (**77.5)** | O5-Mo84-O1 | 102.2 (106.1) |
| O4-Ru84-O2 | 78.4 (78.1) | O4-Os84-O2 | 77.7 77.1 | O4-Mo84-O2 | 74.5 (75.5) |
| O8-Ru84-O3 | 77.1 (78.0) | O8-Os84-O3 | 76.8 77.6 | O8-Mo84-O3 | 70.9 (70.7) |
| **Bite angle** | **Amphi-Ga^3+^** | **Bite angle** | **Amphi-Co^3+^** | **Bite angle** | **Amphi-Sc^3+^** |
| O1-Ga84-O5 | 80.5 **78.6** (81.3) **79.2** | O5-Co84-O1 | 84.1 **82.9** (84.8) **83.6** | O5-Sc84-O1 | 72.9 **72.1 (**73.3) (**72.7)** |
| O2-Ga84-O4 | 78.2 (78.5) | O4-Co84-O2 | 81.8 (82.3) | O8-Sc84-O3 | 70.4 71.6 |
| O8-Ga84-O3 | 77.0 (77.9) | O8-Co84-O3 | 82.8 (83.7) | O4-Sc84-O2 | 73.0 73.3 |
| **Bite angle** | **Amphi-Ti^3+^** | **Bite angle** | **Amphi-V^3+^** | **Bite angle** | **Amphi-Mn^3+^** |
| O5-Ti84-O1 | 75.4 **73.9 (**76.0) (**74.6)** | O5-V84-O1 | 77.7 **76.2** (78.3) (**76.4)** | O5-Mn84-O1 | 81.4 **79.8** (81.9) (**80.5)** |
| O8-Ti84-O3 | 72.2 73.1 | O4-V84-O2 | 77.2 (77.2) | O4-Mn84-O2 | 79.1 (79.5) |
| O4-Ti84-O2 | 74.3 74.7 | O8-V84-O3 | 73.8 (74.7) | O8-Mn84-O3 | 79.0 (80.0) |

**ES1 Equations:** Equations to find Electrode potential from thermodynamic parameters, electrode potential and free energy change at p^H^=7.

The free energy change for the reduction reaction of Fe(III)amphibactin+ ē→Fe(II)amphibactin is

${\Delta G}_{s}^{0}={\Delta G}_{gas}^{0}+{\Delta G}_{sol}^{0}=-76.73+\left( -35.62 \right)=-112.35 kcal/mol$

$$E_{{Fe}^{3+}Red}^{0}\left( V \right)=-\frac{{\Delta G}_{s}^{0}}{nF}=4.88 V\longrightarrow(S1)=4.77V$$

$$E^{0}\left( V | RE \right)=E_{{Fe}^{3+}Red}^{0}\left( V \right)-E_{RE}^{0}\left( V \right)=4.78-4.44=0.44 V\longrightarrow(S2)$$

^*^${Absolute electrode potentail of SHE E}_{RE}^{0}\left( V \right)=4.44 V$

**IUPAC. Compendium of Chemical Terminology, 2nd ed. (the "Gold Book"). Compiled by A. D. McNaught and A. Wilkinson. Blackwell Scientific Publications, Oxford (1997).*

$$E^{0}\left( V | RE \right)>E_{RE}^{0}(V)\longrightarrow(S3)$$

$$E_{Cell}^{0}=E_{Cathode}^{0}-E_{Anode}^{0}=0.44-0.32=0.12 V\longrightarrow(S4)$$

$$bB+mH^{+}+nē\rightleftharpoons aA+wH_{2}O\longrightarrow(S5)$$

Where A= reactant

B=Product

n= number of electrons released

a=moles of reactant

w=moles of water

b=moles of product

m=moles of hydrogen ions

$$E_{h}=E_{Cell}^{0}-\frac{0.0592}{2}ln\frac{\left[ A \right]^{a}\left[ H_{2}O \right]^{w}}{\left[ B \right]^{b}\left[ H^{+} \right]^{m}}\longrightarrow(S6)$$

At 25^0^ C and 1 atm. pressure, activity of water is unity.

$$E_{h}=E_{Cell}^{0}-2.303\frac{RT}{NF}log\frac{\left[ A \right]^{a}}{\left[ B \right]^{b}}+2.303\frac{RTm}{nF}p^{H}\longrightarrow(S7)$$

$$E_{h}=E_{Cell}^{0}-\frac{0.0592}{2}log\frac{\left[ A \right]^{a}}{\left[ B \right]^{b}}-0.0592\frac{m}{n}p^{H}\longrightarrow(S8)$$

The standard potential for this cell (at 298 K, [Fe^3+^] = 1.0 M, [H^+^] = 1.0 M and P_H2_ = 1.0 atm, activity of water unity,

$$E_{h}(at p^{H}=7)=E_{Cell}^{0}-0.05916*p^{H}=(0.44-0.05916*7)V\longrightarrow(S9)$$

$$E_{h} {Fe}^{3+}{\to Fe}^{2+}at p^{H}7=0.02588 V\longrightarrow(S10)$$

$${\Delta G}_{h{Fe}^{3+}{\to Fe}^{2+}}=-nFE_{h{Fe}^{3+}{\to Fe}^{2+}}=-1*23.061*0.02588=-0.60 kcal/mol\longrightarrow(S11)$$

At acidic pH, pH=2 $E_{h} {Fe}^{3+}{\to Fe}^{2+}at p^{H}2=0.32168 V=0.32 V \longrightarrow(S12)$

$${\Delta G}_{h{Fe}^{3+}{\to Fe}^{2+}}at p^{H}2=-7.42 kcal/mol\longrightarrow(S13)$$

The standard potential for this cell (at 298 K, [NADH] = 1.0 M, [H^+^] = 1.0 M and P_H2_ = 1.0 atm, activity of water unity,

$$E_{h}(at p^{H}=7)=E_{Cell}^{0}-0.05916*p^{H}=(0.32-0.05916*7)V\longrightarrow(S14)$$

$$E_{h}\left( NADH \right)at p^{H}7=-0.09412 V\longrightarrow(S15)$$

$$E_{Cell}^{0} at p^{H}7 =E_{Cathode}^{0}-E_{Anode}^{0}= 0.02588-(-0.09412)=0.12 V\longrightarrow(S16)$$

With FADH2(free) as reducing agent,

$$E_{Cell}^{0}=E_{Cathode}^{0}-E_{Anode}^{0}=0.44-0.22=0.22\longrightarrow(S17)$$

At pH=7,

$$E_{h}\left( FADH2 \right)at p^{H}7=-0.19412 V\longrightarrow(S18)$$

$$E_{Cell}^{0} at p^{H}7 =E_{Cathode}^{0}-E_{Anode}^{0}= 0.02588-(-0.19412)=0.22 V\longrightarrow(S19)$$

With FADH_2_ (bound) as reducing agent,

$$E_{Cell}^{0}=E_{Cathode}^{0}-E_{Anode}^{0}=0.44-0.031=0.409 V=0.41 V\longrightarrow(S20)$$

$$E_{Cell}^{0} at p^{H}7 =E_{Cathode}^{0}-E_{Anode}^{0}= 0.02588-\left( -0.38312 \right)=0.409 V=0.41 V\longrightarrow(S21)$$

$${\Delta G}_{h}={\Delta G}^{0}-2.303RTlog\frac{\left[ A \right]^{a}}{\left[ B \right]^{b}}+2.303mRTp^{H}\longrightarrow(S22)$$

$${\Delta G}_{h}={\Delta G}^{0}+2.303mRTp^{H}\longrightarrow(S23)$$

Add $2.303mRTp^{H}=$28.62 to all the $G$ terms in equation of ${\Delta G}_{t}^{\#}$

1. Karp, Gerald, Cell and Molecular Biology, 5th Ed., Wiley, 2008
2. Campbell, M. & Farrell, S. *Biochemistry*. (Cengage Learning, 2005).
3. Ochiai, Ei-Ichiro. *General principles of biochemistry of the elements*. Vol. 7. Springer Science & Business Media, 2012.
4. Fetter, Charles Willard, Thomas Boving, and David Kreamer. *Contaminant hydrogeology*. Waveland Press, 2017.
5. Burgot, Jean-Louis. *Thermodynamics in Bioenergetics*. CRC Press, 2019.
6. Champe, Pamela C., Richard A. Harvey, and Denise R. Ferrier. *Biochemistry*. Lippincott Williams & Wilkins, 2005.
7. Garrett, R. H. & Grisham, C. M. *Biochemistry*. (Cengage Learning, 2008).
8. Chang, Raymond. *Physical Chemistry for the Biosciences*. Sansalito, CA: University Sciences, 2005.
9. Atkins, Peter and de Paula, Julio. *Physical Chemistry for the Life Sciences*. New York, NY: W. H. Freeman and Company, 2006. Page 153-163, 286.
10. Stryer, Lubert. *Biochemistry (Third Edition)*. New York, NY: W.H. Freeman and Company, 1988. Page 181-184.

Computationally from G09 the pKa for hydroxamate CO can be calculated from the equation as follows:

Here a proton (H^+^) is attached to one of the O of CO of hydroxamate group of the three hydrogen protonated at three O atoms of NO of hydroxamate group Fe(II)-amphibactin-3H complex to get Fe(II)-amphibactin-4H complex. From the following reaction and equation given below we can find the p^ka^ of CO group for protonation.

Fe(II)-amphibactin-3H + H^+^ 🡪 Fe(II)-amphibactin-4H → S24

∆G°= -2349.233942 - ( -2348.891572+ -0.010017)= -0.332353=-872.592868kJ/mol → S25

p^Kca^=p^Kfa^-∆G°aq/RTln10 =8-(-

1. Jensen, Jan H. "Predicting accurate absolute binding energies in aqueous solution: thermodynamic considerations for electronic structure methods." *Physical Chemistry Chemical Physics* 17.19 (2015): 12441-12451.

**Optimized geometry coordinates of different amphibactin-metal complexes at B3LYP/def2tzvp level.**

Amphi-Ga

O -1.54062 -2.30122 -1.15312

O 0.90403 -3.79657 0.06259

O 1.62683 -1.32896 1.18346

O 1.48968 -1.99914 -1.61057

O -0.22506 -0.09863 -1.08174

N -0.15846 -1.37085 2.51455

C 1.15142 -1.23958 2.35469

O -0.89549 -1.69233 1.41205

C 2.07165 -1.02991 3.52015

H 2.58348 -0.07377 3.40943

H 1.56964 -1.05754 4.48295

H 2.82355 -1.82084 3.49376

C -0.96478 -1.02495 3.68616

H -0.47042 -1.41882 4.57328

H -1.90042 -1.56747 3.55921

C -1.21939 0.48404 3.88053

H -1.54456 0.60641 4.91834

H -0.27041 1.02302 3.8145

C -2.30121 1.14337 3.00747

H -2.44332 2.17369 3.34629

H -3.24208 0.62009 3.18629

C -2.1164 1.12853 1.47089

H -1.79233 0.12569 1.18901

C -5.30281 0.73743 -0.59471

H -5.70203 1.72638 -0.35083

C -4.65734 0.88348 -1.9958

H -5.37845 1.4324 -2.60688

H -3.78483 1.52939 -1.90895

C -4.32565 -0.38799 -2.79548

H -5.26812 -0.91721 -2.96025

H -3.99537 -0.07649 -3.7892

C -3.39938 -1.47847 -2.24822

H -3.3449 -2.28177 -2.98676

H -3.8364 -1.913 -1.3488

N -2.01188 -1.20741 -1.83315

C -1.2516 -0.12501 -1.83801

C -1.43249 1.02953 -2.77591

H -2.24979 0.91017 -3.47589

H -1.54713 1.95677 -2.21456

H -0.50088 1.1106 -3.33978

N -6.40846 -0.21307 -0.6077

H -6.06665 -1.10318 -0.25397

H -7.1289 0.07916 0.04122

C -4.25449 0.39798 0.48639

O -4.22239 -0.70112 1.01761

N -3.37428 1.38924 0.78235

H -3.39259 2.25463 0.26105

C -1.09367 2.13195 0.90838

O -1.45924 3.0643 0.18487

N 0.19521 1.92004 1.21924

H 0.42332 1.09804 1.75143

C 1.31832 2.57085 0.5426

C 1.32527 4.11884 0.72262

H 0.88842 4.34293 1.70147

H 2.35307 4.47649 0.72352

O 0.66752 4.79216 -0.32444

H -0.23388 4.42495 -0.35529

C 2.60586 2.01808 1.14897

O 2.68748 1.88524 2.36403

N 3.69273 1.77573 0.36396

H 4.48319 1.50609 0.93089

C 3.41035 0.20318 -1.59921

H 2.33865 0.10173 -1.45504

H 3.57112 0.19665 -2.67931

C 4.14533 -0.98429 -0.96251

H 5.22659 -0.85469 -1.09655

H 3.95154 -1.0176 0.11094

C 3.80171 -2.36642 -1.55962

H 3.84619 -2.32497 -2.65127

H 4.53164 -3.09839 -1.21938

C 2.10983 -3.78043 -0.33677

C 3.07424 -4.80453 0.18186

H 3.47558 -4.48283 1.14605

H 2.52251 -5.72882 0.3453

H 3.90214 -4.99933 -0.49609

N 2.47392 -2.84112 -1.19869

C 3.29778 2.70983 -1.93222

O 2.69902 2.55718 -2.96197

O 3.5921 3.92031 -1.42202

H 3.12995 4.58403 -1.96042

H 1.2486 2.35552 -0.52488

C 3.87042 1.57113 -1.07854

H 4.95359 1.65878 -1.22636

Ga 0.11744 -1.92175 -0.23538

Amphi-Co

O -1.33605 -2.3684 -1.13587

O 0.80822 -3.68036 0.28732

O 1.561 -1.29863 1.13721

O 1.3542 -2.08 -1.58085

O -0.16948 -0.13836 -0.85774

N -0.22528 -1.3138 2.47587

C 1.07766 -1.19733 2.31792

O -0.96677 -1.66518 1.38082

C 2.01228 -0.9935 3.47102

H 2.594 -0.0856 3.31404

H 1.50415 -0.92795 4.42894

H 2.70026 -1.84132 3.497

C -1.02973 -0.98522 3.65495

H -0.53554 -1.39452 4.53551

H -1.96428 -1.52708 3.51609

C -1.28571 0.52011 3.86869

H -1.62697 0.62805 4.90282

H -0.33593 1.05962 3.82202

C -2.35117 1.18762 2.98203

H -2.49878 2.21651 3.32262

H -3.29591 0.66395 3.13733

C -2.1222 1.17177 1.45219

H -1.77465 0.17254 1.18494

C -5.20462 0.68229 -0.7452

H -5.62818 1.67239 -0.55219

C -4.49389 0.79536 -2.11749

H -5.19301 1.31428 -2.77842

H -3.63849 1.4596 -2.00827

C -4.10519 -0.49051 -2.86981

H -5.03277 -1.03052 -3.07757

H -3.72068 -0.19358 -3.84876

C -3.20071 -1.56584 -2.2584

H -3.11459 -2.38536 -2.97503

H -3.66687 -1.97933 -1.36284

N -1.83065 -1.27315 -1.8072

C -1.12369 -0.17004 -1.71728

C -1.23401 1.00312 -2.6411

H -1.98467 0.87956 -3.41221

H -1.41297 1.92095 -2.08178

H -0.25851 1.10502 -3.12215

N -6.2958 -0.28413 -0.78321

H -5.95756 -1.16017 -0.39268

H -7.04806 0.01309 -0.17376

C -4.20501 0.3887 0.3941

O -4.17661 -0.70026 0.94534

N -3.36006 1.40379 0.71578

H -3.37949 2.26522 0.18821

C -1.09502 2.18629 0.91996

O -1.45154 3.12707 0.20291

N 0.19161 1.9692 1.24342

H 0.40353 1.11859 1.73635

C 1.31959 2.58289 0.5375

C 1.37636 4.13 0.70326

H 0.97745 4.37554 1.69292

H 2.41365 4.45754 0.6675

O 0.70487 4.81264 -0.32979

H -0.20516 4.46546 -0.33518

C 2.60624 1.99583 1.11351

O 2.72459 1.88923 2.32788

N 3.6576 1.69093 0.30069

H 4.45398 1.40505 0.85124

C 3.31488 0.08264 -1.62651

H 2.25081 -0.01449 -1.43577

H 3.43183 0.0633 -2.71228

C 4.06849 -1.10479 -1.01094

H 5.14269 -0.99694 -1.20696

H 3.93457 -1.11751 0.07229

C 3.66682 -2.49152 -1.56442

H 3.67106 -2.47574 -2.65749

H 4.38918 -3.23683 -1.23471

C 1.97753 -3.77843 -0.20449

C 2.89208 -4.85543 0.29449

H 3.34676 -4.5493 1.23968

H 2.28676 -5.73921 0.49248

H 3.67822 -5.11179 -0.41208

N 2.34118 -2.92031 -1.14364

C 3.19091 2.58217 -1.9945

O 2.51898 2.41389 -2.97616

O 3.54333 3.7998 -1.54231

H 3.05511 4.45887 -2.06337

H 1.2182 2.36225 -0.52589

C 3.79395 1.45777 -1.14264

H 4.87206 1.54408 -1.32463

Co 0.17057 -1.86878 -0.1367

Amphi-Cr

O -1.421985000 -2.370649000 -1.170067000

O 1.014374000 -3.797601000 0.158762000

O 1.600204000 -1.342092000 1.212109000

O 1.497301000 -2.062166000 -1.614812000

O -0.226778000 -0.161583000 -0.980049000

N -0.175096000 -1.366180000 2.558108000

C 1.129750000 -1.234737000 2.393280000

O -0.905273000 -1.725817000 1.463588000

C 2.064704000 -1.004690000 3.541317000

H 2.580935000 -0.054173000 3.405885000

H 1.570803000 -1.008396000 4.508851000

H 2.811275000 -1.801149000 3.525237000

C -0.979873000 -1.014366000 3.729745000

H -0.468118000 -1.374937000 4.621221000

H -1.901677000 -1.584143000 3.621254000

C -1.272166000 0.491109000 3.889664000

H -1.608789000 0.626965000 4.922079000

H -0.336265000 1.051489000 3.817343000

C -2.361610000 1.103282000 2.992411000

H -2.543770000 2.132183000 3.315964000

H -3.287748000 0.550592000 3.159826000

C -2.144819000 1.077504000 1.460378000

H -1.792521000 0.079339000 1.195858000

C -5.275800000 0.600706000 -0.676960000

H -5.700563000 1.582453000 -0.447465000

C -4.607170000 0.751374000 -2.066513000

H -5.327641000 1.281318000 -2.694931000

H -3.750817000 1.416839000 -1.967425000

C -4.234094000 -0.517063000 -2.854189000

H -5.163654000 -1.060028000 -3.044814000

H -3.879963000 -0.201703000 -3.838603000

C -3.306243000 -1.593231000 -2.281988000

H -3.225762000 -2.399675000 -3.014183000

H -3.751417000 -2.027045000 -1.385792000

N -1.931108000 -1.293843000 -1.848172000

C -1.210439000 -0.190919000 -1.798373000

C -1.363294000 0.975087000 -2.725446000

H -2.145524000 0.848831000 -3.463686000

H -1.521263000 1.893328000 -2.159947000

H -0.408275000 1.079233000 -3.245162000

N -6.360304000 -0.373688000 -0.704773000

H -6.007090000 -1.252858000 -0.335062000

H -7.100937000 -0.091179000 -0.074544000

C -4.241009000 0.289165000 0.426185000

O -4.192832000 -0.808926000 0.957655000

N -3.392809000 1.302597000 0.740738000

H -3.428952000 2.171640000 0.226739000

C -1.134282000 2.101779000 0.914869000

O -1.510312000 3.041697000 0.206644000

N 0.157824000 1.900617000 1.224099000

H 0.392535000 1.056171000 1.717432000

C 1.269296000 2.562218000 0.537756000

C 1.267829000 4.109278000 0.723430000

H 0.831395000 4.328637000 1.703234000

H 2.293384000 4.473302000 0.722636000

O 0.603427000 4.782062000 -0.320632000

H -0.297844000 4.414115000 -0.344778000

C 2.569749000 2.016361000 1.122977000

O 2.675750000 1.893244000 2.336781000

N 3.641857000 1.768873000 0.318520000

H 4.443628000 1.505037000 0.872280000

C 3.369789000 0.189374000 -1.640609000

H 2.305781000 0.054606000 -1.469645000

H 3.503688000 0.190505000 -2.724400000

C 4.155632000 -0.978390000 -1.028413000

H 5.228309000 -0.822069000 -1.197781000

H 3.999848000 -1.015177000 0.051824000

C 3.823025000 -2.370115000 -1.611807000

H 3.825754000 -2.331624000 -2.704335000

H 4.580433000 -3.085284000 -1.295718000

C 2.198534000 -3.788093000 -0.302317000

C 3.183439000 -4.812190000 0.176300000

H 3.591913000 -4.509788000 1.143533000

H 2.642983000 -5.745990000 0.325886000

H 4.003896000 -4.981781000 -0.517135000

N 2.519321000 -2.867159000 -1.199646000

C 3.171664000 2.690904000 -1.966019000

O 2.534128000 2.519330000 -2.969522000

O 3.460172000 3.909487000 -1.473676000

H 2.952650000 4.560836000 -1.986033000

H 1.186938000 2.351861000 -0.529613000

C 3.799156000 1.570124000 -1.127354000

H 4.875897000 1.691887000 -1.296353000

Cr 0.189697000 -1.943976000 -0.173583000

Amphi-FeII

Fe 0.829074000 -1.693216000 -0.010934000

O -0.752553000 -2.416413000 -0.965875000

O 2.918851000 -4.101819000 0.243205000

O 2.180651000 -1.182178000 1.410558000

O 2.038612000 -2.124508000 -1.413115000

O 0.025650000 0.054406000 -1.380083000

N 0.324369000 -1.025558000 2.633914000

C 1.646621000 -0.996071000 2.542319000

O -0.354979000 -1.250489000 1.465950000

C 2.511029000 -0.710803000 3.739854000

H 2.512418000 0.360869000 3.948950000

H 2.185408000 -1.245834000 4.632069000

H 3.524517000 -1.021764000 3.495857000

C -0.537392000 -0.848979000 3.787017000

H 0.095229000 -0.852672000 4.673334000

H -1.188237000 -1.727904000 3.833415000

C -1.390928000 0.440549000 3.809144000

H -1.645333000 0.605066000 4.861592000

H -0.759237000 1.281413000 3.516214000

C -2.728293000 0.456209000 3.040516000

H -3.307552000 1.319633000 3.383587000

H -3.304289000 -0.436191000 3.298218000

C -2.660343000 0.496108000 1.499116000

H -2.089705000 -0.373057000 1.184354000

C -5.408470000 -0.631500000 -0.821528000

H -6.034352000 0.255326000 -0.678082000

C -4.597844000 -0.372381000 -2.119126000

H -5.315359000 0.020042000 -2.845304000

H -3.885671000 0.430132000 -1.926708000

C -3.900391000 -1.560458000 -2.807073000

H -4.645857000 -2.351694000 -2.935227000

H -3.656757000 -1.245332000 -3.823701000

C -2.697133000 -2.270887000 -2.169454000

H -2.406843000 -3.091002000 -2.834888000

H -3.009425000 -2.736974000 -1.234642000

N -1.456083000 -1.581949000 -1.785323000

C -0.969642000 -0.367567000 -2.025787000

C -1.504880000 0.527357000 -3.118991000

H -2.242878000 0.071442000 -3.769104000

H -1.917045000 1.443402000 -2.689421000

H -0.644911000 0.823184000 -3.722237000

N -6.295804000 -1.782633000 -0.964767000

H -5.759871000 -2.614732000 -0.729515000

H -7.020655000 -1.733760000 -0.256678000

C -4.474976000 -0.744801000 0.397399000

O -4.189480000 -1.830484000 0.876087000

N -3.970034000 0.432032000 0.855707000

H -4.245697000 1.293121000 0.404412000

C -1.976198000 1.770919000 0.989024000

O -2.656427000 2.771253000 0.711167000

N -0.645893000 1.678338000 0.911113000

H -0.303713000 0.717679000 0.943726000

C 0.295562000 2.669053000 0.393180000

C -0.068849000 4.133569000 0.747020000

H -0.416676000 4.157804000 1.785162000

H 0.837947000 4.731683000 0.682468000

O -1.000009000 4.713400000 -0.137551000

H -1.815055000 4.185856000 -0.014763000

C 1.661637000 2.400002000 1.032891000

O 1.746441000 2.234131000 2.242428000

N 2.800539000 2.479202000 0.281895000

H 3.607241000 2.320211000 0.867324000

C 3.079323000 0.967761000 -1.731011000

H 2.094271000 0.516892000 -1.620518000

H 3.262075000 1.054645000 -2.804966000

C 4.158239000 0.083146000 -1.102371000

H 5.137405000 0.562656000 -1.245584000

H 3.996134000 -0.013048000 -0.026693000

C 4.240795000 -1.341331000 -1.695813000

H 3.994941000 -1.306826000 -2.763473000

H 5.265171000 -1.699467000 -1.611742000

C 3.722033000 -3.323676000 -0.267497000

C 5.216270000 -3.482480000 0.028450000

H 5.651983000 -2.585332000 0.473907000

H 5.306442000 -4.303909000 0.735485000

H 5.792546000 -3.728560000 -0.867339000

N 3.359169000 -2.308748000 -1.082590000

C 2.288491000 3.375119000 -2.013293000

O 1.890721000 3.161179000 -3.126392000

O 2.155670000 4.582735000 -1.417169000

H 1.594426000 5.122039000 -1.996752000

H 0.337269000 2.566118000 -0.691981000

C 3.081809000 2.389182000 -1.155532000

H 4.105944000 2.779712000 -1.240538000

Amphi-FeIII

Fe 0.217315000 -1.913764000 -0.108339000

O -1.394699000 -2.165033000 -0.994216000

O 1.274954000 -3.945161000 0.075925000

O 1.637233000 -1.418143000 1.198304000

O 1.352171000 -2.039889000 -1.612413000

O -0.154384000 0.075475000 -0.983865000

N -0.081997000 -1.515373000 2.593286000

C 1.215359000 -1.365910000 2.399581000

O -0.818527000 -1.838645000 1.483878000

C 2.187381000 -1.161833000 3.517870000

H 2.677365000 -0.195511000 3.395397000

H 1.725288000 -1.209655000 4.500065000

H 2.946555000 -1.942565000 3.446388000

C -0.887615000 -1.212339000 3.775133000

H -0.335085000 -1.542674000 4.653947000

H -1.775243000 -1.837986000 3.685965000

C -1.270646000 0.271157000 3.950377000

H -1.609207000 0.367786000 4.986527000

H -0.370100000 0.887706000 3.887034000

C -2.401460000 0.836726000 3.071968000

H -2.666284000 1.828650000 3.449133000

H -3.282067000 0.204845000 3.201788000

C -2.182518000 0.919505000 1.542471000

H -1.786984000 -0.041373000 1.214311000

C -5.208045000 0.466735000 -0.727055000

H -5.678936000 1.415366000 -0.453096000

C -4.475253000 0.735411000 -2.068362000

H -5.164872000 1.322150000 -2.680566000

H -3.618518000 1.382069000 -1.877338000

C -4.071152000 -0.463610000 -2.940996000

H -4.981281000 -1.025643000 -3.166752000

H -3.732682000 -0.067304000 -3.900289000

C -3.092953000 -1.531491000 -2.435908000

H -2.906451000 -2.240539000 -3.247137000

H -3.560403000 -2.093907000 -1.628351000

N -1.791959000 -1.164599000 -1.852002000

C -1.062494000 -0.050638000 -1.847431000

C -1.210209000 1.021042000 -2.889030000

H -1.945918000 0.803161000 -3.654276000

H -1.443303000 1.971443000 -2.408155000

H -0.233029000 1.136456000 -3.360914000

N -6.253852000 -0.537950000 -0.877411000

H -5.884620000 -1.430099000 -0.557598000

H -7.030628000 -0.328660000 -0.262286000

C -4.218384000 0.114841000 0.403556000

O -4.146165000 -1.015366000 0.857096000

N -3.436879000 1.141442000 0.833577000

H -3.482657000 2.032171000 0.357691000

C -1.217329000 2.017352000 1.060877000

O -1.627979000 2.964936000 0.384424000

N 0.079679000 1.875899000 1.383237000

H 0.361501000 1.038489000 1.862718000

C 1.146948000 2.597824000 0.691054000

C 1.123436000 4.130949000 0.965664000

H 0.713928000 4.286004000 1.969320000

H 2.139765000 4.519750000 0.952415000

O 0.407976000 4.844246000 -0.017326000

H -0.474179000 4.433523000 -0.059291000

C 2.478498000 2.034023000 1.174743000

O 2.639512000 1.801524000 2.367049000

N 3.516045000 1.887807000 0.304034000

H 4.348842000 1.590766000 0.791327000

C 3.161986000 0.415813000 -1.720545000

H 2.115084000 0.236919000 -1.486930000

H 3.226533000 0.480809000 -2.808899000

C 4.030310000 -0.747219000 -1.227961000

H 5.081284000 -0.537253000 -1.463456000

H 3.956572000 -0.847396000 -0.142858000

C 3.700463000 -2.113394000 -1.863435000

H 3.580472000 -2.004175000 -2.945334000

H 4.527422000 -2.800059000 -1.690522000

C 2.372510000 -3.758312000 -0.484525000

C 3.554269000 -4.651901000 -0.201727000

H 4.249693000 -4.166117000 0.487020000

H 3.176640000 -5.552714000 0.276042000

H 4.100515000 -4.926461000 -1.103782000

N 2.496307000 -2.738071000 -1.346537000

C 2.893489000 2.930469000 -1.894509000

O 2.219061000 2.801845000 -2.880471000

O 3.171676000 4.123476000 -1.339531000

H 2.615919000 4.789488000 -1.777760000

H 1.013048000 2.444187000 -0.380279000

C 3.588003000 1.778817000 -1.158306000

H 4.650217000 1.933934000 -1.383542000

Amphi-Mn

O -1.416171000 -2.348028000 -1.130696000

O 0.895398000 -3.766759000 0.183334000

O 1.576555000 -1.350290000 1.199534000

O 1.437509000 -2.052085000 -1.568250000

O -0.200199000 -0.146107000 -1.000136000

N -0.210766000 -1.349132000 2.517147000

C 1.101546000 -1.245322000 2.371980000

O -0.924238000 -1.687010000 1.398496000

C 2.020934000 -1.041963000 3.538200000

H 2.540406000 -0.089526000 3.430709000

H 1.510967000 -1.065067000 4.497350000

H 2.765981000 -1.839311000 3.517544000

C -1.032143000 -0.993097000 3.674303000

H -0.553431000 -1.389316000 4.569342000

H -1.969542000 -1.529911000 3.534509000

C -1.278322000 0.518044000 3.859534000

H -1.614811000 0.649101000 4.892574000

H -0.325481000 1.050381000 3.798051000

C -2.344412000 1.174116000 2.966114000

H -2.486100000 2.209350000 3.289532000

H -3.290657000 0.657367000 3.134910000

C -2.127754000 1.136815000 1.434444000

H -1.793079000 0.132094000 1.173378000

C -5.270878000 0.680245000 -0.686636000

H -5.678291000 1.674366000 -0.479989000

C -4.600423000 0.786518000 -2.078968000

H -5.316932000 1.303594000 -2.722408000

H -3.739517000 1.448378000 -1.997552000

C -4.235409000 -0.506608000 -2.829790000

H -5.168494000 -1.048587000 -3.005591000

H -3.878878000 -0.221929000 -3.822732000

C -3.313449000 -1.571896000 -2.227792000

H -3.245742000 -2.401986000 -2.934396000

H -3.755997000 -1.973322000 -1.315226000

N -1.932863000 -1.270484000 -1.818420000

C -1.200847000 -0.176919000 -1.798916000

C -1.354741000 0.980403000 -2.736996000

H -2.144409000 0.851805000 -3.466907000

H -1.501425000 1.905629000 -2.179839000

H -0.403546000 1.072462000 -3.265652000

N -6.372575000 -0.274866000 -0.692445000

H -6.033340000 -1.154454000 -0.311036000

H -7.103993000 0.030839000 -0.062208000

C -4.240214000 0.375482000 0.421582000

O -4.210237000 -0.711832000 0.977041000

N -3.370734000 1.377629000 0.711709000

H -3.391885000 2.239677000 0.185335000

C -1.097034000 2.139225000 0.887072000

O -1.454052000 3.085693000 0.177958000

N 0.188974000 1.909478000 1.200757000

H 0.400793000 1.055354000 1.687763000

C 1.321823000 2.546675000 0.526186000

C 1.348255000 4.093582000 0.707622000

H 0.913861000 4.323364000 1.686055000

H 2.380670000 4.437699000 0.708626000

O 0.699646000 4.776335000 -0.339541000

H -0.208390000 4.425426000 -0.366263000

C 2.602842000 1.977394000 1.132506000

O 2.688938000 1.856508000 2.348097000

N 3.681349000 1.705777000 0.344522000

H 4.469434000 1.427597000 0.910677000

C 3.384705000 0.128157000 -1.612342000

H 2.315680000 0.027903000 -1.448714000

H 3.527990000 0.123080000 -2.694914000

C 4.127884000 -1.062778000 -0.991285000

H 5.206228000 -0.942394000 -1.153993000

H 3.962623000 -1.091669000 0.087549000

C 3.752365000 -2.443417000 -1.575104000

H 3.765900000 -2.405728000 -2.667907000

H 4.482678000 -3.184533000 -1.253944000

C 2.073266000 -3.813442000 -0.273539000

C 3.017861000 -4.882384000 0.190326000

H 3.502223000 -4.574037000 1.120035000

H 2.430239000 -5.773806000 0.403725000

H 3.784503000 -5.125785000 -0.542390000

N 2.433434000 -2.901512000 -1.169639000

C 3.279126000 2.633592000 -1.951565000

O 2.666365000 2.479989000 -2.973179000

O 3.587302000 3.844338000 -1.451030000

H 3.119274000 4.508712000 -1.983653000

H 1.250819000 2.333573000 -0.541605000

C 3.853112000 1.495598000 -1.097784000

H 4.935871000 1.579637000 -1.250825000

Mn 0.156678000 -1.897935000 -0.179331000

Amphi-Mo

O 0.000012153 0.000012622 0.000004604

O -0.000007560 -0.000001177 0.000003259

O 0.000006250 0.000007854 0.000010228

O 0.000003197 0.000000366 -0.000003695

O -0.000004043 -0.000002862 0.000001699

N -0.000000956 0.000000252 0.000006572

C 0.000002907 -0.000001726 -0.000010062

O 0.000007839 -0.000001147 -0.000004401

C 0.000000576 0.000000316 -0.000000672

H 0.000001726 0.000000744 -0.000001419

H 0.000001389 0.000000084 0.000000664

H 0.000001734 0.000001340 -0.000000675

C 0.000002594 0.000000380 -0.000002776

H 0.000002334 0.000000959 0.000001359

H 0.000001626 -0.000000968 0.000001203

C 0.000002046 -0.000001490 0.000003581

H 0.000001039 0.000000411 -0.000000475

H 0.000001290 0.000000065 -0.000000585

C -0.000002551 0.000004859 -0.000004254

H 0.000001231 -0.000000519 0.000002694

H 0.000001834 -0.000000167 0.000002823

C 0.000000964 -0.000004441 0.000004807

H -0.000001485 -0.000000298 -0.000000323

C 0.000003621 -0.000006547 -0.000005622

H -0.000000871 -0.000000891 0.000003773

C -0.000003735 0.000000488 0.000007560

H -0.000000588 -0.000001135 0.000000653

H -0.000000935 -0.000001323 0.000000811

C -0.000005931 -0.000000668 -0.000004277

H -0.000000087 -0.000001643 0.000003003

H -0.000002000 -0.000002329 0.000002006

C 0.000018961 0.000004141 0.000007768

H -0.000001769 -0.000003255 0.000002053

H -0.000001704 -0.000000586 0.000001344

N -0.000003044 -0.000014555 -0.000011166

C -0.000003270 0.000008920 0.000017599

C -0.000000312 0.000001354 -0.000000582

H 0.000000173 -0.000000150 0.000000628

H 0.000000754 -0.000003177 -0.000000122

H -0.000002588 -0.000002991 -0.000002023

N -0.000002441 -0.000000176 0.000002640

H 0.000000254 -0.000001493 0.000001754

H 0.000000118 -0.000001855 0.000003220

C -0.000006514 0.000006287 0.000009143

O 0.000002552 -0.000003078 0.000000958

N 0.000004208 -0.000002433 -0.000008949

H -0.000001245 0.000000402 0.000002289

C -0.000006439 0.000001029 -0.000007002

O -0.000000655 -0.000001263 0.000006181

N -0.000004039 -0.000007946 0.000003266

H -0.000000098 0.000002740 0.000002347

C 0.000002054 0.000003366 -0.000012042

C 0.000003860 -0.000004344 0.000008015

H 0.000001018 -0.000000481 -0.000000580

H -0.000000879 0.000003950 -0.000001195

O -0.000002912 -0.000001834 -0.000006915

H -0.000001214 0.000000419 0.000000013

C 0.000000961 0.000006992 0.000002597

O -0.000002499 -0.000001563 -0.000003808

N 0.000008959 -0.000003657 -0.000005139

H -0.000000896 0.000003946 -0.000000308

C 0.000000500 0.000001844 0.000001398

H -0.000001139 -0.000001234 -0.000002263

H -0.000001844 -0.000000859 -0.000001444

C -0.000000611 -0.000001423 -0.000001419

H -0.000001231 0.000000332 -0.000002648

H -0.000000819 0.000001852 -0.000002233

C 0.000001316 -0.000000792 -0.000001691

H -0.000001391 0.000000971 -0.000000011

H 0.000001630 0.000000935 -0.000002348

C 0.000012761 0.000007404 -0.000010740

C -0.000000696 0.000001817 -0.000000912

H 0.000000179 0.000002163 -0.000001581

H 0.000001689 -0.000000042 -0.000001384

H 0.000002607 0.000000832 0.000000099

N -0.000001233 -0.000002089 0.000008009

C -0.000002002 0.000012001 0.000007883

O -0.000000736 -0.000002365 -0.000004226

O -0.000004116 -0.000005717 -0.000006281

H -0.000000769 0.000002036 -0.000000463

H -0.000001830 0.000001710 0.000002667

C -0.000004612 0.000002925 -0.000001362

H -0.000000410 0.000000703 -0.000003348

Mo -0.000024208 -0.000013123 -0.000009745

Amphi-Os

O -0.000122065 -0.000008988 -0.000092667

O 0.000055065 0.000068155 -0.000053511

O 0.000065997 -0.000049025 0.000131494

O 0.000140221 0.000016174 -0.000095381

O -0.000034050 -0.000021142 -0.000348160

N -0.000052504 -0.000081293 -0.000057540

C 0.000064389 -0.000082143 0.000004829

O 0.000042069 0.000149077 0.000364315

C -0.000038861 0.000039888 -0.000005066

H -0.000021239 0.000005237 -0.000021473

H -0.000023669 0.000027655 -0.000023308

H -0.000001932 -0.000008058 0.000018994

C 0.000041679 0.000047350 0.000038098

H 0.000039535 -0.000021072 0.000005285

H -0.000040279 -0.000000126 -0.000092306

C -0.000075792 -0.000024721 0.000036213

H 0.000010284 0.000003784 0.000005364

H 0.000021210 -0.000046720 -0.000026340

C 0.000058389 0.000005051 -0.000032513

H -0.000000039 0.000014394 0.000006578

H -0.000009720 -0.000014218 0.000053014

C -0.000073690 0.000038283 0.000001475

H 0.000015688 -0.000002519 -0.000049169

C -0.000040908 -0.000047705 -0.000001258

H -0.000025921 -0.000004452 0.000000088

C -0.000001588 0.000186590 0.000027640

H 0.000011217 0.000000939 0.000001187

H 0.000018601 -0.000017827 0.000010711

C -0.000022884 -0.000068479 -0.000023583

H -0.000003461 0.000023236 0.000010193

H 0.000001756 -0.000033286 0.000007042

C 0.000038653 -0.000016256 -0.000023010

H -0.000015223 0.000018031 -0.000008444

H 0.000013237 -0.000029567 -0.000049549

N 0.000032893 -0.000039398 0.000011874

C 0.000020344 0.000106966 0.000067553

C -0.000036502 0.000071625 -0.000144061

H -0.000005093 -0.000034627 0.000132945

H -0.000035326 0.000002454 0.000089304

H 0.000004419 -0.000003209 -0.000005757

N -0.000023121 0.000016818 0.000046883

H 0.000000607 0.000032992 -0.000005935

H 0.000017948 0.000000637 -0.000014181

C 0.000104125 -0.000088428 0.000007003

O -0.000001439 0.000079498 0.000023784

N 0.000076644 -0.000078118 -0.000094260

H -0.000051852 -0.000037300 0.000009751

C -0.000050611 0.000134884 0.000044055

O 0.000052798 -0.000040231 0.000074293

N -0.000034171 -0.000033815 -0.000067068

H 0.000088533 -0.000225596 -0.000098856

C -0.000074746 0.000095253 -0.000028654

C -0.000046441 -0.000104548 -0.000049753

H 0.000049770 -0.000007458 0.000035984

H -0.000022195 0.000070160 0.000065545

O 0.000005507 -0.000016121 -0.000012741

H 0.000007541 0.000030700 0.000006987

C 0.000108068 -0.000041531 -0.000011462

O 0.000052240 -0.000073297 0.000012358

N -0.000059425 0.000101459 -0.000014286

H -0.000011193 0.000010722 0.000003476

C -0.000000254 0.000104978 0.000026008

H -0.000010704 0.000038844 -0.000007135

H 0.000012928 -0.000062912 0.000003996

C -0.000022590 -0.000062528 -0.000001303

H 0.000009160 0.000039724 0.000004061

H 0.000012642 -0.000015545 -0.000035803

C 0.000067484 0.000123610 0.000003460

H -0.000004119 0.000027825 -0.000003032

H 0.000004953 -0.000029578 -0.000018498

C 0.000014754 0.000013589 -0.000050589

C 0.000037929 -0.000022209 0.000037292

H -0.000040843 0.000031884 -0.000020394

H -0.000001159 0.000000597 0.000005816

H -0.000011507 0.000033940 -0.000034939

N -0.000002897 -0.000176257 0.000136628

C 0.000029081 0.000004319 -0.000024799

O -0.000020562 0.000000171 0.000002356

O -0.000006206 -0.000020270 -0.000034227

H 0.000002160 0.000012066 0.000002337

H 0.000044493 0.000052735 0.000047390

C 0.000035440 -0.000072615 -0.000016989

H -0.000020164 -0.000007116 0.000013669

Os -0.000333505 -0.000011990 0.000160670

Amphi-Ru

O -0.000092589 0.000018587 -0.000084660

O -0.000003022 0.000080983 -0.000072116

O -0.000033921 0.000211952 0.000121755

O 0.000178081 -0.000000726 -0.000043265

O -0.000075697 -0.000067667 -0.000332185

N 0.000005725 -0.000044228 -0.000042227

C 0.000027380 -0.000048774 -0.000005730

O 0.000006646 0.000161114 0.000373339

C -0.000004076 0.000007643 -0.000012181

H -0.000019008 -0.000003400 -0.000035174

H -0.000023231 0.000010928 -0.000028345

H -0.000013220 -0.000007343 0.000015385

C 0.000013661 0.000038314 0.000019188

H 0.000053010 -0.000009002 0.000025156

H -0.000032482 -0.000009464 -0.000072122

C -0.000045506 -0.000017317 0.000019108

H 0.000009098 0.000003018 0.000007275

H -0.000002639 -0.000047360 -0.000045295

C 0.000029976 -0.000009320 -0.000040511

H -0.000002674 0.000020529 0.000021111

H -0.000009905 -0.000004163 0.000056764

C -0.000061683 -0.000010445 -0.000026404

H -0.000016469 -0.000083821 -0.000092470

C -0.000013896 -0.000035642 -0.000042172

H -0.000009227 0.000002797 -0.000002695

C 0.000007956 0.000158198 0.000083608

H 0.000007573 0.000008430 0.000009079

H -0.000022808 -0.000040753 0.000001944

C -0.000017044 0.000038236 -0.000072562

H 0.000009012 -0.000014756 -0.000015462

H -0.000011591 -0.000029257 0.000031995

C 0.000040860 -0.000048935 -0.000019674

H -0.000006569 0.000011438 -0.000007604

H -0.000008687 -0.000032788 -0.000050937

N -0.000029553 -0.000022151 -0.000036642

C 0.000065373 0.000102804 0.000107682

C -0.000014370 0.000047170 -0.000141906

H 0.000015733 -0.000045105 0.000105306

H -0.000026556 0.000021368 0.000059912

H 0.000001689 -0.000003749 -0.000013330

N -0.000027503 0.000005730 0.000019885

H 0.000023989 0.000048627 -0.000001074

H 0.000019806 0.000001116 -0.000009189

C 0.000011765 -0.000134269 0.000049343

O 0.000028159 0.000063786 0.000033411

N 0.000095844 -0.000010852 -0.000038340

H -0.000021675 -0.000013234 -0.000003419

C -0.000033743 0.000118774 0.000068553

O 0.000085943 -0.000020612 0.000039942

N -0.000052548 -0.000058846 -0.000065746

H 0.000050957 -0.000115037 -0.000124894

C -0.000065816 0.000030422 0.000013588

C -0.000029844 -0.000021188 -0.000045532

H 0.000059631 -0.000015953 0.000045982

H -0.000036154 0.000026257 0.000059719

O -0.000001377 -0.000020354 -0.000012197

H -0.000018514 0.000015405 -0.000012643

C 0.000077308 -0.000040239 -0.000058544

O 0.000062007 -0.000087231 0.000050320

N -0.000060345 0.000101778 0.000008915

H -0.000016206 -0.000002738 -0.000005483

C -0.000006022 0.000125329 0.000013790

H 0.000000005 0.000069816 0.000001395

H 0.000011506 -0.000061454 0.000010318

C -0.000018561 -0.000047411 -0.000005323

H 0.000001444 0.000014345 0.000006762

H -0.000017657 -0.000082272 -0.000049703

C -0.000003543 0.000005153 0.000013040

H -0.000003191 0.000036651 -0.000003019

H 0.000014165 -0.000031485 -0.000013997

C 0.000021643 -0.000061191 -0.000044794

C 0.000045025 -0.000032760 0.000049425

H -0.000036374 0.000031449 -0.000037447

H -0.000002435 0.000003416 0.000003540

H -0.000016640 0.000032025 -0.000041955

N 0.000047581 -0.000061360 0.000103750

C 0.000024726 0.000012725 -0.000011344

O -0.000028900 -0.000012275 0.000008942

O 0.000016517 -0.000004962 -0.000047397

H 0.000018929 0.000016793 0.000004734

H -0.000009255 0.000075041 0.000075664

C 0.000032929 -0.000075067 -0.000016638

H -0.000000147 -0.000006117 0.000002658

Ru -0.000118781 -0.000125074 0.000242066

Amphi-Sc

O -1.685628 -2.408690 -1.102574

O 1.190037 -4.007292 -0.097282

O 1.633628 -1.524548 1.385730

O 1.722784 -1.985105 -1.514373

O -0.334890 -0.313357 -1.291766

N -0.155587 -1.314227 2.683035

C 1.164906 -1.290632 2.537789

O -0.864586 -1.682105 1.583413

C 2.089402 -1.027696 3.689072

H 2.610697 -0.085138 3.519873

H 1.588633 -0.988203 4.652514

H 2.830891 -1.827889 3.708040

C -0.961925 -0.851894 3.811895

H -0.457889 -1.140066 4.733406

H -1.893202 -1.414323 3.751600

C -1.234540 0.665726 3.846626

H -1.552240 0.893047 4.868980

H -0.292977 1.204832 3.714051

C -2.335040 1.216450 2.923628

H -2.500841 2.269171 3.171030

H -3.262977 0.689078 3.152571

C -2.161835 1.080563 1.390817

H -1.854818 0.054259 1.182327

C -5.415944 0.655364 -0.569569

H -5.785198 1.662040 -0.352572

C -4.823562 0.726950 -1.998952

H -5.558092 1.260276 -2.607913

H -3.936098 1.358875 -1.976855

C -4.545384 -0.589796 -2.742256

H -5.501529 -1.110581 -2.842669

H -4.247704 -0.341057 -3.763461

C -3.614275 -1.658943 -2.163702

H -3.608790 -2.512216 -2.846445

H -4.014136 -2.020495 -1.216033

N -2.203985 -1.383260 -1.838749

C -1.407373 -0.332461 -1.973317

C -1.622115 0.777583 -2.957655

H -2.488926 0.649930 -3.593681

H -1.676166 1.732231 -2.433212

H -0.729034 0.807553 -3.584713

N -6.539494 -0.271857 -0.498960

H -6.204025 -1.147287 -0.104694

H -7.235409 0.070856 0.151919

C -4.334020 0.338753 0.485938

O -4.308684 -0.734497 1.068836

N -3.421335 1.319355 0.699832

H -3.442022 2.164013 0.145878

C -1.131364 2.034091 0.762848

O -1.491572 2.944134 0.008514

N 0.156498 1.812785 1.067798

H 0.384680 1.028275 1.654988

C 1.285654 2.497123 0.439990

C 1.229134 4.047209 0.609572

H 0.723222 4.259999 1.557197

H 2.241955 4.439755 0.674325

O 0.617891 4.693909 -0.480675

H -0.273698 4.309288 -0.553085

C 2.559042 1.997265 1.117944

O 2.571638 1.843681 2.333118

N 3.703136 1.830974 0.397933

H 4.472461 1.593791 1.006907

C 3.589577 0.270491 -1.586582

H 2.514428 0.127447 -1.517554

H 3.825864 0.271404 -2.652686

C 4.317389 -0.891755 -0.898280

H 5.400051 -0.726782 -0.960587

H 4.056569 -0.932704 0.161417

C 4.048845 -2.281278 -1.511959

H 4.100329 -2.225705 -2.603393

H 4.808513 -2.981100 -1.170524

C 2.406073 -3.872476 -0.441704

C 3.408608 -4.909775 -0.029166

H 3.705120 -4.740613 1.008726

H 2.918133 -5.881186 -0.075855

H 4.300062 -4.931479 -0.651295

N 2.740083 -2.809441 -1.158120

C 3.410580 2.774433 -1.912843

O 2.886751 2.608527 -2.980309

O 3.626251 3.990037 -1.376138

H 3.173492 4.640477 -1.938232

H 1.279466 2.275997 -0.629001

C 3.969779 1.649069 -1.033447

H 5.056020 1.773774 -1.116842

Sc 0.129112 -2.112551 -0.198378

Amphi-Ti

O 0.000000064 0.000003365 0.000005077

O 0.000002830 -0.000000201 0.000000730

O 0.000001607 -0.000001014 0.000005488

O 0.000000149 0.000003694 0.000001829

O -0.000003534 0.000002249 0.000000510

N 0.000002883 -0.000002126 0.000003241

C -0.000000513 0.000001302 -0.000002975

O -0.000000832 0.000003689 -0.000002791

C 0.000000420 -0.000001396 0.000000173

H -0.000001006 0.000000286 -0.000000329

H -0.000000023 0.000001034 -0.000000280

H -0.000000221 0.000001124 0.000000594

C 0.000000018 -0.000000229 -0.000000800

H 0.000000005 -0.000000703 -0.000000801

H -0.000000126 -0.000001024 0.000000287

C -0.000001425 -0.000000607 0.000000690

H -0.000000162 -0.000001026 0.000000417

H -0.000001812 -0.000000058 0.000000549

C -0.000000151 -0.000001411 -0.000000514

H -0.000001030 -0.000001605 0.000000039

H -0.000000426 -0.000001278 0.000000118

C -0.000001389 0.000000044 0.000000396

H 0.000001086 -0.000000827 0.000001286

C -0.000000266 -0.000002906 -0.000000489

H 0.000000261 -0.000002142 0.000000018

C 0.000001606 -0.000001810 -0.000000780

H 0.000000134 -0.000001477 0.000000430

H -0.000000780 -0.000001423 0.000000425

C 0.000000315 -0.000001324 0.000000131

H 0.000000921 -0.000001574 -0.000000364

H 0.000000459 -0.000000885 0.000000199

C 0.000000027 -0.000000490 -0.000000761

H 0.000001493 -0.000000969 0.000000295

H 0.000000681 -0.000001780 -0.000000365

N 0.000000005 -0.000002341 -0.000000517

C 0.000002590 -0.000001392 -0.000000924

C 0.000000610 -0.000000502 -0.000000711

H 0.000000083 -0.000000131 0.000000077

H -0.000000502 -0.000000349 -0.000000062

H -0.000000331 0.000000038 0.000000525

N 0.000001454 -0.000001448 0.000000529

H 0.000000418 -0.000002603 0.000000262

H -0.000000244 -0.000002656 -0.000000132

C -0.000001754 0.000002115 0.000001304

O 0.000000674 -0.000002484 -0.000001346

N 0.000001337 -0.000002804 0.000000214

H -0.000001608 -0.000001183 -0.000000646

C 0.000001247 -0.000004820 0.000000059

O -0.000001963 -0.000000305 0.000000974

N -0.000002180 0.000004291 0.000001287

H -0.000000475 -0.000002025 -0.000001336

C -0.000000065 0.000002571 -0.000001281

C -0.000000791 -0.000000613 -0.000002555

H -0.000001538 0.000000118 0.000000605

H -0.000001364 0.000000350 0.000001009

O -0.000000032 0.000000935 0.000002906

H -0.000003246 -0.000000391 -0.000001163

C -0.000002350 0.000002545 0.000006172

O -0.000001125 -0.000000075 -0.000002412

N -0.000002220 -0.000000102 -0.000000298

H -0.000001141 0.000002941 -0.000000938

C 0.000000181 0.000000777 0.000002438

H 0.000000741 0.000001200 0.000000634

H 0.000000673 0.000002526 -0.000000148

C -0.000002306 0.000000217 -0.000000176

H 0.000000511 0.000002021 0.000000599

H 0.000000542 0.000001527 -0.000000796

C 0.000002230 0.000002298 0.000000606

H 0.000001297 0.000001057 0.000000157

H 0.000002348 0.000001992 -0.000001217

C 0.000000229 0.000003200 0.000001903

C 0.000002915 0.000000457 -0.000000439

H 0.000000757 0.000001145 -0.000001171

H 0.000002089 0.000000388 -0.000000197

H 0.000001281 0.000001562 0.000000053

N 0.000001768 -0.000000574 -0.000000993

C -0.000003829 0.000002026 -0.000003122

O 0.000000661 0.000000553 0.000001370

O 0.000000065 0.000004076 0.000001926

H -0.000001634 -0.000000198 0.000000463

H 0.000000731 -0.000001181 0.000000680

C 0.000002540 0.000001479 -0.000004717

H -0.000001880 0.000000897 0.000000907

Ti 0.000001342 -0.000003626 -0.000012032

Amphi-V

O -1.475713000 -2.434143000 -1.149652000

O 1.033550000 -3.916060000 0.102709000

O 1.585479000 -1.454745000 1.284023000

O 1.620000000 -2.011439000 -1.455306000

O -0.290650000 -0.215574000 -1.195084000

N -0.218768000 -1.270723000 2.559432000

C 1.101250000 -1.264015000 2.439944000

O -0.914528000 -1.551908000 1.425308000

C 2.012302000 -1.057239000 3.612353000

H 2.544049000 -0.112719000 3.491320000

H 1.495315000 -1.052356000 4.568089000

H 2.746514000 -1.864252000 3.612813000

C -1.039197000 -0.841186000 3.691852000

H -0.566165000 -1.195104000 4.606767000

H -1.984965000 -1.370501000 3.580165000

C -1.266429000 0.681131000 3.802255000

H -1.586212000 0.867487000 4.832155000

H -0.308239000 1.197768000 3.703195000

C -2.340913000 1.307168000 2.896175000

H -2.458867000 2.360048000 3.168690000

H -3.290843000 0.815814000 3.112986000

C -2.160975000 1.186074000 1.363764000

H -1.843382000 0.163300000 1.152946000

C -5.384200000 0.668419000 -0.620300000

H -5.761919000 1.683321000 -0.464858000

C -4.769669000 0.665811000 -2.041594000

H -5.509158000 1.139837000 -2.692153000

H -3.903196000 1.324836000 -2.042069000

C -4.443666000 -0.684245000 -2.705358000

H -5.384788000 -1.235425000 -2.782290000

H -4.145059000 -0.484279000 -3.737430000

C -3.485946000 -1.699263000 -2.072775000

H -3.494287000 -2.598854000 -2.691776000

H -3.852210000 -1.992342000 -1.087506000

N -2.065652000 -1.390020000 -1.836697000

C -1.334409000 -0.283465000 -1.906969000

C -1.578825000 0.826348000 -2.886128000

H -2.418635000 0.658374000 -3.548804000

H -1.691215000 1.773931000 -2.359448000

H -0.670400000 0.900795000 -3.487263000

N -6.505396000 -0.258359000 -0.518863000

H -6.167590000 -1.121930000 -0.101350000

H -7.199019000 0.101745000 0.125319000

C -4.314798000 0.411167000 0.462357000

O -4.281394000 -0.643107000 1.079982000

N -3.416209000 1.408779000 0.657887000

H -3.441217000 2.241288000 0.086140000

C -1.123132000 2.135265000 0.747614000

O -1.467579000 3.043699000 -0.016128000

N 0.157670000 1.900536000 1.074043000

H 0.359226000 1.097644000 1.645625000

C 1.311387000 2.531482000 0.435438000

C 1.306494000 4.084266000 0.573368000

H 0.823001000 4.332262000 1.524186000

H 2.332136000 4.445491000 0.612534000

O 0.696213000 4.726400000 -0.520459000

H -0.205565000 4.362288000 -0.575809000

C 2.564183000 1.997021000 1.127019000

O 2.568534000 1.863885000 2.344413000

N 3.704589000 1.772110000 0.414861000

H 4.459841000 1.514831000 1.033242000

C 3.550878000 0.173215000 -1.538619000

H 2.472645000 0.067685000 -1.457328000

H 3.777765000 0.152100000 -2.606548000

C 4.249517000 -1.002025000 -0.840678000

H 5.336160000 -0.871582000 -0.915261000

H 3.995238000 -1.020520000 0.220891000

C 3.935028000 -2.392034000 -1.435208000

H 4.001059000 -2.356655000 -2.526638000

H 4.665380000 -3.114867000 -1.076287000

C 2.233004000 -3.873386000 -0.286570000

C 3.204486000 -4.935236000 0.143820000

H 3.683815000 -4.643556000 1.081487000

H 2.638294000 -5.846031000 0.328489000

H 3.977067000 -5.136133000 -0.595950000

N 2.609522000 -2.877062000 -1.084713000

C 3.454497000 2.673033000 -1.917500000

O 2.928418000 2.500915000 -2.983023000

O 3.709868000 3.892901000 -1.408713000

H 3.284855000 4.544889000 -1.990317000

H 1.300961000 2.287677000 -0.628834000

C 3.973185000 1.549947000 -1.010392000

H 5.063244000 1.638763000 -1.090509000

V 0.149747000 -2.009043000 -0.224262000

**Optimized geometry coordinates of different amphibactin-metal complexes at M06/def2tzvp level.**

**Amphibactin**

O 8.881544 -2.684188 -1.302474

O -9.396641 -1.604242 -0.365558

O -2.982489 3.041929 -1.485871

O -7.432627 0.321837 -0.685414

O 11.344081 -1.833962 -0.311194

N -0.760413 2.851953 -1.062734

C -1.963894 3.479490 -0.993085

O -0.734617 1.586314 -1.587898

C -1.953956 4.814709 -0.278001

H -1.562897 4.722268 0.736553

H -1.358062 5.558729 -0.814442

H -2.986946 5.153117 -0.227041

C 0.489555 3.298471 -0.485468

H 0.507984 4.389532 -0.538350

H 1.279924 2.944191 -1.157761

C 0.770310 2.837564 0.953490

H 1.099219 3.706562 1.536986

H -0.168020 2.495108 1.400400

C 1.845452 1.770311 1.080203

H 2.009138 1.547720 2.143860

H 2.792516 2.157335 0.684602

C 1.579705 0.428185 0.381986

H 1.355436 0.595777 -0.674088

C 5.107581 -0.935193 0.587773

C 6.266708 -1.252486 -0.347871

H 6.886854 -2.038909 0.091918

H 5.858725 -1.682684 -1.269956

C 7.171475 -0.088235 -0.713470

H 6.572146 0.712468 -1.160179

H 7.634664 0.318216 0.195597

C 8.290557 -0.460405 -1.677695

H 8.786459 0.458418 -2.009892

H 7.865544 -0.936996 -2.569068

N 9.258283 -1.416695 -1.166116

C 10.451717 -1.053582 -0.673472

C 10.723036 0.445480 -0.552859

H 9.876255 1.037603 -0.196604

H 11.543626 0.554780 0.157707

H 11.048554 0.879163 -1.506852

N 5.567062 -0.206440 1.780594

H 5.911720 0.702156 1.484506

H 4.767615 -0.019341 2.378673

C 3.957152 -0.143993 -0.039526

O 4.131467 0.728093 -0.877767

N 2.753116 -0.412909 0.507410

H 2.639048 -1.121326 1.224812

C 0.403924 -0.269369 1.083259

O 0.714958 -1.096020 1.982942

N -0.762498 0.175378 0.703765

H -0.835204 0.984975 -0.773767

C -1.976310 -0.137991 1.427807

C -1.862483 -0.291216 2.957209

H -1.175311 0.491579 3.318387

H -2.844413 -0.088128 3.397070

O -1.478796 -1.568851 3.373810

H -0.601693 -1.686037 2.944650

C -2.941864 1.027609 1.222887

O -2.595977 2.163458 1.516254

N -4.210717 0.774354 0.820183

H -4.748946 1.617867 0.671302

C -4.296985 -0.653435 -1.194050

H -4.200661 0.336591 -1.653356

H -3.285389 -1.076666 -1.166891

C -5.192277 -1.523866 -2.064068

H -4.644425 -1.734250 -2.992274

H -5.357686 -2.491411 -1.578241

C -6.534174 -0.916884 -2.463053

H -6.383859 0.107616 -2.822849

H -6.940607 -1.495294 -3.300004

C -8.493255 -1.710354 -1.198508

C -8.459761 -2.955597 -2.080748

H -9.122900 -3.685143 -1.615634

H -8.838118 -2.748588 -3.088622

H -7.467975 -3.401114 -2.186395

N -7.522894 -0.803756 -1.404581

C -4.684539 -1.633266 1.101342

O -4.527760 -2.764940 0.716766

O -4.841618 -1.361179 2.411357

H -4.685824 -2.202767 2.861197

H -2.408865 -1.076447 1.053162

C -4.787671 -0.420469 0.231167

H -5.884682 -0.188830 0.154826

H 4.701256 -1.889890 0.945281

**Amphi-Ga**

O -1.460014 -2.141957 -1.324761

O 1.048665 -3.689121 -0.130598

O 1.705557 -1.242235 0.979558

O 1.480025 -1.881801 -1.827723

O -0.172207 0.042991 -0.990933

N -0.004419 -1.602172 2.354172

C 1.279597 -1.337514 2.160906

O -0.765208 -1.852313 1.269476

C 2.219504 -1.184199 3.304128

H 2.620622 -0.168435 3.309678

H 1.775577 -1.403797 4.271958

H 3.054945 -1.867145 3.134491

C -0.773338 -1.413962 3.576856

H -0.232628 -1.871694 4.406747

H -1.691689 -1.985787 3.426014

C -1.084193 0.045179 3.907514

H -1.363726 0.073878 4.965642

H -0.164541 0.639197 3.842678

C -2.229767 0.694287 3.136692

H -2.439617 1.677789 3.571543

H -3.127349 0.087755 3.294485

C -2.108559 0.838263 1.614280

H -1.759375 -0.120657 1.212759

C -5.255167 0.555410 -0.454876

H -5.721646 1.457053 -0.040295

C -4.622199 0.982714 -1.788251

H -5.366260 1.593520 -2.308273

H -3.780480 1.648953 -1.581945

C -4.229974 -0.122257 -2.761473

H -5.151845 -0.644230 -3.038997

H -3.877112 0.340964 -3.687157

C -3.302656 -1.242220 -2.331398

H -3.213224 -1.962771 -3.149633

H -3.748495 -1.785982 -1.492499

N -1.939692 -0.991747 -1.858337

C -1.200431 0.095202 -1.731896

C -1.416996 1.359145 -2.484895

H -2.211880 1.316239 -3.221102

H -1.591172 2.181289 -1.787102

H -0.473180 1.574910 -2.992514

N -6.278068 -0.451348 -0.648411

H -5.871634 -1.359970 -0.439540

H -7.026575 -0.327282 0.019751

C -4.183811 0.116072 0.545665

O -4.048880 -1.043333 0.876353

N -3.394628 1.116924 1.011435

H -3.478863 2.044227 0.615432

C -1.150431 1.917701 1.129152

O -1.557607 2.891031 0.501601

N 0.148583 1.726985 1.402142

H 0.417219 0.839133 1.797217

C 1.196666 2.447294 0.703294

C 1.140412 3.974059 0.940037

H 0.660224 4.139785 1.913587

H 2.156730 4.366731 1.008140

O 0.508740 4.663742 -0.095545

H -0.388433 4.294630 -0.144243

C 2.531818 1.956378 1.223807

O 2.679799 1.761908 2.415851

N 3.581611 1.861929 0.367802

H 4.426515 1.602572 0.856324

C 3.234937 0.419408 -1.636755

H 2.174385 0.258693 -1.442165

H 3.327543 0.453690 -2.727023

C 4.063749 -0.731276 -1.086860

H 5.130789 -0.506776 -1.222362

H 3.889921 -0.842580 -0.009763

C 3.804850 -2.076175 -1.764179

H 3.835096 -1.961907 -2.853143

H 4.578726 -2.791667 -1.483662

C 2.235120 -3.581369 -0.536236

C 3.306503 -4.446956 0.030324

H 3.825426 -3.907421 0.829221

H 2.840375 -5.328844 0.464999

H 4.044098 -4.756618 -0.708827

N 2.515994 -2.645847 -1.430423

C 2.874236 2.896036 -1.777933

O 2.126408 2.741100 -2.698969

O 3.158488 4.086829 -1.247461

H 2.511240 4.719590 -1.599168

H 1.079976 2.259334 -0.370975

C 3.639438 1.774546 -1.087376

H 4.693145 1.969183 -1.333006

Ga 0.186630 -1.850257 -0.403029

Amphi-Co

O 1.348292 2.279593 -1.079820

O -0.855384 3.616754 0.210737

O -1.656512 1.260174 1.025117

O -1.264069 2.006487 -1.675990

O 0.166551 0.072034 -0.757887

N 0.049901 1.309619 2.450232

C -1.237871 1.169197 2.220097

O 0.843538 1.687078 1.422677

C -2.197348 0.958922 3.341045

H -1.907572 0.138779 3.996759

H -2.272953 1.872327 3.937316

H -3.171562 0.718424 2.922221

C 0.763635 1.026280 3.685687

H 0.150805 1.368523 4.522745

H 1.657781 1.653503 3.648369

C 1.135401 -0.440796 3.891726

H 1.442153 -0.533594 4.938619

H 0.237844 -1.064337 3.805380

C 2.280509 -0.993469 3.047614

H 2.536684 -1.994925 3.410431

H 3.161485 -0.365765 3.216444

C 2.108944 -1.039353 1.525099

H 1.754463 -0.052119 1.205977

C 5.158290 -0.573261 -0.653240

H 5.627160 -1.524170 -0.373357

C 4.452483 -0.828823 -1.993664

H 5.157188 -1.387633 -2.616901

H 3.607797 -1.501106 -1.824124

C 4.039439 0.384061 -2.821384

H 4.958520 0.913366 -3.093469

H 3.627767 0.024985 -3.769271

C 3.157391 1.472811 -2.239892

H 3.038198 2.267488 -2.981782

H 3.647452 1.929552 -1.373613

N 1.818544 1.182125 -1.730097

C 1.114374 0.083025 -1.615037

C 1.225235 -1.113884 -2.488783

H 1.961196 -1.016626 -3.279559

H 1.424276 -2.006639 -1.891548

H 0.238218 -1.249689 -2.941312

N 6.190096 0.434999 -0.781565

H 5.803857 1.321806 -0.466716

H 6.958238 0.235198 -0.155080

C 4.145118 -0.237004 0.443108

O 4.036614 0.886121 0.887892

N 3.371624 -1.275559 0.854192

H 3.431553 -2.162543 0.370956

C 1.117188 -2.064892 0.994484

O 1.476471 -2.984790 0.265172

N -0.168330 -1.879334 1.333924

H -0.402471 -1.029281 1.822137

C -1.250696 -2.476856 0.575122

C -1.294049 -4.014771 0.711033

H -0.829421 -4.274075 1.671522

H -2.331449 -4.352874 0.747958

O -0.692183 -4.667701 -0.367528

H 0.217380 -4.327967 -0.407177

C -2.553760 -1.907198 1.100717

O -2.699388 -1.739705 2.296393

N -3.583622 -1.691544 0.238909

H -4.408017 -1.382849 0.734159

C -3.147786 -0.174549 -1.695491

H -2.094838 -0.059835 -1.437588

H -3.185195 -0.172627 -2.789623

C -3.954963 0.997753 -1.156972

H -5.019491 0.839255 -1.377615

H -3.855800 1.060158 -0.067034

C -3.573799 2.355039 -1.751410

H -3.536094 2.292373 -2.844414

H -4.321004 3.104777 -1.485616

C -1.991471 3.676010 -0.336174

C -2.992570 4.666588 0.143485

H -3.624105 4.203139 0.907704

H -2.458248 5.492958 0.608896

H -3.631888 5.048270 -0.651220

N -2.278536 2.821973 -1.302553

C -2.904788 -2.655081 -1.945349

O -2.125760 -2.493702 -2.839709

O -3.263513 -3.854229 -1.485509

H -2.630549 -4.500175 -1.841222

H -1.101149 -2.230637 -0.482845

C -3.625539 -1.531449 -1.211543

H -4.684718 -1.666134 -1.474289

Co -0.197359 1.817780 -0.152502

Amphi-Cr

O -1.387814000 -2.218237000 -1.268127000

O 1.071890000 -3.712748000 -0.034038000

O 1.668917000 -1.310053000 1.075255000

O 1.442717000 -1.940937000 -1.800515000

O -0.196944000 -0.036224000 -0.882789000

N -0.057837000 -1.539037000 2.458806000

C 1.230798000 -1.325485000 2.265897000

O -0.805893000 -1.849823000 1.381717000

C 2.174487000 -1.133017000 3.397840000

H 2.628195000 -0.142479000 3.328031000

H 1.712787000 -1.250002000 4.375212000

H 2.971693000 -1.872247000 3.292377000

C -0.832454000 -1.296701000 3.669290000

H -0.291394000 -1.709339000 4.522336000

H -1.745061000 -1.883068000 3.540149000

C -1.156444000 0.173648000 3.925407000

H -1.456084000 0.249498000 4.975607000

H -0.238219000 0.768569000 3.848457000

C -2.289621000 0.777749000 3.101299000

H -2.521097000 1.775776000 3.489665000

H -3.184974000 0.167608000 3.258640000

C -2.123535000 0.860012000 1.578908000

H -1.766045000 -0.115578000 1.228619000

C -5.207232000 0.496452000 -0.577963000

H -5.684814000 1.411961000 -0.208863000

C -4.544226000 0.875009000 -1.911478000

H -5.274816000 1.470124000 -2.467703000

H -3.704745000 1.546055000 -1.712200000

C -4.136419000 -0.261361000 -2.842003000

H -5.054252000 -0.785009000 -3.128627000

H -3.755926000 0.174938000 -3.770114000

C -3.224133000 -1.373316000 -2.361592000

H -3.105870000 -2.108759000 -3.162522000

H -3.691326000 -1.902515000 -1.525005000

N -1.880753000 -1.099161000 -1.851387000

C -1.170819000 -0.001738000 -1.702891000

C -1.329245000 1.241067000 -2.501960000

H -2.084639000 1.180054000 -3.277907000

H -1.530203000 2.087864000 -1.841919000

H -0.357107000 1.429416000 -2.966741000

N -6.222205000 -0.521188000 -0.758333000

H -5.823086000 -1.415906000 -0.484653000

H -6.998059000 -0.362976000 -0.129963000

C -4.162040000 0.095696000 0.466261000

O -4.035573000 -1.051617000 0.838711000

N -3.389356000 1.114117000 0.923054000

H -3.465579000 2.028628000 0.497057000

C -1.148404000 1.920302000 1.087244000

O -1.535264000 2.882007000 0.429910000

N 0.145980000 1.730392000 1.390627000

H 0.401564000 0.838989000 1.788067000

C 1.198293000 2.424369000 0.670844000

C 1.162241000 3.955409000 0.884765000

H 0.671156000 4.143051000 1.848521000

H 2.182475000 4.336237000 0.960937000

O 0.552315000 4.638233000 -0.169508000

H -0.347408000 4.276383000 -0.227283000

C 2.534840000 1.923999000 1.178110000

O 2.700559000 1.750831000 2.370891000

N 3.569311000 1.796011000 0.306797000

H 4.418610000 1.536694000 0.787646000

C 3.199434000 0.345306000 -1.687059000

H 2.148003000 0.177678000 -1.451686000

H 3.250675000 0.381208000 -2.779888000

C 4.055038000 -0.802284000 -1.172037000

H 5.114395000 -0.575356000 -1.354359000

H 3.932069000 -0.911922000 -0.088437000

C 3.767195000 -2.154092000 -1.824494000

H 3.745914000 -2.053495000 -2.915020000

H 4.551157000 -2.868045000 -1.567961000

C 2.235418000 -3.634865000 -0.514248000

C 3.305493000 -4.549676000 -0.028672000

H 3.865027000 -4.059845000 0.774416000

H 2.830948000 -5.436714000 0.386699000

H 4.006871000 -4.844637000 -0.807855000

N 2.491740000 -2.705098000 -1.419675000

C 2.833316000 2.820093000 -1.832325000

O 2.056701000 2.657428000 -2.728407000

O 3.136387000 4.014339000 -1.322775000

H 2.463415000 4.641026000 -1.637649000

H 1.066490000 2.222698000 -0.398668000

C 3.611610000 1.702565000 -1.149220000

H 4.661906000 1.898179000 -1.408273000

Cr 0.233902000 -1.872394000 -0.282521000

Amphi-Fe(II)

Fe 0.432035000 -1.756323000 -0.278010000

O -1.243026000 -2.129099000 -1.226287000

O 2.021020000 -3.990168000 -0.072081000

O 1.941837000 -1.265100000 0.989601000

O 1.501575000 -2.079347000 -1.860728000

O -0.195179000 0.255681000 -0.994102000

N 0.274678000 -1.558336000 2.435718000

C 1.551806000 -1.294196000 2.181968000

O -0.520134000 -1.917728000 1.398460000

C 2.502854000 -1.047370000 3.309058000

H 2.213450000 -0.182238000 3.907571000

H 2.572975000 -1.922616000 3.959983000

H 3.481378000 -0.840678000 2.879466000

C -0.418993000 -1.438977000 3.696838000

H 0.287530000 -1.632792000 4.508204000

H -1.164851000 -2.239971000 3.702887000

C -1.087283000 -0.085869000 3.937192000

H -1.394038000 -0.077898000 4.989583000

H -0.335506000 0.706812000 3.846980000

C -2.334352000 0.244739000 3.119845000

H -2.813702000 1.128617000 3.558628000

H -3.046760000 -0.581524000 3.219969000

C -2.206870000 0.475483000 1.607892000

H -1.736155000 -0.419106000 1.176675000

C -5.300071000 0.016648000 -0.512886000

H -5.846049000 0.863591000 -0.076576000

C -4.686214000 0.536987000 -1.816880000

H -5.475391000 1.091359000 -2.335698000

H -3.909204000 1.267345000 -1.576558000

C -4.159899000 -0.495341000 -2.805851000

H -5.004564000 -1.126509000 -3.106183000

H -3.869965000 0.031435000 -3.719224000

C -3.082731000 -1.478346000 -2.380536000

H -2.866320000 -2.147771000 -3.221316000

H -3.476738000 -2.122800000 -1.586302000

N -1.800986000 -1.059094000 -1.825971000

C -1.190674000 0.115986000 -1.749594000

C -1.556479000 1.298275000 -2.591161000

H -2.336546000 1.131338000 -3.327540000

H -1.830371000 2.135992000 -1.942027000

H -0.641975000 1.597771000 -3.109311000

N -6.260562000 -1.038832000 -0.771236000

H -5.742150000 -1.906151000 -0.887165000

H -6.807352000 -1.194129000 0.068015000

C -4.236322000 -0.382256000 0.519151000

O -4.092039000 -1.529901000 0.879289000

N -3.509233000 0.658243000 0.998500000

H -3.629752000 1.573062000 0.582226000

C -1.370819000 1.678417000 1.209774000

O -1.878878000 2.659737000 0.669092000

N -0.058015000 1.603405000 1.485783000

H 0.293221000 0.680321000 1.704247000

C 0.881257000 2.467360000 0.797853000

C 0.697219000 3.954625000 1.164355000

H 0.232082000 3.996793000 2.158323000

H 1.673670000 4.436676000 1.241764000

O -0.037121000 4.667902000 0.207823000

H -0.873051000 4.175059000 0.122346000

C 2.284652000 2.059357000 1.195349000

O 2.545507000 1.810420000 2.357062000

N 3.264502000 2.103752000 0.253874000

H 4.153577000 1.830010000 0.646513000

C 2.902190000 0.734216000 -1.798469000

H 1.918739000 0.387805000 -1.468358000

H 2.829064000 0.843820000 -2.885669000

C 3.976899000 -0.281414000 -1.460092000

H 4.957354000 0.119586000 -1.762175000

H 4.014119000 -0.448174000 -0.376479000

C 3.789583000 -1.635635000 -2.138110000

H 3.516016000 -1.487083000 -3.190668000

H 4.737303000 -2.178229000 -2.125307000

C 2.951917000 -3.423528000 -0.633229000

C 4.382268000 -3.764454000 -0.270310000

H 4.874715000 -2.919494000 0.221037000

H 4.345955000 -4.597730000 0.429081000

H 4.989767000 -4.052888000 -1.131193000

N 2.765876000 -2.457885000 -1.552761000

C 2.294448000 3.187152000 -1.759790000

O 1.545047000 3.052666000 -2.682389000

O 2.467069000 4.351869000 -1.119968000

H 1.685206000 4.898102000 -1.308922000

H 0.711003000 2.339175000 -0.278174000

C 3.198144000 2.098744000 -1.204246000

H 4.205963000 2.409677000 -1.522652000

Amphi-Fe(III)

Fe 0.164150000 -1.896647000 -0.206863000

O -1.508138000 -1.991541000 -1.015601000

O 1.223574000 -3.845531000 -0.081170000

O 1.633205000 -1.374962000 1.045707000

O 1.150418000 -1.983675000 -1.807138000

O -0.177075000 0.156658000 -0.798928000

N -0.008664000 -1.722647000 2.492203000

C 1.261362000 -1.461534000 2.255387000

O -0.784043000 -2.001302000 1.416212000

C 2.245661000 -1.275702000 3.350662000

H 2.611186000 -0.246232000 3.341505000

H 1.843363000 -1.512611000 4.332906000

H 3.096398000 -1.928635000 3.146983000

C -0.766164000 -1.492320000 3.713513000

H -0.193773000 -1.886039000 4.555348000

H -1.665644000 -2.103486000 3.610487000

C -1.120934000 -0.029419000 3.971771000

H -1.422009000 0.030414000 5.022633000

H -0.213904000 0.583048000 3.901585000

C -2.265934000 0.564855000 3.155064000

H -2.547616000 1.528749000 3.593160000

H -3.137072000 -0.090142000 3.261446000

C -2.084516000 0.746138000 1.642972000

H -1.711744000 -0.198204000 1.234073000

C -5.095252000 0.534666000 -0.625434000

H -5.577518000 1.428130000 -0.211331000

C -4.378933000 0.995310000 -1.906818000

H -5.066756000 1.667412000 -2.428562000

H -3.513081000 1.607128000 -1.634883000

C -3.994537000 -0.077328000 -2.916472000

H -4.916641000 -0.573880000 -3.235354000

H -3.614346000 0.421890000 -3.811518000

C -3.078294000 -1.215134000 -2.503743000

H -2.869932000 -1.846944000 -3.372882000

H -3.597543000 -1.846867000 -1.777471000

N -1.813508000 -0.941995000 -1.821902000

C -1.054112000 0.140371000 -1.696778000

C -1.124411000 1.302357000 -2.623484000

H -1.862598000 1.215080000 -3.413849000

H -1.298420000 2.212096000 -2.042283000

H -0.131983000 1.408502000 -3.069535000

N -6.113358000 -0.455010000 -0.912600000

H -5.728266000 -1.371442000 -0.695820000

H -6.904597000 -0.337511000 -0.294419000

C -4.095556000 0.049333000 0.427415000

O -3.986011000 -1.121828000 0.717158000

N -3.345911000 1.032516000 0.991883000

H -3.409269000 1.971443000 0.619199000

C -1.122433000 1.850754000 1.224389000

O -1.518443000 2.837989000 0.614866000

N 0.174619000 1.680075000 1.541620000

H 0.449975000 0.757747000 1.844466000

C 1.193030000 2.424694000 0.821672000

C 1.166649000 3.935969000 1.145590000

H 0.701133000 4.054371000 2.132769000

H 2.188260000 4.313620000 1.221477000

O 0.525156000 4.690112000 0.159550000

H -0.366384000 4.311409000 0.081552000

C 2.550917000 1.882757000 1.210985000

O 2.795231000 1.632179000 2.376358000

N 3.518883000 1.792521000 0.262074000

H 4.396815000 1.493463000 0.661181000

C 2.958809000 0.483412000 -1.784813000

H 1.935394000 0.297286000 -1.451700000

H 2.904717000 0.600500000 -2.872142000

C 3.858236000 -0.692204000 -1.445417000

H 4.892039000 -0.450101000 -1.727572000

H 3.855932000 -0.877353000 -0.364375000

C 3.480092000 -1.988486000 -2.154916000

H 3.269252000 -1.796615000 -3.212606000

H 4.310079000 -2.694882000 -2.111254000

C 2.278464000 -3.627019000 -0.695171000

C 3.518792000 -4.415477000 -0.419151000

H 4.222612000 -3.813774000 0.163433000

H 3.239709000 -5.284566000 0.172387000

H 4.024621000 -4.744297000 -1.327141000

N 2.317394000 -2.636606000 -1.594310000

C 2.671518000 2.981562000 -1.748807000

O 1.868500000 2.912631000 -2.634061000

O 3.016658000 4.120120000 -1.149082000

H 2.344656000 4.783517000 -1.379539000

H 0.999335000 2.298226000 -0.249752000

C 3.446826000 1.794517000 -1.194392000

H 4.481600000 1.971150000 -1.522057000

Amphi-Mn

O -1.400621000 -2.233881000 -1.088164000

O 1.025402000 -3.671262000 0.097513000

O 1.710466000 -1.264716000 1.050744000

O 1.354022000 -1.973946000 -1.716316000

O -0.182316000 -0.054710000 -0.865779000

N 0.023848000 -1.393631000 2.480179000

C 1.312809000 -1.213502000 2.246115000

O -0.752788000 -1.757197000 1.432768000

C 2.262155000 -0.985831000 3.372663000

H 2.008737000 -0.099576000 3.954394000

H 2.273298000 -1.851940000 4.038826000

H 3.255864000 -0.832900000 2.958122000

C -0.700046000 -1.127004000 3.711342000

H -0.075809000 -1.445378000 4.549529000

H -1.575752000 -1.780118000 3.680921000

C -1.112169000 0.330318000 3.911544000

H -1.411986000 0.422056000 4.960510000

H -0.233008000 0.977221000 3.810099000

C -2.279791000 0.845627000 3.075335000

H -2.568976000 1.835627000 3.445055000

H -3.138714000 0.187486000 3.243238000

C -2.121611000 0.914564000 1.551758000

H -1.761997000 -0.063895000 1.213249000

C -5.208978000 0.497063000 -0.584999000

H -5.676756000 1.432353000 -0.255221000

C -4.540255000 0.810273000 -1.932009000

H -5.267173000 1.379975000 -2.518696000

H -3.699047000 1.487076000 -1.761088000

C -4.130000000 -0.370353000 -2.804861000

H -5.046364000 -0.906225000 -3.073122000

H -3.743040000 0.021326000 -3.750102000

C -3.219339000 -1.456996000 -2.265913000

H -3.089257000 -2.226209000 -3.032716000

H -3.692125000 -1.950677000 -1.411038000

N -1.885427000 -1.149345000 -1.754655000

C -1.165588000 -0.054983000 -1.677888000

C -1.321953000 1.153420000 -2.528458000

H -2.074690000 1.059905000 -3.303849000

H -1.529299000 2.024978000 -1.902375000

H -0.348305000 1.327700000 -2.995262000

N -6.236972000 -0.513524000 -0.726239000

H -5.839795000 -1.407938000 -0.448990000

H -6.994193000 -0.339222000 -0.079287000

C -4.165646000 0.125948000 0.471232000

O -4.035758000 -1.012020000 0.870051000

N -3.392957000 1.155993000 0.901931000

H -3.470698000 2.059584000 0.453232000

C -1.147382000 1.963581000 1.035438000

O -1.529904000 2.908539000 0.351675000

N 0.145176000 1.773746000 1.345223000

H 0.390281000 0.888888000 1.762911000

C 1.205580000 2.437148000 0.610540000

C 1.193538000 3.970642000 0.799700000

H 0.709295000 4.181468000 1.762205000

H 2.219312000 4.338523000 0.864717000

O 0.585772000 4.643663000 -0.262915000

H -0.316543000 4.287095000 -0.315415000

C 2.534379000 1.915017000 1.119078000

O 2.699144000 1.735181000 2.310027000

N 3.564460000 1.768636000 0.242899000

H 4.409827000 1.492369000 0.721371000

C 3.155041000 0.281798000 -1.718222000

H 2.109105000 0.124043000 -1.453000000

H 3.180874000 0.301382000 -2.812480000

C 4.011032000 -0.867850000 -1.208857000

H 5.066843000 -0.663256000 -1.433288000

H 3.923210000 -0.953065000 -0.119549000

C 3.676236000 -2.225609000 -1.827156000

H 3.625805000 -2.141674000 -2.918392000

H 4.453985000 -2.951406000 -1.584022000

C 2.162343000 -3.655897000 -0.432543000

C 3.220480000 -4.607641000 0.006459000

H 3.869002000 -4.121395000 0.741406000

H 2.737018000 -5.453051000 0.492133000

H 3.839353000 -4.966155000 -0.815317000

N 2.407734000 -2.753502000 -1.374048000

C 2.822820000 2.758442000 -1.909700000

O 2.035886000 2.589082000 -2.795754000

O 3.146126000 3.958234000 -1.426498000

H 2.475099000 4.586854000 -1.742060000

H 1.065489000 2.221009000 -0.454904000

C 3.591788000 1.643817000 -1.211292000

H 4.642141000 1.819524000 -1.484508000

Mn 0.218475000 -1.837947000 -0.212357000

Amphi-Mo

O -0.267376 -3.295166 -1.473143

O 1.965820 -3.464084 0.135556

O 1.710307 -1.018575 1.002515

O 2.131236 -1.655472 -1.681631

O -0.249273 -0.392037 -1.419508

N -0.076920 -1.584429 2.186318

C 1.186381 -1.184647 2.130821

O -0.683294 -1.853314 1.009921

C 1.986716 -0.992070 3.369979

H 2.328990 0.043372 3.423466

H 1.454437 -1.249204 4.282744

H 2.870018 -1.629677 3.285125

C -0.982602 -1.490378 3.328259

H -0.550182 -2.052354 4.158585

H -1.889705 -2.012431 3.016328

C -1.292299 -0.058874 3.760277

H -1.607726 -0.100454 4.807623

H -0.366841 0.529063 3.778693

C -2.406274 0.649876 2.998998

H -2.537360 1.656134 3.411535

H -3.334775 0.109376 3.202794

C -2.344710 0.741659 1.468909

H -2.030330 -0.228288 1.054556

C -5.620954 0.513907 -0.352181

H -6.025912 1.462251 0.024782

C -5.008141 0.826267 -1.724909

H -5.750584 1.409517 -2.277485

H -4.139903 1.481655 -1.594838

C -4.631971 -0.371189 -2.582662

H -5.551776 -0.911667 -2.834453

H -4.250294 -0.004064 -3.539982

C -3.689747 -1.413953 -1.986129

H -3.590899 -2.219202 -2.725799

H -4.197035 -1.887930 -1.138305

N -2.360129 -1.126326 -1.525700

C -1.507189 -0.274909 -1.888683

C -1.595580 0.904683 -2.809253

H -2.586281 1.044965 -3.230892

H -1.309888 1.805271 -2.258865

H -0.872922 0.783524 -3.618239

N -6.722593 -0.414526 -0.468482

H -6.355231 -1.354851 -0.575106

H -7.242099 -0.436077 0.400671

C -4.554110 0.083073 0.659025

O -4.522698 -1.017396 1.168744

N -3.649612 1.056085 0.938117

H -3.657663 1.894652 0.372075

C -1.395275 1.782917 0.890956

O -1.786121 2.619793 0.083610

N -0.116651 1.732820 1.285499

H 0.187567 1.045041 1.954979

C 0.901182 2.557050 0.673566

C 0.616676 4.074677 0.834284

H -0.031959 4.193110 1.712535

H 1.548609 4.603285 1.043965

O 0.075377 4.652131 -0.313349

H -0.739461 4.156006 -0.498351

C 2.219229 2.261700 1.355806

O 2.245824 2.019186 2.548550

N 3.368097 2.405460 0.646268

H 4.180561 2.278391 1.232187

C 3.452765 0.947588 -1.362208

H 2.426924 0.604238 -1.211879

H 3.587095 1.000346 -2.446543

C 4.442296 -0.040310 -0.768489

H 5.457952 0.368553 -0.852214

H 4.243402 -0.189016 0.300613

C 4.444785 -1.402502 -1.449733

H 4.479876 -1.292191 -2.537842

H 5.318334 -1.980322 -1.146766

C 3.118725 -3.107199 -0.206911

C 4.315021 -3.703179 0.452372

H 4.752530 -2.980608 1.147326

H 3.992299 -4.574560 1.017577

H 5.084433 -3.999202 -0.260147

N 3.265203 -2.180702 -1.139430

C 2.778973 3.354612 -1.581964

O 2.172158 3.095730 -2.578318

O 2.843366 4.571799 -1.041095

H 2.143919 5.103552 -1.456248

H 0.929947 2.330832 -0.399283

C 3.601945 2.344764 -0.792120

H 4.640707 2.679800 -0.919170

Mo 0.470044 -2.067182 -0.657304

Amphi-Os

O -1.200022 -2.246924 -1.174272

O 1.542578 -3.353180 0.337850

O 1.863546 -0.866498 1.278273

O 1.619817 -1.783333 -1.693633

O -0.321541 0.164524 -0.909763

N 0.073099 -0.973003 2.620793

C 1.370835 -0.770535 2.440373

O -0.690332 -1.332639 1.556143

C 2.243461 -0.435242 3.600731

H 3.238628 -0.213061 3.223355

H 1.888793 0.442619 4.140550

H 2.298108 -1.277239 4.295107

C -0.710989 -0.686690 3.810467

H -0.085671 -0.885542 4.682635

H -1.522799 -1.419197 3.808536

C -1.266092 0.735437 3.897829

H -1.571458 0.874901 4.939894

H -0.454299 1.455227 3.742137

C -2.486324 1.074916 3.042968

H -2.877159 2.046685 3.364719

H -3.268880 0.339270 3.256805

C -2.347616 1.079207 1.515444

H -1.895571 0.124590 1.231514

C -5.364862 0.176234 -0.571373

H -5.936010 1.072240 -0.300308

C -4.755760 0.464361 -1.950442

H -5.555070 0.898802 -2.558486

H -4.004005 1.249396 -1.842678

C -4.218918 -0.715573 -2.757156

H -5.071662 -1.369045 -2.969849

H -3.902229 -0.340662 -3.735110

C -3.175592 -1.662934 -2.191955

H -3.008625 -2.469523 -2.911431

H -3.555404 -2.137108 -1.280492

N -1.848883 -1.201913 -1.787108

C -1.306989 -0.011009 -1.702671

C -1.642946 1.152002 -2.565966

H -2.390585 0.946699 -3.323818

H -1.941966 2.004450 -1.952189

H -0.709325 1.434857 -3.060009

N -6.274588 -0.949004 -0.622531

H -5.754097 -1.788807 -0.381972

H -6.983403 -0.858123 0.093580

C -4.270656 0.005500 0.483593

O -3.979882 -1.085300 0.928428

N -3.638192 1.147953 0.862383

H -3.829508 2.014012 0.375617

C -1.467011 2.171906 0.936867

O -1.922313 3.051802 0.212323

N -0.162632 2.085289 1.244945

H 0.146295 1.252919 1.723600

C 0.858456 2.782235 0.489509

C 0.739896 4.317840 0.609352

H 0.247936 4.536160 1.566496

H 1.737217 4.759814 0.647509

O 0.077209 4.896297 -0.475492

H -0.796294 4.470513 -0.505781

C 2.209139 2.366299 1.038096

O 2.353295 2.196062 2.233169

N 3.275651 2.306217 0.194374

H 4.120810 2.090154 0.703934

C 3.107253 0.721569 -1.723488

H 2.080287 0.450105 -1.474094

H 3.157397 0.709254 -2.816874

C 4.087707 -0.295116 -1.156042

H 5.113889 0.043128 -1.354483

H 3.977831 -0.365517 -0.066703

C 3.955799 -1.702790 -1.739598

H 3.896276 -1.661855 -2.831969

H 4.828105 -2.302689 -1.475283

C 2.655013 -3.211896 -0.234751

C 3.839612 -3.966650 0.258816

H 4.426795 -3.328640 0.926173

H 3.482776 -4.817640 0.835855

H 4.487475 -4.316050 -0.544218

N 2.770101 -2.386516 -1.270113

C 2.498067 3.137258 -2.014940

O 1.742318 2.846586 -2.896009

O 2.701718 4.386288 -1.595149

H 1.980762 4.931955 -1.951872

H 0.749130 2.507015 -0.566642

C 3.364238 2.141950 -1.253905

H 4.395986 2.429499 -1.502344

Os 0.417266 -1.604419 -0.116265

Amphi-Ru

O 0 -1.258962 -2.304539 -1.263376

O 0 1.359984 -3.584078 0.203037

O 0 1.793179 -1.106878 1.218909

O 0 1.575860 -1.910669 -1.743271

O 0 -0.251583 0.053429 -0.929565

N 0 -0.009023 -1.278924 2.539592

C 0 1.293834 -1.087604 2.382277

O 0 -0.759755 -1.539394 1.443317

C 0 2.192709 -0.847309 3.542532

H 0 2.663447 0.130125 3.420659

H 0 1.690582 -0.885293 4.505739

H 0 2.978449 -1.605207 3.522584

C 0 -0.822954 -1.001811 3.716729

H 0 -0.287542 -1.352279 4.599529

H 0 -1.711267 -1.627545 3.599785

C 0 -1.209728 0.466401 3.895922

H 0 -1.502317 0.582990 4.944368

H 0 -0.318098 1.094475 3.780378

C 0 -2.380287 0.983602 3.061203

H 0 -2.646917 1.987248 3.410635

H 0 -3.246358 0.344623 3.262529

C 0 -2.243381 1.002391 1.533673

H 0 -1.847518 0.027975 1.230480

C 0 -5.303451 0.315651 -0.571595

H 0 -5.828174 1.232281 -0.276543

C 0 -4.676176 0.610930 -1.941373

H 0 -5.449498 1.105052 -2.537235

H 0 -3.882697 1.350243 -1.811396

C 0 -4.201570 -0.572830 -2.779703

H 0 -5.086719 -1.176400 -3.007007

H 0 -3.866268 -0.190636 -3.748473

C 0 -3.208932 -1.585595 -2.238518

H 0 -3.089324 -2.385844 -2.974183

H 0 -3.610229 -2.055996 -1.334266

N 0 -1.855096 -1.207919 -1.832928

C 0 -1.246583 -0.050352 -1.720368

C 0 -1.529915 1.152423 -2.549281

H 0 -2.285029 1.003273 -3.312915

H 0 -1.791183 1.998734 -1.910008

H 0 -0.583867 1.407869 -3.034468

N 0 -6.270671 -0.758331 -0.655905

H 0 -5.796478 -1.630884 -0.437384

H 0 -6.976359 -0.650165 0.060872

C 0 -4.224827 0.060321 0.482705

O 0 -4.001726 -1.052853 0.911147

N 0 -3.527158 1.156696 0.881604

H 0 -3.665166 2.040077 0.408135

C 0 -1.306803 2.058900 0.976444

O 0 -1.717750 2.969700 0.263631

N 0 -0.009155 1.914429 1.291504

H 0 0.267225 1.058868 1.749011

C 0 1.036361 2.602094 0.559972

C 0 0.955542 4.138742 0.710395

H 0 0.445354 4.350483 1.659252

H 0 1.963525 4.551055 0.785008

O 0 0.339926 4.760155 -0.377960

H 0 -0.546752 4.366797 -0.434706

C 0 2.374256 2.154933 1.113240

O 0 2.512609 2.004279 2.312403

N 0 3.436641 2.051348 0.271338

H 0 4.279539 1.824246 0.779234

C 0 3.179997 0.502347 -1.663585

H 0 2.143895 0.280686 -1.403676

H 0 3.214030 0.502115 -2.757492

C 0 4.113314 -0.570881 -1.122813

H 0 5.153693 -0.279070 -1.321278

H 0 4.006061 -0.657184 -0.034916

C 0 3.911571 -1.956229 -1.736599

H 0 3.876888 -1.889232 -2.828982

H 0 4.742463 -2.610570 -1.468820

C 0 2.494344 -3.454402 -0.322149

C 0 3.636847 -4.271672 0.175110

H 0 4.229199 -3.678311 0.878379

H 0 3.232110 -5.126645 0.713030

H 0 4.293439 -4.619401 -0.621539

N 0 2.678674 -2.580394 -1.307003

C 0 2.702476 2.950957 -1.925267

O 0 1.946625 2.714362 -2.822237

O 0 2.953541 4.179496 -1.471854

H 0 2.260220 4.763435 -1.822753

H 0 0.929376 2.351948 -0.502387

C 0 3.514960 1.900458 -1.178467

H 0 4.560320 2.134655 -1.425551

Ru 0 0.357573 -1.764087 -0.204031

Amphi-Sc

O -1.580502 -2.129927 -1.476163

O 1.411931 -3.870896 -0.407666

O 1.732202 -1.383596 0.978633

O 1.697046 -1.861782 -1.928438

O -0.268980 -0.035862 -1.115193

N 0.087236 -1.766318 2.418112

O 1.356171 -1.461683 2.177563

O -0.670673 -2.065889 1.351006

C 2.320554 -1.239908 3.290961

H 2.526270 -0.172560 3.403111

H 1.981996 -1.643645 4.243113

H 3.259030 -1.722246 3.011928

C -0.644697 -1.570274 3.660570

H -0.043384 -1.953591 4.486821

H -1.530723 -2.203006 3.574104

C -1.031783 -0.120661 3.943616

H -1.302340 -0.071354 5.003309

H -0.145905 0.519030 3.848149

C -2.218475 0.437562 3.164716

H -2.507947 1.400695 3.599908

H -3.068897 -0.236002 3.314590

C -2.104176 0.607280 1.643782

H -1.718425 -0.329502 1.222579

C -5.311867 0.370216 -0.356368

H -5.783067 1.212958 0.163413

C -4.754999 0.940811 -1.670467

H -5.530740 1.586769 -2.092538

H -3.910062 1.597924 -1.443681

C -4.394007 -0.060735 -2.757966

H -5.319831 -0.559849 -3.060979

H -4.063513 0.490006 -3.642953

C -3.451743 -1.205774 -2.447355

H -3.354252 -1.840049 -3.333801

H -3.889594 -1.835461 -1.666790

N -2.094981 -0.976421 -1.947782

C -1.319360 0.087672 -1.808375

C -1.548182 1.395644 -2.479022

H -2.382250 1.414414 -3.171852

H -1.669431 2.175232 -1.723439

H -0.629163 1.627502 -3.022781

N -6.307951 -0.655164 -0.593911

H -5.883267 -1.558868 -0.399671

H -7.077040 -0.563054 0.054754

C -4.183764 -0.124080 0.554582

O -4.010878 -1.301784 0.785992

N -3.403082 0.861681 1.060495

H -3.534201 1.811956 0.738954

C -1.198245 1.742975 1.182543

O -1.661661 2.725465 0.609954

N 0.113504 1.600013 1.417632

H 0.437781 0.718757 1.784463

C 1.115316 2.408360 0.748730

C 0.967823 3.922956 1.026627

H 0.488923 4.034522 2.008524

H 1.960146 4.373871 1.092334

O 0.287166 4.599461 0.014864

H -0.592230 4.188610 -0.024377

C 2.476302 1.986752 1.264484

O 2.628760 1.754957 2.448909

N 3.533275 1.996863 0.412378

H 4.392697 1.771095 0.892259

C 3.246032 0.570309 -1.608123

H 2.203377 0.334777 -1.387513

H 3.304115 0.627783 -2.699278

C 4.165130 -0.530270 -1.100174

H 5.209981 -0.208492 -1.210394

H 3.993322 -0.707706 -0.030965

C 4.030051 -1.861256 -1.836933

H 4.058795 -1.698234 -2.919912

H 4.860883 -2.517064 -1.574470

C 2.588544 -3.587788 -0.751458

C 3.739702 -4.389504 -0.243173

H 4.217252 -3.860730 0.587709

H 3.354989 -5.334889 0.133526

H 4.496837 -4.585733 -1.001767

N 2.790263 -2.542838 -1.539170

C 2.767182 3.034228 -1.714572

O 2.042565 2.865373 -2.651245

O 2.980686 4.224904 -1.150005

H 2.315162 4.833293 -1.509659

H 1.011459 2.248820 -0.331064

C 3.583975 1.937661 -1.043568

H 4.625514 2.185099 -1.293524

Sc 0.206460 -2.051531 -0.521206

Amphi-Ti

O -1.545803 -2.176041 -1.291198

O 1.156956 -3.827050 -0.298934

O 1.709692 -1.515920 1.155342

O 1.625919 -1.837343 -1.732508

O -0.250633 -0.049576 -1.157122

N -0.063196 -1.575422 2.474314

C 1.253687 -1.456336 2.319843

O -0.758537 -1.849864 1.352879

C 2.157631 -1.280507 3.488678

H 2.656789 -0.312631 3.409024

H 1.649216 -1.341144 4.448160

H 2.921899 -2.058376 3.442887

C -0.872849 -1.289559 3.648847

H -0.384943 -1.726056 4.522334

H -1.808305 -1.831551 3.490413

C -1.136622 0.194587 3.896737

H -1.428145 0.294241 4.947245

H -0.194491 0.750130 3.813692

C -2.251052 0.832235 3.073450

H -2.415300 1.857072 3.424691

H -3.175496 0.283282 3.280593

C -2.127113 0.843060 1.544176

H -1.810981 -0.155850 1.220314

C -5.324135 0.539390 -0.453372

H -5.763754 1.461513 -0.055022

C -4.727204 0.909504 -1.819274

H -5.476605 1.512291 -2.340556

H -3.867067 1.566503 -1.664562

C -4.379994 -0.242195 -2.754043

H -5.315225 -0.762841 -2.984896

H -4.043043 0.173328 -3.707893

C -3.451037 -1.346672 -2.290401

H -3.380767 -2.106350 -3.074701

H -3.878436 -1.847290 -1.416378

N -2.081259 -1.066561 -1.865319

C -1.311209 0.004115 -1.849939

C -1.534395 1.229968 -2.661560

H -2.371064 1.170568 -3.349421

H -1.645931 2.092033 -2.000717

H -0.616058 1.388559 -3.231508

N -6.369754 -0.454259 -0.582047

H -5.969438 -1.366137 -0.375641

H -7.083536 -0.307074 0.118738

C -4.231262 0.117673 0.533199

O -4.119418 -1.025706 0.923478

N -3.403081 1.118910 0.922535

H -3.469123 2.025324 0.478128

C -1.132589 1.850599 0.985713

O -1.501658 2.781852 0.275143

N 0.153312 1.657965 1.304097

H 0.415494 0.852274 1.850912

C 1.229409 2.368908 0.646914

C 1.168469 3.897568 0.881267

H 0.642161 4.060409 1.831684

H 2.180635 4.289431 0.998676

O 0.587041 4.590052 -0.180149

H -0.296842 4.203283 -0.293966

C 2.535364 1.864593 1.224530

O 2.609789 1.602718 2.411067

N 3.636840 1.835656 0.431985

H 4.454434 1.553841 0.953626

C 3.366848 0.423173 -1.596061

H 2.296652 0.269145 -1.447876

H 3.509037 0.457205 -2.680609

C 4.154703 -0.737772 -1.006517

H 5.228369 -0.510400 -1.057114

H 3.900839 -0.868350 0.052390

C 3.945319 -2.072889 -1.720471

H 4.019923 -1.934180 -2.805371

H 4.715215 -2.784599 -1.419586

C 2.344333 -3.703431 -0.677836

C 3.386095 -4.691624 -0.279113

H 3.918520 -4.328555 0.604643

H 2.887065 -5.621074 -0.011836

H 4.116101 -4.886961 -1.063829

N 2.653322 -2.654720 -1.432241

C 3.039964 2.907286 -1.733540

O 2.335238 2.769439 -2.689865

O 3.313261 4.090738 -1.179442

H 2.694321 4.732605 -1.563674

H 1.155004 2.181174 -0.431132

C 3.760783 1.770040 -1.020308

H 4.826556 1.955788 -1.213895

Ti 0.184064 -1.928557 -0.384832

Amphi-V

O -1.487083000 -2.231408000 -1.177862000

O 1.055892000 -3.789509000 -0.008160000

O 1.679998000 -1.328249000 1.077500000

O 1.569588000 -1.929355000 -1.621637000

O -0.276888000 -0.050763000 -1.087915000

N -0.017379000 -1.341181000 2.492016000

C 1.280086000 -1.218630000 2.267821000

O -0.778881000 -1.709787000 1.449145000

C 2.229157000 -1.001475000 3.397479000

H 1.973674000 -0.126014000 3.993529000

H 2.244285000 -1.878142000 4.050282000

H 3.223060000 -0.841747000 2.985610000

C -0.739402000 -1.015660000 3.711022000

H -0.129830000 -1.327273000 4.562316000

H -1.635387000 -1.641338000 3.694506000

C -1.105595000 0.459811000 3.862906000

H -1.393484000 0.600713000 4.909768000

H -0.208212000 1.074939000 3.729097000

C -2.264895000 0.976547000 3.016654000

H -2.509068000 1.996268000 3.335114000

H -3.145598000 0.362892000 3.232684000

C -2.127482000 0.955406000 1.488857000

H -1.795421000 -0.048786000 1.201373000

C -5.332783000 0.511725000 -0.475872000

H -5.777480000 1.457740000 -0.144518000

C -4.748130000 0.784268000 -1.868735000

H -5.516323000 1.321591000 -2.433037000

H -3.910146000 1.478539000 -1.770710000

C -4.372046000 -0.424682000 -2.717057000

H -5.296415000 -0.978215000 -2.911438000

H -4.042395000 -0.068798000 -3.697521000

C -3.423065000 -1.484193000 -2.190189000

H -3.344195000 -2.283809000 -2.932950000

H -3.838984000 -1.943848000 -1.288810000

N -2.054991000 -1.155112000 -1.794000000

C -1.319153000 -0.054224000 -1.796477000

C -1.568344000 1.137216000 -2.651943000

H -2.377072000 1.020728000 -3.365174000

H -1.739726000 2.011876000 -2.020990000

H -0.637572000 1.320115000 -3.194177000

N -6.365567000 -0.502994000 -0.523509000

H -5.960972000 -1.378121000 -0.198958000

H -7.106977000 -0.285123000 0.128093000

C -4.230395000 0.168087000 0.528944000

O -4.108606000 -0.949535000 0.986746000

N -3.402257000 1.192515000 0.847398000

H -3.484592000 2.082001000 0.372882000

C -1.136697000 1.955129000 0.912840000

O -1.509036000 2.893591000 0.213533000

N 0.150777000 1.729490000 1.207115000

H 0.375679000 0.865599000 1.677460000

C 1.246635000 2.388864000 0.525822000

C 1.217456000 3.925225000 0.695837000

H 0.713028000 4.139951000 1.647848000

H 2.240295000 4.297593000 0.779921000

O 0.630986000 4.583406000 -0.384362000

H -0.270131000 4.225505000 -0.449296000

C 2.541875000 1.893201000 1.138770000

O 2.615132000 1.719355000 2.339673000

N 3.644156000 1.774699000 0.350863000

H 4.453542000 1.519185000 0.898131000

C 3.370711000 0.260421000 -1.608536000

H 2.299292000 0.135965000 -1.451334000

H 3.509831000 0.249234000 -2.694213000

C 4.135252000 -0.895767000 -0.978752000

H 5.213966000 -0.706328000 -1.065374000

H 3.902026000 -0.964770000 0.090627000

C 3.870443000 -2.258261000 -1.624211000

H 3.937045000 -2.173548000 -2.714916000

H 4.620887000 -2.980604000 -1.299984000

C 2.227193000 -3.751114000 -0.444360000

C 3.241318000 -4.751566000 -0.003467000

H 3.836676000 -4.335878000 0.814426000

H 2.714297000 -5.625375000 0.374364000

H 3.917867000 -5.052774000 -0.802399000

N 2.562055000 -2.789407000 -1.300904000

C 3.091373000 2.734404000 -1.874185000

O 2.388227000 2.557780000 -2.825581000

O 3.385692000 3.941380000 -1.385584000

H 2.790811000 4.575869000 -1.816358000

H 1.178683000 2.159405000 -0.545206000

C 3.785059000 1.625179000 -1.093069000

H 4.856037000 1.782949000 -1.283657000

V 0.172601000 -1.894303000 -0.316279000

**Amphi-Fe^2+^-3H in water at B3LYP/6-31g(d,p)/LANL2DZ level**

Fe 2.167894000 0.721892000 1.369095000

O 3.744401000 0.116246000 0.211239000

O 1.662211000 2.231546000 0.248330000

O 0.746408000 0.959507000 2.673904000

O -0.082171000 2.334113000 -1.575713000

O 5.848625000 2.307404000 -1.542349000

N 0.821775000 -1.287125000 2.877796000

C 0.291749000 -0.098742000 3.205332000

O 2.016671000 -1.171813000 2.124106000

C -0.865418000 -0.007064000 4.152233000

H -1.797275000 0.058844000 3.577768000

H -0.932835000 -0.850402000 4.837820000

H -0.750974000 0.918909000 4.718101000

C 0.517771000 -2.648855000 3.363168000

H 0.199580000 -2.563557000 4.403243000

H 1.476142000 -3.173454000 3.365522000

C -0.541558000 -3.421731000 2.555651000

H -0.676176000 -4.364036000 3.097155000

H -1.510041000 -2.914385000 2.617775000

C -0.187309000 -3.770532000 1.098366000

H -0.843064000 -4.573938000 0.749236000

H 0.831237000 -4.170557000 1.060141000

C -0.269219000 -2.597424000 0.082360000

H 0.076640000 -1.670310000 0.540319000

C 2.526425000 -2.559320000 -2.509504000

H 1.841970000 -3.099379000 -3.179855000

C 2.614882000 -1.075234000 -3.032912000

H 2.147506000 -1.052631000 -4.021183000

H 1.992100000 -0.427968000 -2.405130000

C 4.012513000 -0.449621000 -3.173976000

H 4.617965000 -1.046994000 -3.864996000

H 3.884187000 0.525093000 -3.657714000

C 4.864496000 -0.254874000 -1.915893000

H 5.847999000 0.112841000 -2.214737000

H 5.003249000 -1.206384000 -1.394566000

N 4.348630000 0.739589000 -0.949036000

C 5.131955000 1.858226000 -0.656064000

C 5.007421000 2.490643000 0.706751000

H 5.142566000 1.756381000 1.505688000

H 5.766687000 3.268478000 0.782782000

H 4.018615000 2.943366000 0.824585000

N 3.837301000 -3.198858000 -2.402658000

H 3.719683000 -4.174278000 -2.135752000

H 4.269794000 -3.217042000 -3.322775000

C 1.858486000 -2.532573000 -1.142305000

O 2.495625000 -2.168956000 -0.108350000

N 0.567390000 -2.834719000 -1.090563000

H 0.077158000 -3.153714000 -1.921894000

C -1.684986000 -2.340667000 -0.468014000

O -2.057861000 -2.902985000 -1.507157000

N -2.420202000 -1.462815000 0.229268000

H -2.080841000 -1.110049000 1.112839000

C -3.707992000 -0.901873000 -0.182735000

C -4.845111000 -1.959466000 -0.264241000

H -4.743393000 -2.627721000 0.603439000

H -5.809475000 -1.450063000 -0.189447000

O -4.840596000 -2.648432000 -1.494997000

H -3.922853000 -2.941108000 -1.654095000

C -4.041295000 0.154587000 0.879882000

O -3.811742000 -0.099765000 2.065444000

N -4.647275000 1.311045000 0.502565000

H -4.885875000 1.893677000 1.296630000

C -3.211873000 2.473847000 -1.215699000

H -2.528428000 1.622040000 -1.280947000

H -3.289555000 2.889059000 -2.224770000

C -2.658422000 3.537697000 -0.256346000

H -3.359247000 4.380384000 -0.210134000

H -2.578396000 3.141257000 0.761539000

C -1.304193000 4.140007000 -0.661700000

H -1.299616000 4.446599000 -1.710993000

H -1.091102000 5.022262000 -0.060826000

C 0.750501000 3.125905000 0.435534000

C 0.769325000 4.016598000 1.636655000

H 0.686989000 3.386462000 2.525764000

H 1.736648000 4.525985000 1.669643000

H -0.026413000 4.757503000 1.652977000

N -0.168254000 3.224144000 -0.511872000

C -5.308378000 1.200021000 -1.915898000

O -4.884636000 1.098351000 -3.047618000

O -6.474642000 0.673412000 -1.507467000

H -6.873914000 0.215507000 -2.268527000

H -3.594231000 -0.470288000 -1.181045000

C -4.614350000 1.991683000 -0.799466000

H -5.255420000 2.871581000 -0.665328000

H 3.488560000 -0.806488000 -0.075736000

H 0.741835000 1.839890000 -1.377013000

H 2.010340000 -1.775834000 1.287464000

**Amphi-Fe^2+^-6H in water at B3LYP/6-31g(d,p)/LANL2DZ level**

Fe 3.229473000 -0.380627000 -1.171949000

O 4.570485000 -0.020226000 0.222587000

O 0.796762000 -2.792608000 0.028340000

O 1.849438000 -0.662238000 -2.533253000

O -1.322848000 -3.270703000 1.363025000

O 6.000500000 -2.891241000 1.655300000

N 1.595840000 1.573629000 -2.554509000

C 1.233164000 0.359495000 -2.973304000

O 2.808217000 1.561758000 -1.828579000

C 0.097000000 0.187793000 -3.931552000

H -0.765378000 -0.215407000 -3.392331000

H -0.195468000 1.109474000 -4.431114000

H 0.401473000 -0.550705000 -4.676365000

C 1.088939000 2.915229000 -2.930844000

H 0.977291000 2.931826000 -4.017195000

H 1.900515000 3.603967000 -2.689626000

C -0.233537000 3.361473000 -2.274305000

H -0.495200000 4.286911000 -2.798320000

H -1.031342000 2.654700000 -2.526075000

C -0.236333000 3.677555000 -0.764955000

H -1.103830000 4.306949000 -0.546588000

H 0.643284000 4.269491000 -0.509911000

C -0.277208000 2.419798000 0.163603000

H 0.030678000 1.537440000 -0.404854000

C 2.799286000 2.072376000 2.390517000

H 2.629059000 2.616049000 3.322010000

C 2.532022000 0.550860000 2.559923000

H 1.630459000 0.480515000 3.174494000

H 2.263135000 0.124593000 1.593461000

C 3.591012000 -0.349049000 3.219719000

H 3.818039000 0.010201000 4.229841000

H 3.113986000 -1.325910000 3.344267000

C 4.956427000 -0.570487000 2.549330000

H 5.475238000 -1.349239000 3.103787000

H 5.601492000 0.309789000 2.592491000

N 4.975093000 -0.981375000 1.122077000

C 5.654892000 -2.030388000 0.714959000

C 6.047345000 -2.264702000 -0.702200000

H 6.170398000 -1.315498000 -1.224558000

H 6.986686000 -2.821299000 -0.728632000

H 5.284829000 -2.858627000 -1.218667000

N 4.211834000 2.372090000 1.951942000

H 4.250692000 3.284612000 1.484733000

H 4.860577000 2.392353000 2.742400000

C 1.919357000 2.578395000 1.239541000

O 2.503140000 2.941533000 0.191990000

N 0.599670000 2.493736000 1.342138000

H 0.159862000 2.317516000 2.240192000

C -1.690901000 2.186819000 0.716018000

O -2.009317000 2.631414000 1.816215000

N -2.537331000 1.508415000 -0.112843000

H -2.090376000 0.908889000 -0.797180000

C -3.888048000 1.116822000 0.311995000

C -4.738339000 2.316563000 0.901588000

H -4.374283000 3.244782000 0.442756000

H -5.782676000 2.180800000 0.616102000

O -4.693356000 2.334270000 2.297373000

H -3.765399000 2.524037000 2.526677000

C -4.598126000 0.564269000 -0.893835000

O -4.608328000 1.240383000 -2.013307000

N -5.279465000 -0.550702000 -0.906837000

H -5.765593000 -0.730507000 -1.783211000

C -4.153008000 -2.409136000 0.409844000

H -3.367068000 -1.769337000 0.820241000

H -4.421258000 -3.111678000 1.203129000

C -3.645530000 -3.168181000 -0.823921000

H -4.469009000 -3.732513000 -1.276995000

H -3.280417000 -2.476259000 -1.589872000

C -2.557868000 -4.207127000 -0.521421000

H -2.895699000 -4.936051000 0.219770000

H -2.292960000 -4.763452000 -1.420173000

C -0.202217000 -3.320276000 -0.623714000

C -0.000609000 -3.549776000 -2.078338000

H 0.705377000 -2.802435000 -2.443519000

H 0.433138000 -4.546014000 -2.219483000

H -0.930010000 -3.487050000 -2.641579000

N -1.305473000 -3.639156000 0.017129000

C -6.066964000 -1.068849000 1.437345000

O -5.905244000 -1.605887000 2.507879000

O -6.855881000 -0.008609000 1.222653000

H -7.255102000 0.253199000 2.072239000

H -3.844936000 0.357473000 1.096300000

C -5.429008000 -1.592310000 0.138810000

H -6.182191000 -2.268436000 -0.279750000

H 4.526158000 1.669407000 1.254342000

H -1.308974000 -4.102284000 1.876051000

H 2.672979000 2.185926000 -1.025913000

H -4.067939000 2.049636000 -1.969467000

H 0.569886000 -2.685826000 0.975515000

H 6.513385000 -3.627926000 1.286987000

**Amphibactin-Fe(II)-4H in water at B3LYP/6-31g(d,p)/LANL2DZ level**

Fe 2.723653000 1.528683000 1.426395000

O 3.911510000 0.273617000 0.399780000

O 0.932009000 2.520586000 0.009017000

O 1.532502000 0.726275000 2.640083000

O -0.632582000 2.828597000 -1.932601000

O 5.846019000 2.323489000 -1.692969000

N 1.393864000 -1.534360000 2.917079000

C 0.961856000 -0.298472000 3.138560000

O 2.639170000 -1.656848000 2.317816000

C -0.250545000 -0.040924000 3.989631000

H -1.123915000 0.132611000 3.352455000

H -0.473219000 -0.853654000 4.678232000

H -0.064243000 0.872767000 4.557089000

C 0.793486000 -2.814681000 3.356158000

H 0.383429000 -2.684556000 4.358432000

H 1.646486000 -3.489901000 3.438541000

C -0.281676000 -3.408617000 2.427285000

H -0.542250000 -4.370378000 2.881230000

H -1.198399000 -2.812020000 2.476116000

C 0.132726000 -3.664475000 0.963087000

H -0.445009000 -4.502156000 0.560679000

H 1.182182000 -3.974054000 0.922385000

C -0.045178000 -2.458500000 -0.003749000

H 0.279903000 -1.533306000 0.473924000

C 2.705610000 -2.302226000 -2.661385000

H 1.935398000 -2.644348000 -3.368713000

C 3.180765000 -0.898603000 -3.159092000

H 2.970101000 -0.857017000 -4.232217000

H 2.553761000 -0.121382000 -2.707306000

C 4.666379000 -0.538864000 -2.983052000

H 5.297047000 -1.288546000 -3.471457000

H 4.841893000 0.399893000 -3.518371000

C 5.204050000 -0.383736000 -1.557609000

H 6.274584000 -0.160550000 -1.588926000

H 5.058704000 -1.301058000 -0.987028000

N 4.546287000 0.710342000 -0.814816000

C 5.050578000 1.986552000 -0.825491000

C 4.546866000 2.940508000 0.237176000

H 4.986292000 2.730143000 1.218047000

H 4.836871000 3.957216000 -0.032301000

H 3.439009000 3.010005000 0.246435000

N 3.824382000 -3.244753000 -2.539944000

H 3.463958000 -4.175607000 -2.337768000

H 4.281729000 -3.322991000 -3.445598000

C 2.019237000 -2.203677000 -1.302147000

O 2.612870000 -1.753658000 -0.279854000

N 0.760653000 -2.615527000 -1.213219000

H 0.280465000 -3.006859000 -2.018505000

C -1.497817000 -2.304483000 -0.490725000

O -1.873466000 -2.920495000 -1.499334000

N -2.273266000 -1.482432000 0.230543000

H -1.923655000 -1.072321000 1.085657000

C -3.656367000 -1.119221000 -0.076383000

C -4.629044000 -2.336301000 -0.088769000

H -4.342280000 -3.001925000 0.738573000

H -5.645518000 -1.981229000 0.099662000

O -4.648561000 -2.986228000 -1.339614000

H -3.717389000 -3.162376000 -1.575802000

C -4.068062000 -0.130630000 1.023449000

O -3.656018000 -0.302417000 2.172188000

N -4.948111000 0.865384000 0.723920000

H -5.218125000 1.395641000 1.544100000

C -3.962342000 2.253165000 -1.133112000

H -3.158200000 1.519988000 -1.269141000

H -4.216569000 2.632430000 -2.126489000

C -3.511175000 3.404585000 -0.224857000

H -4.314738000 4.146283000 -0.151218000

H -3.325321000 3.045272000 0.792398000

C -2.281162000 4.174141000 -0.716200000

H -2.382703000 4.507228000 -1.750377000

H -2.099318000 5.055907000 -0.101477000

C -0.159284000 3.225385000 0.252435000

C -0.288301000 3.815965000 1.604620000

H -0.102615000 3.031696000 2.342916000

H 0.487324000 4.581021000 1.719628000

H -1.265253000 4.257167000 1.780903000

N -1.031958000 3.366260000 -0.712221000

C -5.838486000 0.595709000 -1.608416000

O -5.478873000 0.511604000 -2.763335000

O -6.881956000 -0.075845000 -1.098030000

H -7.261438000 -0.621659000 -1.809687000

H -3.692191000 -0.671736000 -1.073710000

C -5.194290000 1.521047000 -0.566490000

H -5.969511000 2.266569000 -0.354935000

H 3.371691000 -0.538547000 0.076366000

H -1.230366000 2.078678000 -2.118526000

H 2.481945000 -1.881331000 1.363297000

H 0.933606000 2.217538000 -0.927886000
